# Targeting *Mycobacterium tuberculosis* Persistence through Inhibition of the Trehalose Catalytic Shift

**DOI:** 10.1021/acsinfecdis.4c00138

**Published:** 2024-03-14

**Authors:** Karishma Kalera, Rachel Liu, Juhyeon Lim, Rasangi Pathirage, Daniel H. Swanson, Ulysses G. Johnson, Alicyn I. Stothard, Jae Jin Lee, Anne W. Poston, Peter J. Woodruff, Donald R. Ronning, Hyungjin Eoh, Benjamin M. Swarts

**Affiliations:** † Department of Chemistry and Biochemistry, Central Michigan University, Mount Pleasant, Michigan 48859, United States; ‡ Biochemistry, Cell, and Molecular Biology Program, Central Michigan University, Mount Pleasant, Michigan 48859, United States; § Department of Molecular Microbiology and Immunology, Keck School of Medicine, University of Southern California, Los Angeles, California 90033, United States; ∥ Department of Pharmaceutical Sciences, University of Nebraska Medical Center, Omaha, Nebraska 68198, United States; ⊥ Department of Chemistry, University of Southern Maine, Portland, Maine 04104, United States

## Abstract

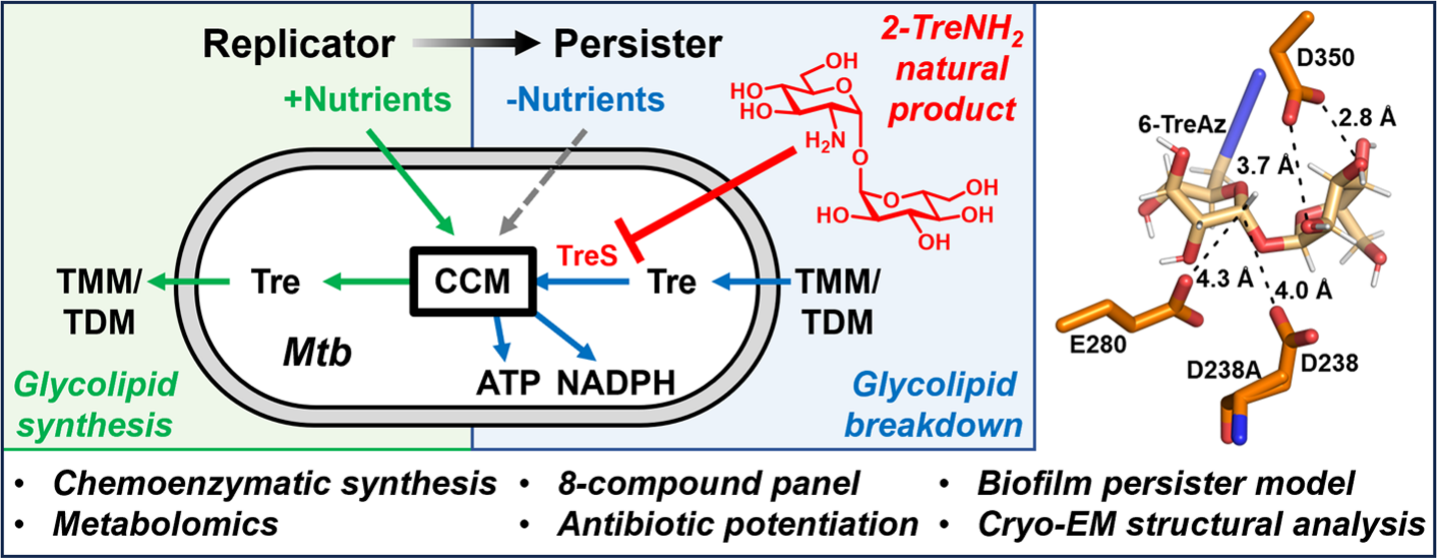

Tuberculosis (TB), caused by *Mycobacterium tuberculosis* (Mtb), is the leading cause of death worldwide by infectious disease. Treatment of Mtb infection requires a six-month course of multiple antibiotics, an extremely challenging regimen necessitated by Mtb’s ability to form drug-tolerant persister cells. Mtb persister formation is dependent on the trehalose catalytic shift, a stress-responsive metabolic remodeling mechanism in which the disaccharide trehalose is liberated from cell surface glycolipids and repurposed as an internal carbon source to meet energy and redox demands. Here, using a biofilm-persister model, metabolomics, and cryo-electron microscopy (EM), we found that azidodeoxy- and aminodeoxy-d-trehalose analogues block the Mtb trehalose catalytic shift through inhibition of trehalose synthase TreS (Rv0126), which catalyzes the isomerization of trehalose to maltose. Out of a focused eight-member compound panel constructed by chemoenzymatic synthesis, the natural product 2-trehalosamine exhibited the highest potency and significantly potentiated first- and second-line TB drugs in broth culture and macrophage infection assays. We also report the first structure of TreS bound to a substrate analogue inhibitor, obtained via cryo-EM, which revealed conformational changes likely essential for catalysis and inhibitor binding that can potentially be exploited for future therapeutic development. Our results demonstrate that inhibition of the trehalose catalytic shift is a viable strategy to target Mtb persisters and advance trehalose analogues as tools and potential adjunctive therapeutics for investigating and targeting mycobacterial persistence.

*Mycobacterium tuberculosis* (Mtb), which causes tuberculosis (TB), was responsible for 1.6 million deaths in 2021.^1^ TB endures as a leading cause of mortality in part due to the remarkable difficulty of treating Mtb infections. Conventional treatment of drug-susceptible Mtb infections requires the use of a multidrug cocktail administered for a minimum duration of six months, which increases the risk of deleterious side effects and patient nonadherence, and ultimately contributes to the emergence of multidrug-resistant (MDR) TB.^2−4^ This lengthy and intensive drug treatment regimen is necessitated by—and its efficacy is limited by—the ability of Mtb to form persister populations of cells, which exhibit extraordinary tolerance to TB drugs.^5,6^ Persisters form as a means for Mtb to respond and adapt to various stresses encountered during infection, thus promoting bacterial survival within the assaulting environment of the host. Given the urgent need to improve TB treatment, there has been increasing interest in understanding and targeting the molecular mechanisms that contribute to mycobacterial persistence.^6−8^ Advancements in these areas may lead to adjunctive therapeutics that shorten TB treatment durations and help to curb acquired drug resistance.

Using metabolomics in conjunction with an *in vitro* biofilm model of Mtb persistence, we recently discovered that Mtb persisters remodel trehalose metabolism to drive both transient drug tolerance and permanent drug resistance, an adaptive response mechanism termed the trehalose catalytic shift.^10^ Trehalose is a nonmammalian disaccharide consisting of two glucose residues linked by a 1,1-α,α-glycosidic bond.^11^ Trehalose has long been known to play unique and critical roles in mycobacteria, namely, its involvement in the biosynthesis of essential immunomodulatory glycolipids, including trehalose monomycolate (TMM) and trehalose dimycolate (TDM), which are components of the mycobacterial outer membrane, or mycomembrane.^12,13^ Actively replicating Mtb cells exhibit high glycolipid biosynthesis activity, whereas Mtb persister cells adapt to nutrient depletion by downregulating glycolipid synthesis, breaking down glycolipids to release free trehalose, which is recycled through central carbon metabolism (CCM) to support essential functions, including adenosine triphosphate (ATP) and nicotinamide adenine dinucleotide phosphate (NADPH) production (Figure 1A).^10,14−17^ The trehalose catalytic shift depends on the Mtb trehalose synthase TreS (Rv0126),^18^ which catalyzes the isomerization of trehalose to maltose, thus serving as a link between trehalose metabolism and CCM (Figure 1B). Although TreS is not strictly essential for Mtb viability under favorable growth conditions, it is required for the formation of biofilm persisters and can therefore, in principle, be conditionally targeted as a persistence factor. On the basis that our prior work had identified several monosubstituted trehalose analogues as selective inhibitors of biofilm formation in the nonpathogenic model organism *Mycobacterium smegmatis* (Msmeg),^19^ we then investigated the influence of 6-azido-6-deoxy-d-trehalose (6-TreAz) on Mtb persister formation. Our initial studies revealed that 6-TreAz interrupted the trehalose catalytic shift through inhibition of TreS and that 6-TreAz significantly sensitized Mtb to treatment with bedaquiline (BDQ), a drug that was recently approved by the FDA to treat MDR-TB.^10^ Thus, small-molecule TreS inhibitors, including trehalose analogues, are promising tools for interrogating the trehalose catalytic shift and represent lead compounds for the development of adjunctive therapeutics for TB.

**Figure 1:**
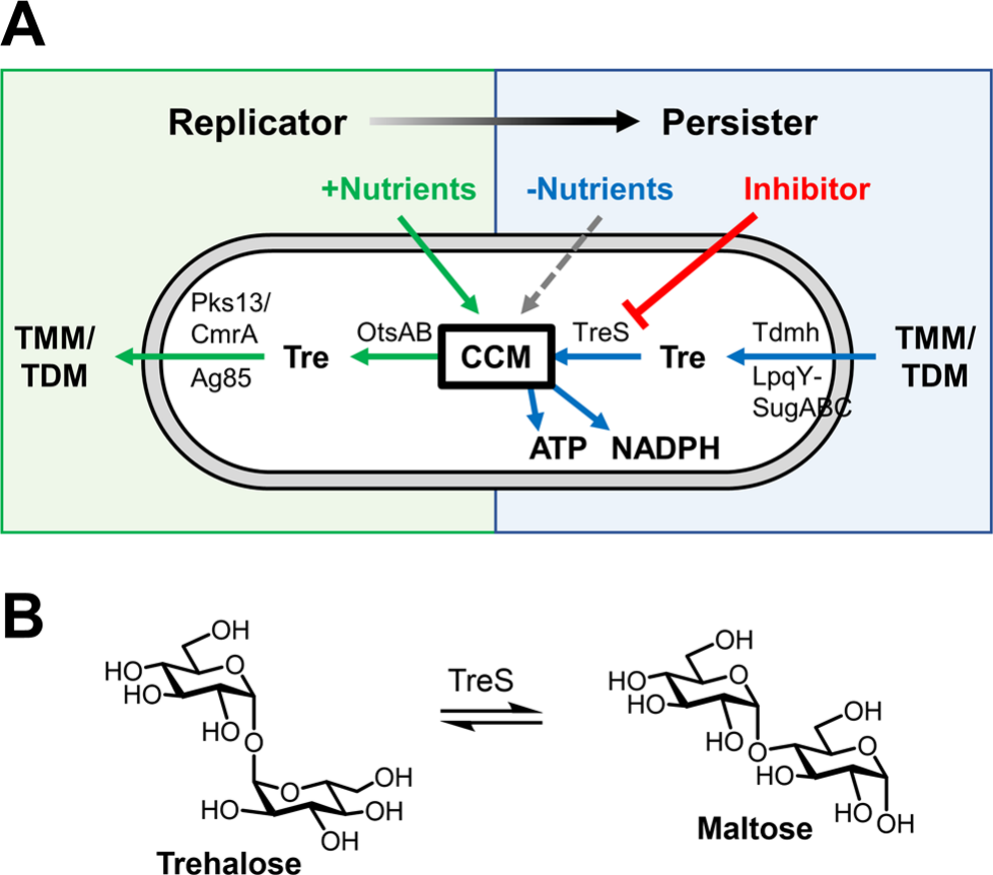
(A) Overview of the TreS-mediated trehalose (Tre) catalytic shift in Mtb. Under nutrient-replete conditions (left, green), actively replicating Mtb uses exogenously acquired nutrients to drive the synthesis of trehalose via OtsAB, and the subsequent synthesis of cell surface TMM and TDM via Pks13/CmrA and Ag85. In response to stresses such as nutrient-scarce conditions encountered during biofilm formation or antibiotic treatment (right, blue), Mtb degrades cell surface TMM/TDM via Tdmh and potentially other hydrolases, releasing free trehalose that is recycled via LpqY-SugABC and channeled into central carbon metabolism (CCM) via TreS, supporting ATP and NADPH synthesis. The present study investigates inhibition of the TreS-mediated trehalose catalytic shift. (B) TreS-catalyzed conversion of trehalose to maltose.

Over half a century ago, the trehalosamine natural product 2-amino-2-deoxy-d-trehalose (2-TreNH_2_) was isolated from *Streptomyces* and shown to potently inhibit Mtb growth.^20,21^ However, the intriguing activity of 2-TreNH_2_ on Mtb growth has not been further investigated, likely in part due to the difficulty of isolating or synthesizing this molecule.^22,23^ Thus, the mechanism of action and therapeutic potential of 2-TreNH_2_ and related compounds remains untested. In light of this history and the confluence of (i) our discovery of the trehalose catalytic shift as a persistence factor in Mtb, (ii) our finding that 6-TreAz inhibits the trehalose catalytic shift and enhances TB drug efficacy, and (iii) our lab’s ongoing development of novel chemoenzymatic methods to efficiently synthesize trehalose analogues,^24,25^ here we synthesized a panel of azidodeoxy- and aminodeoxy-d-trehalose (TreAz and TreNH_2_) analogues and investigated them as potential inhibitors of the Mtb trehalose catalytic shift. Using our established biofilm model, metabolomics, and cryo-electron microscopy (EM), we found that a subset of the compounds impact Mtb growth and trehalose metabolism, with 2-TreNH_2_ potently blocking the TreS-mediated trehalose catalytic shift, as well as potentiating front-line TB drugs both in liquid culture and in infected macrophages. Consistent with the cell-based results, the cryo-EM structure of TreS-D238A in complex with 6-TreAz shows that the inhibitor binds within the active site and induces a conformational change essential for conversion of trehalose to maltose, insights that may aid therapeutic development. Our data generate renewed interest in trehalosamines as TB therapeutic candidates and demonstrate that inhibition of the trehalose catalytic shift is a promising strategy to target mycobacterial persisters.

## Results and Discussion

### Design and Synthesis of Azido Trehalose (TreAz) and Trehalosamine (TreNH_2_) Analogues

**Scheme 1:**
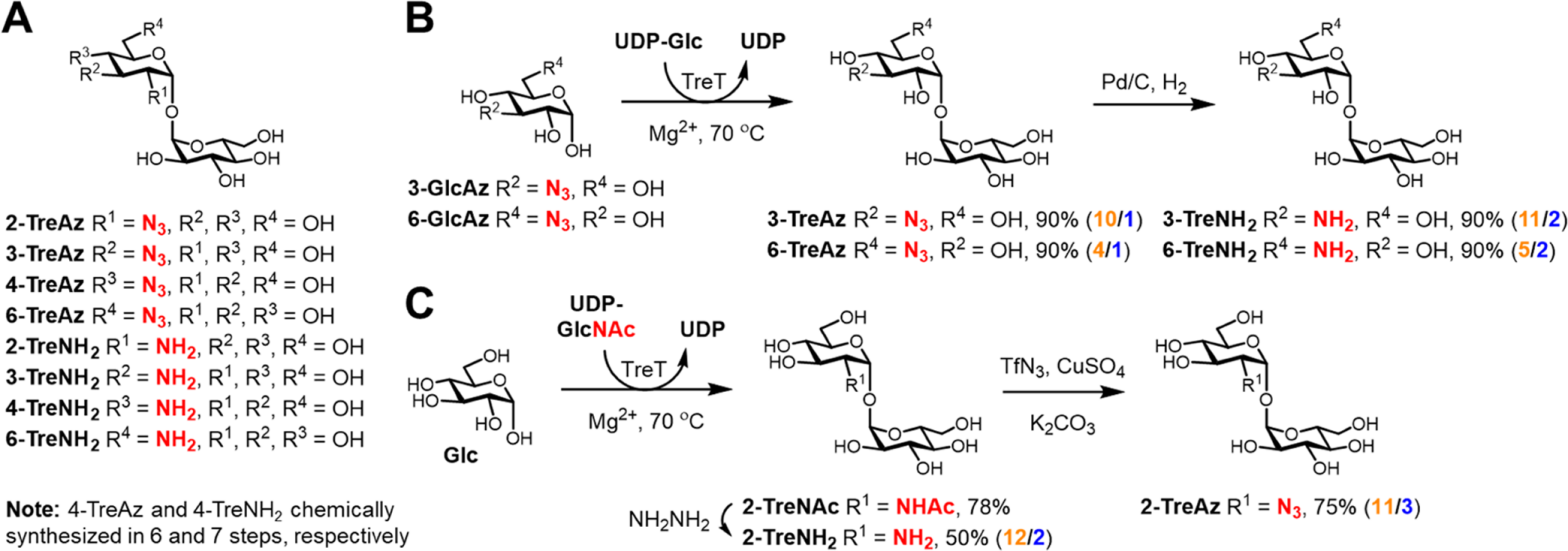
(A) TreAz and TreNH_2_ Analogues Investigated in This Study. (B, C) TreT-Mediated Chemoenzymatic Synthesis of (B) 3- and 6-Position-Modified Compounds and (C) 2-Position-Modified Compounds Comparison of chemoenzymatic synthesis step counts (blue) and chemical synthesis step counts (orange) is given for each target compound.

Prior studies from our group and others established that the synthetic compound 6-TreAz inhibits growth of Msmeg,^19^ Mtb,^10,26^ and *Mycobacterium aurum*.^27^ Importantly, we recently demonstrated that 6-TreAz is a selective inhibitor of biofilm-associated persisters in Msmeg and Mtb, and that 6-TreAz inhibits the TreS-mediated trehalose catalytic shift in Mtb.^10,19^ In addition, the natural product 2-TreNH_2_ was shown in 1957 to inhibit Mtb growth,^20,21^ and aside from synthetic studies,^22,28^ to our knowledge it has not been further studied since, aside from synthetic studies. To investigate 6-TreAz and 2-TreNH_2_ in more depth, identify related active compounds, and enable an initial structure–activity relationship study for this underexplored compound class, we designed a panel of 8 regioisomeric TreAz and TreNH_2_ analogues with systematic variation of the azidodeoxy or aminodeoxy group position (Scheme 1A). In addition to 6-TreAz and 2-TreNH_2_, the remaining target compounds have previously been synthesized and/or isolated from natural sources. In 2012, the TreAz series was chemically synthesized, including 2-, 3-, 4-, and 6-TreAz, and the analogues were tested for their ability to metabolically label TMM and TDM in mycobacteria to allow click chemistry-based imaging and other applications.^29^ Out of the four trehalosamine targets, 2-, 3-, and 4-TreNH_2_ are naturally occurring aminoglycosides isolated from *Streptomyces* (2- and 4-TreNH_2_) or *Nocardiopsis* (3-TreNH_2_), and 6-TreNH_2_ was chemically synthesized, first by the Hanessian group.^20,30−32^ However, aside from our prior study on 6-TreAz, the remaining 7 compounds in the panel have not been tested for their impact on Mtb biofilm formation or the trehalose catalytic shift.

To access the 8-member trehalose analogue panel, we used a combination of chemoenzymatic and chemical synthesis. Despite the relatively simple appearance of the TreAz and TreNH_2_ target compounds, their preparation using conventional chemical synthesis approaches poses multiple challenges due to trehalose’s unique 1,1-α,α-glycosidic bond, eight similarly reactive hydroxyl groups, and *C*_2_-symmetric arrangement.^33,34^ Previously reported chemical syntheses of several of the target compounds required between 4 and 12 steps and generally proceeded in <10% overall yield. Similarly, isolation of naturally occurring trehalosamines is lengthy and arduous. We previously developed a chemoenzymatic method for trehalose analogue synthesis, which utilizes the substrate-promiscuous trehalose synthase TreT from *Thermoproteus tenax* to convert glucose analogues and UDP-glucose into their respective trehalose analogues in one step.^35,36^ Here, we applied TreT catalysis to generate 6 out of the 8 target compounds (Scheme 1B,C). Commercially available 3- and 6-azido glucose analogues were reacted with UDP-glucose via TreT catalysis to generate 3- and 6-TreAz in one step, each in ≥90% yield. Then, Pd-catalyzed reduction converted 3- and 6-TreAz to 3- and 6-TreNH_2_ in 99 and 88% yield, respectively. Previously, we found that various 2-*N*-substituted glucoses were not tolerated by TreT, so we developed an alternative route whereby native glucose was successfully reacted with UDP-*N*-acetylglucosamine via TreT catalysis, generating 2-*N*-acetyltrehalosamine that could then be *N*-deacetylated with hydrazine to give 2-TreNH_2_; subjecting 2-TreNH_2_ to diazo transfer reaction with triflic azide gave 2-TreAz.^22^ Here, to access 2-TreAz and 2-TreNH_2_, we used a combination of this chemoenzymatic procedure^22^ to support initial screening experiments and a reported chemical synthesis procedure^29^ to support experiments requiring more material. Because *T. tenax* TreT does not tolerate 4-position-modified glucose analogues, we chemically synthesized 4-TreAz and 4-TreNH_2_ using a reported route.^37^ Comparison of step counts for TreT chemoenzymatic synthesis and chemical synthesis, shown in Scheme 1, reveals the improved efficiency of TreT catalysis.

### TreAz and TreNH_2_ Analogues Differentially Inhibit Planktonic and Biofilm Growth of Mycobacteria via Action on Trehalose Metabolism

**Figure 2:**
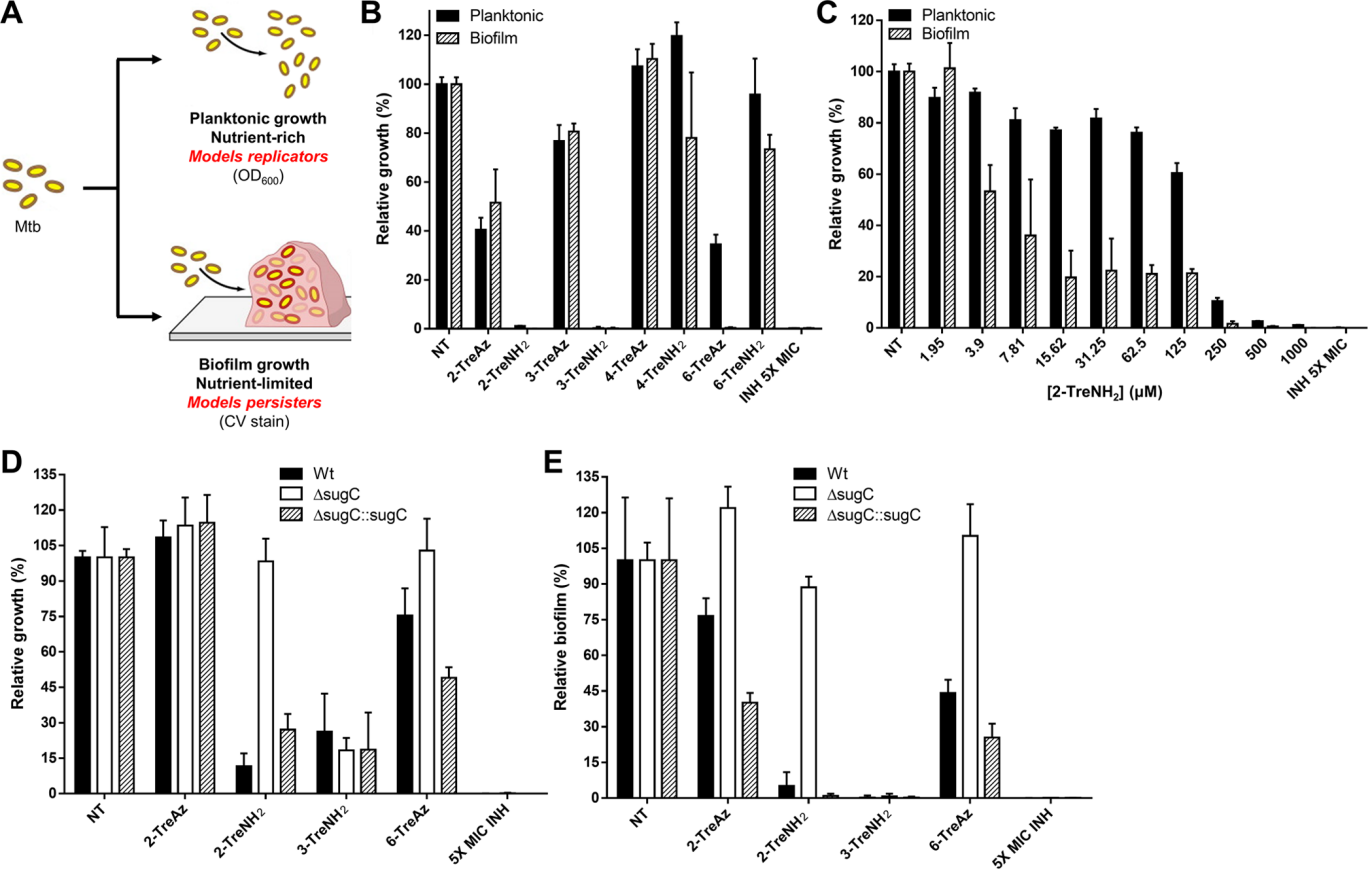
6-TreAz and 2-TreNH_2_ selectively inhibit biofilm formation in Mtb in an LpqY-SugABC-dependent manner. (A) Scheme for evaluating planktonic and biofilm growth in Mtb. Reproduced with permission from ref (39). Copyright 2005 Elsevier. (B–E) Mtb H37Rv wild type (or mutant, if indicated) was cultured under planktonic or biofilm growth conditions in the presence of the indicated trehalose analogue, or left not treated (NT) as negative control, or treated with front-line TB drug isoniazid (INH) as positive control. Growth was measured using OD_600_ reading for planktonic conditions and CV staining for biofilm conditions. In (B), all analogue concentrations are 1 mM. In (D), analogue concentrations are 2-TreAz, 1000 μM; 2-TreNH_2_, 250 μM; 3-TreNH_2_, 1000 μM; 6-TreAz, 1000 μM. In (E), analogue concentrations are 2-TreAz, 1000 μM; 2-TreNH_2_, 250 μM; 3-TreNH_2_, 1000 μM; 6-TreAz, 250 μM. Data are normalized relative to positive control at 100%. Error bars represent the standard deviation of three replicates and data are representative of three independent experiments. See Figures S1–S16 for additional Mtb and Msmeg data.

We previously used *in vitro* mycobacterial biofilm culture, which is a model for drug-tolerant persisters, to discover the trehalose catalytic shift and identify 6-TreAz as a selective biofilm inhibitor that interferes with this pathway.^10^ To determine whether the synthesized trehalose analogues were selective biofilm inhibitors, we evaluated their effects on planktonic (i.e., free-living in liquid medium) and biofilm-associated mycobacterial growth. The compound panel was screened at 1 mM concentration for inhibition of Msmeg and Mtb grown under either planktonic or biofilm conditions and growth was monitored by optical density at 600 nm (OD_600_) or crystal violet staining, respectively (Figure 2A). In the model organism Msmeg, 6-TreAz exhibited selective albeit partial biofilm inhibition at 1 mM, consistent with our reported results, while 2-TreAz and 2-TreNH_2_ promisingly showed selective and complete biofilm inhibition at the same concentration (Figure S1). None of the 3- or 4-position-modified analogues, nor 6-TreNH_2_, exhibited inhibition of Msmeg in either growth mode at 1 mM. This finding demonstrates that subtle changes in modification type (azido or amino) and position on the disaccharide can influence activity. Dose–response curves in Msmeg revealed that the natural product 2-TreNH_2_ exhibited the highest potency, with selective biofilm inhibition occurring in the low-micromolar range (Figures S2–S9).

Inhibitory activities of TreAz and TreNH_2_ analogues in the pathogen Mtb were largely consistent with those observed in Msmeg, with some notable differences (Figure 2B,C; Figures S10–S16). 6-TreAz selectively inhibited biofilm formation in Mtb, with modestly higher potency than in Msmeg, consistent with our prior work on this compound. 2-TreAz was less potent in Mtb than in Msmeg. 2-TreNH_2_, the most potent compound in the panel against Mtb, fully inhibited both growth modes at 1 mM and dose–response analysis revealed selective, low-micromolar biofilm inhibition (Figure 2C). Also consistent with the results in Msmeg, 3-TreAz, 4-TreAz, 4-TreNH_2_, and 6-TreNH_2_ lacked activity in Mtb. By contrast, 3-TreNH_2_, which had no effect in Msmeg, completely blocked Mtb planktonic and biofilm growth at 1 mM but had lower potency than 6-TreAz and 2-TreNH_2_ and was not selective for biofilm inhibition, suggesting an alternative mechanism of action. These data show that the activity of the compound panel was similar in Msmeg and Mtb and that modifications at the 2- and 6-positions were most likely to confer inhibitory activity. The natural product 2-TreNH_2_ was identified as the most potent selective biofilm inhibitor in both Msmeg and Mtb.

In a previous study, we found that the inhibitory activity of 6-TreAz in Msmeg was completely dependent on LpqY-SugABC,^19^ a plasma membrane-associated trehalose-specific transporter. The trehalose transporter is conserved in mycobacteria and required for virulence of Mtb.^38^ However, it is dispensable for growth and biofilm formation, and thus the genes encoding it can be knocked out.^38^ To test whether the inhibitory action of TreAz and TreNH_2_ analogues requires the trehalose transporter, we assessed their effects on planktonic and biofilm growth in Msmeg and Mtb wild type, ΔsugC knockout mutants (lack functional transporter), and ΔsugC::sugC complements (transporter restored). The activity of 6-TreAz, 2-TreAz, and 2-TreNH_2_ against planktonic and biofilm growth in wild-type Mtb was reversed in the ΔsugC mutant and restored in the ΔsugC::sugC complement, demonstrating that LpqY-SugABC is required for inhibition by these compounds (Figure 2D,E). Identical results were obtained for these compounds in Msmeg (Figures S17 and S18). These findings confirm that the compounds interfere with trehalose metabolism and, furthermore, suggest that the compounds require active transport into the cell to act on an intracellular target. Of note, the inhibitory activity of 3-TreNH_2_ in Mtb was independent of LpqY-SugABC, further suggesting an alternative mechanism of action for this analogue.

### TreAz and TreNH_2_ Analogues Inhibit the TreS-Mediated Trehalose Catalytic Shift in *Mycobacterium tuberculosis*

**Figure 3:**
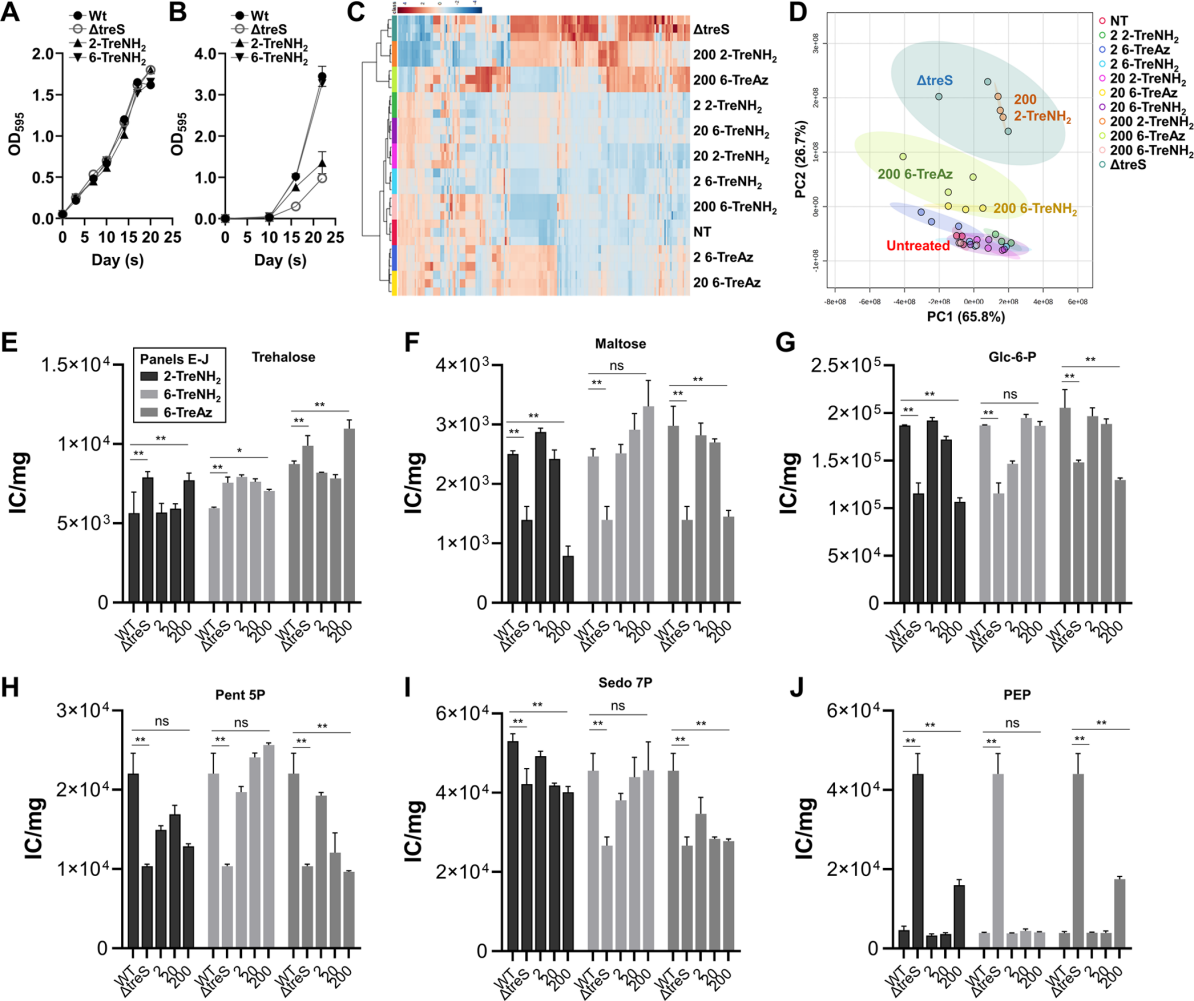
2-TreNH_2_ treatment mimics the metabolic impact of *treS* deletion in Mtb. (A, B) Mtb H37Rv wild type was cultured under (A) planktonic or (B) biofilm growth conditions in the presence of 200 μM of the indicated trehalose analogue and growth was monitored over time. Mtb ΔtreS was included as a control. (C–J) Biofilm metabolomics analysis. Metabolomes from 28-day-old Mtb biofilm persisters after treatment with 0, 2, 20, and 200 μM 2- or 6-TreNH_2_ were collected and analyzed by liquid chromatography-mass spectrometry (LC-MS). ΔtreS and treatment with the same amount of 6-TreAz were used as positive controls. (C, D) Biofilm metabolomics pattern analysis by clustered heatmap analysis (C) and PCA (D) plots. See Figure S18 for the chemical impact on Mtb metabolome in a concentration-dependent manner by PCA analysis. (E–J) Intracellular pool sizes of Mtb intermediates of trehalose metabolism (E, F), glycolysis (G, H), and pentose phosphate pathway (I, J) in Mtb wild type and ΔtreS biofilm persisters. All values are the average of independent biological triplicates ± standard error of the mean. **, *P* < 0.05; ns, not significant by Student *t* test.

Our above data demonstrate that 2-TreNH_2_ potently and selectively inhibits *in vitro* biofilm growth in both Msmeg and Mtb in a trehalose metabolism-dependent manner, suggesting that this compound may target Mtb persister formation through inhibition of the trehalose catalytic shift. Therefore, we performed additional mechanism-of-action studies in Mtb to determine whether 2-TreNH_2_ exerts its activity through inhibition of TreS, the trehalose isomerase essential for the trehalose catalytic shift. First, we monitored the kinetics of planktonic growth and biofilm-persister formation in wild-type Mtb treated with 2-TreNH_2_, and the results were compared to Mtb ΔtreS, a TreS-deficient mutant. For comparison as a negative control, we also included 6-TreNH_2_, which exhibited no inhibitory activity in Msmeg or Mtb. Neither treatment with 2- or 6-TreNH_2_ nor *treS* deletion impacted Mtb planktonic growth, as expected (Figure 3A). However, treatment with 2-TreNH_2_, but not the control compound 6-TreNH_2_, selectively reduced biofilm-persister formation in wild-type Mtb to approximately the same level as Mtb ΔtreS (Figure 3B). Thus, 2-TreNH_2_ treatment phenocopies *treS* deletion with respect to Mtb biofilm-persister growth kinetics.

To investigate the mechanistic basis underlying 2-TreNH_2_-mediated biofilm-persister inhibition, we conducted metabolomic profiling of compound-treated wild-type Mtb biofilm, and compared with that of Mtb ΔtreS mutant. We collected the metabolomes from the 28-day-old biofilm-persister bacilli of 2-TreNH_2_-, 6-TreNH_2_-, or 6-TreAz-treated (or untreated) wild-type Mtb or untreated Mtb ΔtreS, and quantified ∼250 known metabolites. Clustered heatmap analysis and principal component analysis (PCA) plots displayed a clear metabolic similarity between Mtb treated with 200 μM 2-TreNH_2_ and ΔtreS, which was segregated from that of Mtb treated with 200 μM 6-TreNH_2_ and untreated controls (Figure 3C,D; Figure S18). We found that the metabolic patterns of Mtb treated with increasing doses of 2-TreNH_2_ gradually migrated toward that of ΔtreS in PCA results, indicating that Mtb metabolic damage due to 2-TreNH_2_ was almost identical to that of ΔtreS. Given our prior finding that 6-TreAz inhibits the trehalose catalytic shift, we included this compound in our analysis as an additional control. Although Mtb metabolic patterns after 200 μM 6-TreAz treatment were quite similar to those of ΔtreS, the impact of 6-TreAz was slightly different than 2-TreNH_2_, with the latter displaying the closest similarity to ΔtreS. Metabolic networks of Mtb after treatment with varying doses of 6-TreNH_2_ showed no significant changes as compared to that of untreated controls. Using the metabolites that were altered in each condition, we conducted pathway enrichment analysis (Table S1). As expected, a substantial number of pathways were damaged commonly in Mtb after chemical treatment with 2-TreNH_2_ and *treS* genetic deficiency in ΔtreS under a biofilm-persister state. Intriguingly, 2-TreNH_2_-treated Mtb showed additional pathways that were remodeled, including pathways involved in arginine and proline metabolism, cysteine and methionine metabolism, carbapenem biosynthesis, and pentose and glucuronate interconversion. This suggested that 2-TreNH_2_ may have additional targets. We also observed that Mtb in a biofilm-persister state remodeled several metabolic pathways after treatment with 6-TreNH_2_ but still was able to form biofilm-persister bacilli at levels similar to untreated controls, suggesting that the affected metabolic pathways were not essential for persister formation (Table S1).

Next, targeted metabolomic analysis was conducted to monitor the abundance of trehalose metabolism and CCM intermediates. Similar to that of the ΔtreS biofilm-persister metabolome, 2-TreNH_2_-treated Mtb accumulated trehalose with reciprocal depletion of maltose, resulting in continued deficiency in carbon flux toward the biosynthesis of intermediates in upper glycolysis (e.g., glucose 6P) and pentose phosphate pathway (PPP) (e.g., pentose 5P and sedoheptulose 7P) (Figure 3E–I). As expected, the metabolic patterns caused by treatment with 2-TreNH_2_ or *treS* genetic deficiency were not observed in Mtb after treatment with 6-TreNH_2_. Separately, we previously reported that substantial depletion of the lowest intermediate in glycolysis, phosphoenolpyruvate (PEP), also played an important role in provoking Mtb drug tolerance and persister formation.^40^ Intriguingly, ΔtreS failed to downregulate PEP abundance under a biofilm-persister state, which was also observed in Mtb after treatment with 2-TreNH_2_ but not 6-TreNH_2_ (Figure 3J). Overall, our metabolomic analysis demonstrated that the impaired Mtb biofilm-persister formation observed after treatment with 2-TreNH_2_ was largely attributed to a defective TreS-mediated trehalose catalytic shift.

To confirm that 2-TreNH_2_ inhibits TreS-catalyzed isomerization of trehalose to maltose, we used our previously established *in vitro* TreS enzyme assay.^10^ We monitored the extent of the inhibitory effect of 2- or 6-TreNH_2_ compounds against TreS activity. As expected, 2-TreNH_2_ showed 38–55% suppression of the maltose production activity of TreS at 100 μM, comparable to that of our previously reported TreS inhibitor 6-TreAz (Figure S19). Intriguingly, despite its lack of activity in Msmeg and Mtb cells, 6-TreNH_2_ also showed a weaker inhibitory effect, ranging from 10 to 50% on TreS activity. In addition, Mtb TreS did not convert 6-TreAz or 2-TreNH_2_ to the corresponding maltose analogues, 6-azido or 2-amino maltose, indicating that neither compound served as a substrate of TreS-mediated trehalose isomerization. This result is consistent with a published report showing that Msmeg TreS does not isomerize 6-TreAz.^41^ Combined, our data suggest that 2-TreNH_2_ is the best TreS-specific inhibitor among the compounds tested and harbors the most promising activity on Mtb biofilm persisters.

### TreAz and TreNH_2_ Analogues Sensitize *Mycobacterium tuberculosis* to Existing Antitubercular Compounds and Are Active in a Macrophage Infection Model

**Figure 4:**
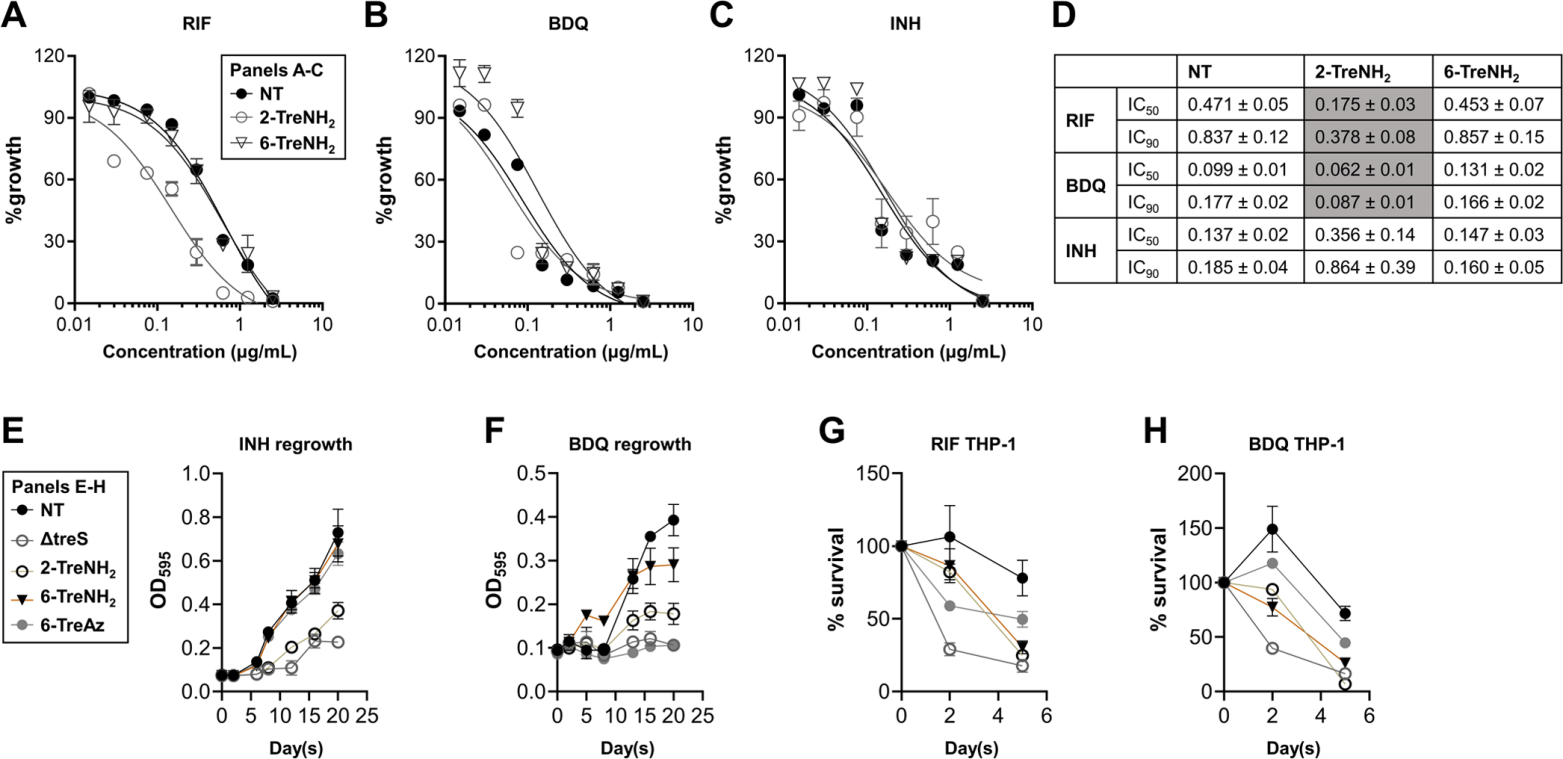
TreAz and TreNH_2_ analogues potentiate the activity of existing anti-TB drugs. (A–C) Dose–response growth of Mtb for (A) RIF, (B) BDQ, and (C) INH were measured with cotreatment with 200 μM trehalose analogues. The OD growth was monitored at 10 days of incubation and calculated by % growth relative to NT with no antibiotic. (D) Half-maximal inhibitory concentration (IC_50_) and 90% inhibitory concentration (IC_90_) shift resulting from treatments in (A–C) were determined by Prism software (Ver 9.5). (E, F) Effect of 200 μM trehalose analogues on Mtb outgrowth following treatment with 30× MIC of (E) INH and (F) BDQ for 5 days. (G, H) Effect of 200 μM trehalose analogues on colony-forming units (CFU)-based viability of Mtb within THP-1 macrophages following treatment with 10× MIC of (G) RIF and (H) BDQ. All values are the average of biological triplicates ± standard error of the mean.

The trehalose catalytic shift is a metabolic strategy used by Mtb to survive environmental pressure, including antibiotic treatment.^10^ Thus, we tested whether cotreatment with trehalosamine compounds increases the antimicrobial effects of known anti-TB antibiotics such as rifampicin (RIF), isoniazid (INH), and BDQ against Mtb growing planktonically. Consistent with the previous literature^10^ and our results monitoring biofilm-persister formation, 2-TreNH_2_, but not 6-TreNH_2_, significantly enhanced the antimicrobial effects of RIF and BDQ (Figure 4A,B). The IC_90_ values of RIF and BDQ on wild-type Mtb were 0.84 ± 0.12 and 0.18 ± 0.02 μg/mL, which were improved to 0.38 ± 0.08 and 0.09 ± 0.01 μg/mL, respectively, as a result of cotreatment with 2-TreNH_2_ (Figure 4D). The improved IC_90_ values represent a potentiation effect because Mtb planktonic growth was unaltered by treatment with 2-TreNH_2_ alone at the same concentration (Figure 3A). Interestingly, antimicrobial potentiation was not observed in cotreatment experiments with INH, presumably due to its strong and rapid bactericidal effects,^42,43^ and in fact led to an unanticipated MIC increase (Figure 4C,D). Thus, we measured the viable but nonculturable colonies after cotreatment of 2-TreNH_2_ and INH or BDQ by monitoring the outgrowth kinetics in antibiotic-free 7H9 medium after inoculating with each culture.^44^ Mtb cultures treated with INH or BDQ exited the lag phase at day 7, but ΔtreS and Mtb after cotreatment with 2-TreNH_2_ showed a prolonged period of lag phase without evidence resuming the growth until day 20 (Figure 4E,F). On the other hand, Mtb cultured with cotreatment with INH and the control compound 6-TreNH_2_ showed regrowth kinetics similar to Mtb culture without any trehalose analogue. The results from Mtb outgrowth experiments suggest that 2-TreNH_2_ also affects viable but nonculturable bacilli putatively formed after treatment with INH or BDQ.

We next tested whether the antimicrobial potentiation resulting from cotreatment with conventional TB antibiotics and trehalose analogues during *in vitro* growth was also effective against intracellular Mtb bacilli. We used THP-1 human macrophages as a host and treated Mtb-infected THP-1 cells with 1X minimum inhibitory concentration (MIC) of RIF or BDQ in the presence or absence of trehalose analogue. RIF and BDQ showed a weak bactericidal effect on intracellular Mtb when administered alone. However, cotreatment with 2-TreNH_2_ enhanced the activity of RIF from ∼10% to greater than 80%, nearly identical to the effect of *treS* deletion (Figure 4G). Similar drug potentiation against intracellular Mtb was observed during cotreatment with BDQ and 2-TreNH_2_ (Figure 4H). In both the RIF and BDQ cotreatment experiments, the effect of 2-TreNH_2_ was significantly better than that of 6-TreAz. Surprisingly, 6-TreNH_2_, which showed no impact on *in vitro* growth or antimycobacterial potentiation, showed a potentiation effect against intracellular Mtb at levels similar to that of 2-TreNH_2_ (Figure 4G,H). It is possible that 6-TreNH_2_ has Mtb bactericidal targets only under intracellular conditions or host cell-specific targets to provoke the observed effects. Finally, none of the trehalose analogues shown to have inhibitory activity against Mtb in liquid culture or macrophages were cytotoxic to mammalian U-937 cells at concentrations up to 1 mM (Figure S20), underscoring their potential as safe and selective inhibitors of Mtb persister formation within a host environment.

### Cryo-Electron Microscopy Reveals That 6-TreAz Binds to the Active Site of Mtb TreS and Induces a Conformational Change

Our results demonstrate that small-molecule inhibitors of TreS sensitize Mtb to existing TB antibiotics and may have value as adjunctive therapeutics. To understand the mechanisms of TreS catalysis and inhibition, and to gain insight into specific interactions of inhibitors with TreS active site residues, we sought to determine the structure of TreS in complex with the inhibitors described herein. To date, no structures of TreS bound to native substrates or substrate analogues have been reported. We initially attempted cocrystallization of wild-type Mtb TreS with substrates or inhibitors. However, consistent with a previous report,^45^ our solved crystal structures, resolved to a resolution of 2.7 Å, lacked density corresponding to any of the compounds within the enzyme active site. Given that TreS preferentially crystallizes in the unliganded form, we turned to cryo-EM to elucidate the structures of enzyme–inhibitor complexes. In addition, we aimed to mitigate against the possibility of substrate or substrate analogues being reacted upon by the enzyme. Previously published results using Msmeg TreS unambiguously identified the aspartic acid residue corresponding to the D238 residue of Mtb TreS as the nucleophile that initiates the attack on the anomeric carbon of either trehalose or maltose.^45−47^ Therefore, we constructed a catalytically inactive Mtb TreS-D238A variant, which was used to obtain a cryo-EM structure of Mtb TreS in complex with a trehalose analogue inhibitor.

**Figure 5:**
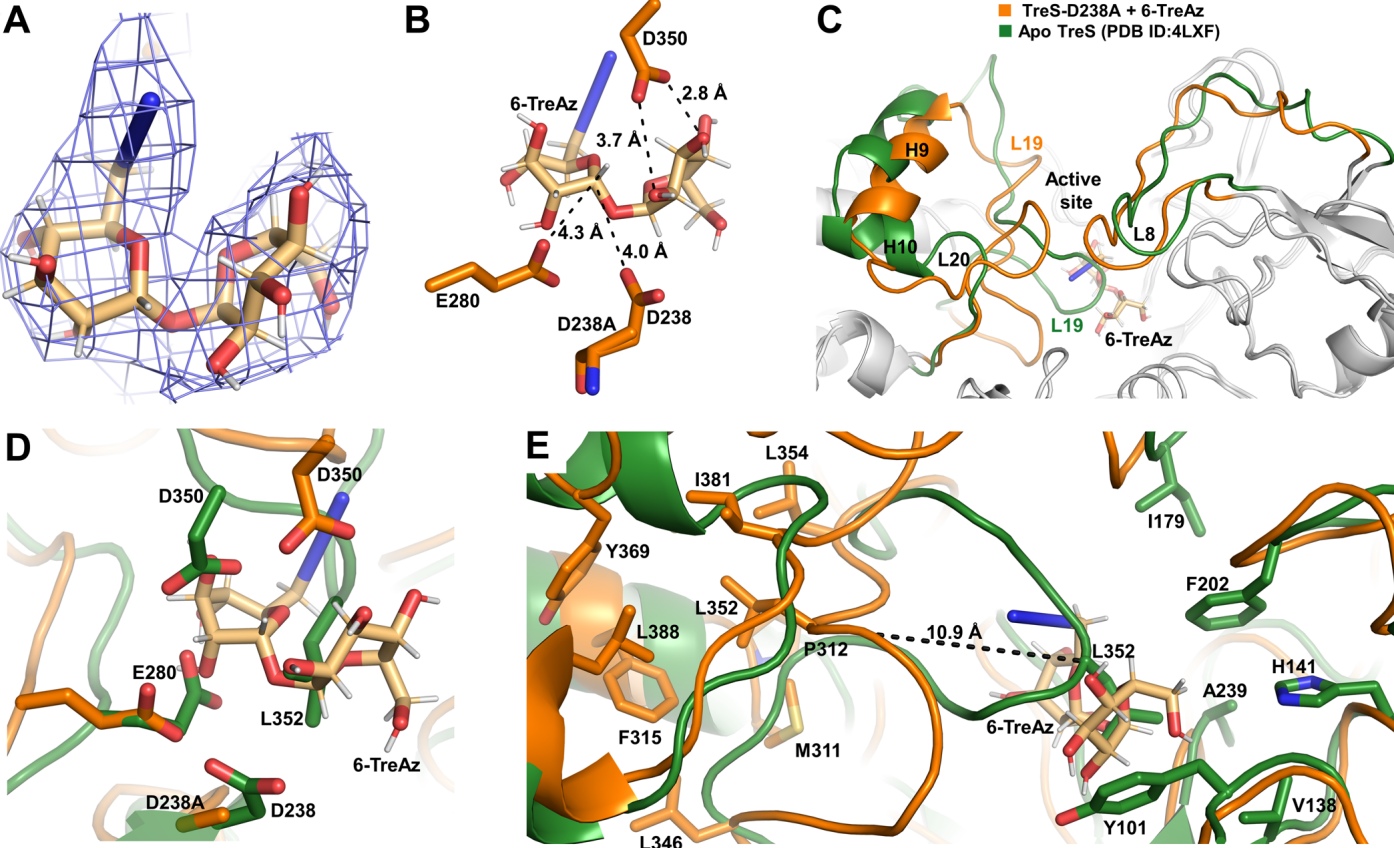
Cryo-EM structure of TreS/6-TreAz complex reveals a ligand-induced conformational change in TreS. (A) Density map for the 6-TreAz ligand. (B) Interactions formed between the ligand 6-TreAz and the catalytic amino acid residues at the active site of TreS-D238A. The D238A is shown as D238 and aligned to illustrate the likely interactions in wild-type TreS. (C) Conformational change observed from the alignment of the 6-TreAz-bound TreS-D238A to the published apo structure of Mtb TreS (PDB ID: 4LXF). Regions without significant changes in the alignment are colored in gray. (D) Changes of active site residues upon 6-TreAz binding. (E) Structural changes of the L19 loop and the repositioning of L352.

Cryo-EM experiments using TreS-D238A in the presence of 6-TreAz afforded the 3-dimensional reconstruction of the Mtb TreS tetramer/6-TreAz to a resolution of 3.6 Å (PDB ID: 8UQV) (Figure S21). As the tetramer of the Mtb TreS crystal structure (PDB ID: 4LXF) was used as the preliminary model for fitting the initial maps, observed differences between the initial model and maps clearly illustrate a conformational change potentially induced by ligand binding. Following multiple rounds of manual and computational real space refinement, clear density corresponding to a disaccharide molecule was observed near the C-terminal end of the β-sheets of the barrel where the active site of a TIM-barrel fold protein is typically located (Figure 5).^48^ The observed density for 6-TreAz was stronger for the D chain of the tetramer compared to the other 3 chains. Therefore, for analysis purposes, the D chain was used for alignments and for defining the specific interactions within the active site. The 6-azido-glucosyl moiety of 6-TreAz fit within the cryo-EM map is modeled with multiple axial hydroxyls as that is the best fit (Figure 5A). This higher-energy conformation may derive from steric hindrance between the C6 azido group and the enzyme active site residues, or it may reflect a conformational change in the substrate required to promote catalysis. As other glycosyl hydrolases generally bind glucosyl moieties and react upon them in the low-energy chair conformation,^49,50^ it is reasonable to assume that the high-energy conformation of that portion of 6-TreAz is due to the addition of the azido moiety and is the direct cause of TreS inhibitory activity.

Based on published literature and the determined structures, the three conserved catalytic residues of TreS are D238 (nucleophile), E280 (general acid/base), and D350 (substrate coordination).^45−47^ The side chain of the E280 residue is positioned 4.3 Å from the C1 hydrogen of the pyranose possessing the azido group containing the azido group (Figure 5B). When the D238A residue is modeled as aspartate to represent the wild-type form of the enzyme, this places the side chain 4 Å closer to the C1 carbon of the azide-containing pyranose moiety and able to undergo a nucleophilic attack on the true substrate.

To understand the conformational changes that occur upon complex formation, the 6-TreAz-bound Mtb-TreS-D238A cryo-EM structure was aligned with the crystal structure of wild-type TreS protein incubated with 6-TreAz, treating that structure as a control since 6-TreAz is not observed in that crystal structure (PDB ID: 8UZH). Additionally, we determined the X-ray crystal structure of the ligand-free TreS-D238A variant. This structure is consistent with wild-type Mtb TreS, which indicates that the D238A mutation alone was insufficient to produce the observed structural changes in the cryo-EM structure of the TreS-D238A/6-TreAz complex. The 6-TreAz-bound complex and the ligand-free wild-type Mtb TreS structures were also superimposed with the published wild-type ligand-free TreS crystal structure (PDB ID: 4LXF). The structural alignment of the published ligand-free TreS structure with the solved crystal structure of wild-type TreS protein incubated with 6-TreAz shows no structural differences (Figure S22). However, as shown in Figure 5C, the 6-TreAz-bound cryo-EM structure shows significant movement of the L19 and L20 loops at the active site along with the H9 and H10 helices and L8 loop vacating the space required to accommodate 6-TreAz binding. The structural alignment of the ligand-free and 6-TreAz complex also highlights the movement of the two catalytic residues of TreS upon binding 6-TreAz (Figure 5D). The position of the D238A nucleophile is little changed. However, the E280 residue, which acts as the general acid, moves slightly away from the ligand binding site to better accommodate the ligand and afford the acid/base chemistry essential for the enzymatic reaction. Specifically, E280 donates a proton to the C1 hydroxyl of the leaving group following nucleophilic attack by D238 and, following glycone rotation, subsequent proton abstraction from the C4 hydroxyl that functions as the nucleophile during the formation of the new α-1,4-glycosidic bond to produce maltose. Additionally, D350, which is essential for coordinating ligand binding, moves toward the substrate and forms a hydrogen-bonded interaction with each of the two rings of 6-TreAz. Like other enzymes catalyzing similar reactions on sugars, D350 may promote nucleophilic attack on the anomeric carbon by slightly modulating the electronics within the rings through hydrogen-bonded interactions with the C2 hydroxyl group. This is analogous to the role of D503 in the Mtb GlgE enzyme, D480 in the *Streptomyces coelicolor* GlgE1 enzyme that catalyzes the formation of 1,4-glycosidic bond in growing α-glucans, D295 of *Leuconostoc mesenteroides* sucrose phosphorylase that catalyzes sucrose phosphorolysis to produce α-d-glucose 1-phosphate and d-fructose, and other glycosyl hydrolase family 13 members.^51−53^ In each of the aforementioned systems, the conserved aspartate residue forms a bidentate interaction with the hydroxyls at positions 2 and 3 of the respective rings.

Other structural differences in the ligand-free structure highlight the significant reorientation of active site loops, both to accommodate bound ligand and to prevent loss of the glucose intermediate during the required glycone rotation. The side chain of L352 harbored by the L19 loop occupies the same volume of space in the ligand-free structure that is occupied by 6-TreAz in the TreS-D238A/6-TreAz complex structure. Specifically, the position of the L352 residue in the ligand-free structure is stabilized by hydrophobic interactions with Y101, V138, H141, I179, F202, and A239 (Figure 5E). However, in the 6-TreAz-bound structure, the L19 loop containing L352 travels 10 Å from the active site with the new conformation stabilized via interactions between the L352 side chain and a different hydrophobic network formed by residues M311, P312, F315, L346, L354, M365, Y369, I381, and L388 (Figure 5E). Additionally, the H9 helix between the L19 and L20 loops and the L8 loop slightly shift toward the active site. This reorientation likely locks the compound at the active site and facilitates the catalytic reaction through preventing release of the glucose intermediate and promoting rotation and formation of the new 1,4-glycosidic linkage. Finally, to further highlight the unique active site structure in TreS stimulated by ligand binding, the structure was superimposed onto the TreS portion of the *M. smegmatis* TreS-Pep2 complex (PDB ID: 5JY7) to determine if the complex with Pep2 promotes a similar conformational change in TreS.^54^ Once again, the conformational differences in the 6-TreAz complex were clearly observed as TreS in TreS-Pep2 complex possesses the same conformation as the apo structure, which further strengthens the hypothesis that only substrates or substrate analogues stimulate this structural change.

From a drug development perspective, it will be of interest to quantify the thermodynamic differences between the two TreS conformations to ascertain the need of stimulating this conformational change with novel TreS inhibitors, or if the ligand-free form is a druggable target. It may be possible to develop an allosteric inhibitor of TreS by targeting the ligand-free form, thereby preventing the conformational change required for substrate binding and catalysis. For example, targeting the hydrophobic pocket that accommodates L352 in the 6-TreAz-bound form of TreS could block the conformational shift necessary to promote catalysis.

## Conclusions

The discovery, characterization, and targeting of Mtb persistence factors is emerging as an attractive pathway toward adjunctive therapeutics that could be used alongside existing drugs to increase the speed and efficacy of TB treatment. The trehalose catalytic shift, which is required for Mtb persister formation in a biofilm model, was recently identified as a promising source of targets for persister-focused inhibitor development. Specifically, TreS-catalyzed isomerization of recycled trehalose into maltose is a key step in the trehalose catalytic shift that promotes Mtb survival during stress. Here, we took a multidisciplinary approach spanning synthetic chemistry, microbiology, metabolomics, and structural biology to develop substrate analogue inhibitors of TreS that selectively block Mtb biofilm-persister formation and potentiate TB drugs against Mtb growing in liquid medium or inside macrophages. Chemoenzymatic synthesis was instrumental in rapidly generating a panel of systematically altered TreAz and TreNH_2_ analogues, which enabled initial SAR assessment on the focused 8-compound panel and identification of 6-TreAz and 2-TreNH_2_ as lead compounds. Quantitative mass spectrometry-based metabolomic analysis of inhibitor-treated Mtb biofilm-persister cells identified TreS as a target of both compounds and showed that 2-TreNH_2_ treatment most accurately mimics the metabolic damage of *treS* deletion. 2-TreNH_2_ is a natural product whose antimycobacterial activity has been known for decades, but whose mechanism of action, TB drug potentiation, and lack of toxicity against mammalian cells were previously unknown and revealed here for the first time. Interestingly, in combination treatment experiments, different TB antibiotics were variably potentiated by 2-TreNH_2_ during in vitro growth, and INH potency unexpectedly decreased. Although the precise reason for this variability is presently unknown, we nonetheless found in outgrowth kinetics and macrophage infection experiments that 2-TreNH_2_ strongly potentiated multiple TB antibiotics, including INH in outgrowth experiments, nearly to the same level as deletion of treS. This is consistent with our prior work showing that Mtb engages the trehalose catalytic shift as an adaptive strategy to mitigate antibiotic treatment-induced reactive oxygen species (ROS) production, activate an alternate ATP biosynthetic pathway, and biosynthesize antioxidant chemicals, all of which are advantageous to survive antibiotic effects regardless of their modes-of-action.^10^ An important next step will be to investigate whether TreS inhibitors improve the efficacy of TB treatment in animal models of Mtb infection, which will require significantly larger compound quantities and may necessitate innovations to scale up the synthetic methods. We will also investigate the impact of TreS inhibitors on the frequency of the emergence of drug-resistant mutants through inhibition of persister formation. Given the differential activities of the trehalose analogues tested, extension of this research to related analogues (e.g., epimers, nonhydrolyzable analogues) to generate richer SAR data, as well as testing in mycobacterial pathogens besides Mtb, is warranted. Finally, this study reported the first three-dimensional (3D) structure of TreS in complex with a substrate or substrate analogue. Critically, because TreS crystallization is preferential for the unliganded form and wild-type enzyme presents potential issues with respect to analogue reactivity, we used cryo-EM to obtain the structure of a catalytically inactive TreS mutant (D238A) in complex with 6-TreAz. The TreS-D238A/6-TreAz structure showed that 6-TreAz binds to the enzyme active site, inducing a conformational change that is likely necessary for catalysis and could be exploited to develop new strategies for inhibiting TreS. These results also highlight the utility of cryo-EM as a key technique for obtaining high-resolution structures of TreS bound to other substrate analogues, including 2-TreNH_2_. Collectively, our study (i) validates TreS inhibition as a strategy to target Mtb persisters; (ii) identifies selective and cell-active substrate analogue inhibitors of TreS that are lead compounds for adjunctive therapeutic development; and (iii) provides TreS structural and mechanistic knowledge that can be leveraged in future TB drug development efforts. More broadly, our study serves as a model multidisciplinary approach to identifying inhibitors that target persistent infections, which are caused by diverse bacterial pathogens.

## Materials and Methods

### General Procedures for Compound Synthesis

Reagents and solvents were procured from commercial sources without further purification, unless otherwise noted. TreT enzyme was expressed and purified as previously reported Meints et al.^1^ Monosaccharides were obtained from Sigma (6-GlcAz), Synthose (3-GlcAz), Carbosynth (UDP-GlcNAc), and Abcam (UDP-glucose). Thin-layer chromatography (TLC) analysis was performed on glass-backed silica 60 Å plates (thickness 250 μm) from Silicycle and visualized either by charring with 5% H_2_SO_4_ in ethanol or gentle warming with ninhydrin stain. Column chromatography was performed on flash-grade silica gel 32–63 μm (230–400 mesh) from Silicycle. ^1^H and ^13^C NMR spectra were recorded at 500 and 126 MHz, respectively, on a Bruker Avance 500 NMR spectrometer.

### Synthesis of 2-TreAz and 2-TreNH_2_

2-TreAz and 2-TreNH_2_ were synthesized using a combination of chemoenzymatic synthesis using the approach of Groenevelt et al.^22^ and chemical synthesis using the approach of Swarts et al.^29^^1^H and ^13^C NMR data (Supporting Information) matched the literature data.

### Synthesis of 4-TreAz and 2-TreNH_2_

4-TreAz and 4-TreNH_2_ were chemically synthesized using the approach of Bassily et al.^37^^1^H and ^13^C NMR data (Supporting Information) matched the literature data.

### Synthesis of 3-TreAz and 6-TreAz

The syntheses of 3-TreAz and 6-TreAz were carried out using a reported chemoenzymatic synthesis method.^35,36^ His-tagged TreT enzyme was expressed and purified from *Escherichia coli* as reported.^36^ To a 15 mL conical tube were sequentially added 3-azido-3-deoxy-d-glucose or 6-azido-6-deoxy-d-glucose (16.4 mg, 20 mM final concentration), UDP-glucose (97.6 mg, 40 mM final concentration), MgCl_2_·6H_2_O (16.2 mg, 20 mM final concentration), and 4 mL of His-tagged TreT enzyme (300 μg/mL) in Tris buffer. The contents of tube were mixed thoroughly by pipetting and the reaction was incubated at 70 °C with shaking for 60 min, after which the reaction mixture was cooled on ice for 20 min. The reaction mixture was added to a pre-rinsed Amicon Ultra-15 centrifugal filter unit and centrifuged at 3900*g* for 20 min. The upper chamber of the filter unit was washed twice with water (3 mL) and centrifuged at 3900*g* for 20 min. After discarding the upper chamber of the filter unit, mixed-bed ion-exchange resin (Bio-Rad Bio-Rex RG 501-X8, 3 g) was added and the mixture was stirred for 1 h. The supernatant was collected and the resin was washed twice with water (5 mL). The supernatants were combined and dried via rotary evaporation to give 3-TreAz (26.8 mg, 92% yield, white solid) or 6-TreAz (26.7 mg, 92% yield, white solid). ^1^H and ^13^C NMR data (Supporting Information) matched the literature data from Swarts et al.^29^

### Synthesis of 3-TreNH_2_ and 6-TreNH_2_ Analogues

TreAz analogues were reduced to the corresponding TreNH_2_ analogues via Pd-catalyzed hydrogenation using the procedure of Rodriguez-Rivera et al.^55^ To an argon-flushed round-bottom flask containing a stirring solution of TreAz analogue (6-TreAz: 28.2 mg, 0.076 mmol; 3-TreAz: 18.3 mg, 0.049 mmol) in water (6-TreAz: 5 mL; 3-TreAz: 5 mL) was added 10% wt. Pd/C (6-TreAz: 14 mg; 3-TreAz: 9 mg). A hydrogen-filled balloon was attached to the flask to replace the argon atmosphere. The reaction was stirred at room temperature overnight, after which the catalyst was filtered and the filtrate was dried via rotary evaporation to give the corresponding TreNH_2_ analogue (3-TreNH_2_: 16.7 mg, 98% yield, white solid); for 6-TreNH_2_, the sample was treated with glacial acetic acid (200 μL), filtered, and dried via rotary evaporation to give the acetate form (29.1 mg, 95% yield, white solid). ^1^H and ^13^C NMR data (Supporting Information) matched the literature data from Lu et al.^23^

### Bacterial Strains and Culture Conditions

Msmeg WT, ΔsugC mutant^38^ (hygromycin B, 50 μg/mL), or sugC::sugC complement^19^ (hygromycin B, 50 μg/mL; apramycin, 10 μg/mL) was cultured at 37 °C in M63 medium (M63 salts minimal medium supplemented with 2% glucose, 0.5% casamino acids, 1 mM MgSO_4_, and 0.7 mM CaCl_2_) with or without Tween-80 as detergent and antibiotic if noted. Mtb H37Rv WT, ΔsugC mutant (hygromycin B, 50 μg/mL),^38^ ΔsugC::sugC complement (hygromycin B and kanamycin, 50 μg/mL each),^38^ and ΔtreS mutant (hygromycin B, 50 μg/mL)^10^ were cultured at 37 °C in Sauton’s medium^56^ (KH_2_PO_4_ 0.5 g/L, MgSO_4_·7H_2_O 0.5 g/L, citric acid 2 g/L, ferric ammonium citrate 0.05 g/L, 1% ZnSO_4_ solution 0.1 mL/L, l-asparagine 4 g/L, glycerol 6% v/v) supplemented with or without 0.4% tyloxapol as detergent and antibiotic if noted. Outgrowth experiments were conducted in Middlebrook 7H9 medium (2.5 g/L disodium phosphate, 1.0 g/L monopotassium phosphate, 0.5 g/L monosodium glutamate, 0.5 g/L ammonium sulfate, 0.1 g/L sodium citrate, 0.05 g/L magnesium sulfate, 0.04 g/L ferric ammonium citrate, 1.0 mg/L copper sulfate, 1.0 mg/L zinc sulfate, 1.0 mg/L pyridoxine HCl, 0.5 mg/L biotin, 0.5 mg/L calcium chloride) supplemented with 0.2% glycerol, 0.2% dextrose, 0.04% tyloxapol, 0.5 g/L bovine serum albumin, and 0.085% NaCl. All Mtb H37Rv strains were cultured in a biosafety level 3 facility. Mtb mc^2^7000 auxotroph^9^ was cultured at 37 °C in Sauton’s medium supplemented with 100 μg/mL d-pantothenate and with or without 0.4% tyloxapol as detergent.

### Planktonic and Biofilm Growth Assays

Msmeg planktonic and biofilm growth assays were performed as previously reported by Wolber et al.^19^ For Mtb planktonic and biofilm growth assays, starter cultures of Mtb were grown in Sauton’s medium containing 0.04% tyloxapol detergent and antibiotic (if appropriate) until reaching exponential phase (OD_600_ ∼ 0.8). To initiate planktonic growth assays, starter cultures were diluted to OD_600_ 0.01 in Sauton’s medium containing 0.04% tyloxapol. 100 μL of the diluted culture was added to a sterile polystyrene 96-well plate containing 100 μL of 2-fold serially diluted trehalose analogues in Sauton’s medium to give a final compound concentration of 0–1000 μM, a final volume of 200 μL, and a final OD_600_ of 0.005. Liquid medium with no compound was used as a no treatment (NT) control and 5× MIC INH (5 μg/mL) was used as a positive control. The empty wells in the plate were filled with sterile water to help minimize evaporation. The plates were sealed with sterile breathable film and placed in a secondary container and incubated at 37 °C with shaking for 5 weeks. At regular intervals, the contents of plates were mixed by pipetting up and down and OD_600_ was recorded using a Tecan Infinite M200 Pro microplate reader. At the end of the 5-week incubation period, biofilm growth assays were performed essentially as described by Lee et al.^10^ To initiate biofilm growth assays, starter cultures prepared as described above were diluted to OD_600_ 0.01 in Sauton’s medium without tyloxapol. 100 μL of the diluted culture was added to a sterile polystyrene 96-well plate containing 100 μL of 2-fold serially diluted trehalose analogues in Sauton’s medium to give a final compound concentration of 0–1000 μM, a final volume of 200 μL, and a final OD_600_ of 0.005. Liquid medium with no compound was used as a no treatment (NT) control and INH (5 μg/mL) was used as a positive control. The empty wells in the plate were filled with sterile water to help minimize evaporation. Lids were placed on the plates, which were then triple-sealed with parafilm, placed in a secondary container, and incubated at 37 °C without shaking for 5 weeks. The biofilms were washed three times with phosphate-buffered saline (PBS) and stained using 1% aqueous CV solution. Stained biofilms were washed three times with PBS to remove excess unbound CV, then the biofilm-associated CV was extracted using 95% ethanol. OD_595_ of 20-fold ethanol-diluted CV extract was recorded using a Tecan Infinite M200 Pro microplate reader.

### Metabolite Extraction and LC-MS Metabolomics

For LC-MS metabolomics, at 28-day-old biofilm after media removal, biofilm culture was washed with ice-cold PBS three times and quenched by adding 1 mL of acetonitrile/methanol/H_2_O (40:40:20) precooled to −40 °C. Biofilm metabolites were extracted by mechanical lysis with 0.1 mm zirconia beads in Precellys tissue homogenizer for 6 min (6000 rpm) twice under continuous cooling at or below 2 °C.^10^ Lysates were clarified by centrifugation and then filtered through a 0.22-μm spin-X column. The residual protein content of metabolite extracts was determined to normalize the samples to cell biomass. LC-MS differentiation and detection of extracted metabolites were performed using an Agilent Accurate Mass 6230 TOF coupled with an Agilent 1290 Liquid Chromatography system. The metabolites were separated on a Cogent Diamond Hydride Type C column (gradient 3) (Microsolve Technologies) with solvents and configuration as reported previously.^57^ The mobile phase consisted of solution A (dd-H_2_O with 0.2% formic acid) and solution B (acetonitrile with 0.2% formic acid). An isocratic pump was used for continuous infusion of a reference mass solution to allow mass axis calibration. Detected ions were deemed to be metabolites on the basis of unique accurate mass-retention time identifiers for masses exhibiting the expected distribution of accompanying isotopologues. The abundance of extracted metabolites was analyzed using Profinder B06.00 software and Agilent Qualitative Analysis B.07.00 with a mass tolerance of <0.005 Da. The clustered heatmap, pathway enrichment assay, principal component analysis, and fold change analysis were performed using bioinformatics tools available in MetaboAnalyst v.5.0 (www.metaboanalyst.ca).

### Expression and Purification of Mtb TreS and In Vitro TreS Assay

The *treS* gene (Rv0126) was amplified by PCR using the TreS-specific primers (forward, 5′- CAT ATG AAC GAG GCA GAA CAC AGC GTC – 3′; reverse, 5′ – AAG CTT TCA TAG GCG CCG CTC TCC C −3′) as previously reported.^10^ The amplified gene and pET28a were double digested with NdeI and *Hin*dIII and ligated to construct pET28a::TreS. After the sequence was confirmed, *E. coli* BL21 (DE3) pLysS was used as the expression strain. The *E. coli* BL21 (DE3) pLysS harboring pET28a::TreS was grown at 37 °C in LB medium containing 50 μg/mL of kanamycin and 25 μg/mL of chloramphenicol to an OD_595_ of 0.6–0.7. Expression of the *treS* gene was induced by isopropyl-β-d-thiogalactopyranoside (IPTG) to the cultures to a final concentration of 0.5 mM, and then cells were further grown for 16 h at 16 °C. The cells were harvested from 500 mL cultures and resuspended in 20 mL of lysis buffer (20 mM Tris-HCl [pH 7.5] and 200 mM NaCl) containing protease inhibitor. The resuspended cells were disrupted using sonication, and cell-free crude extracts were obtained by centrifugation at 15,000 rpm for 30 min. The crude extracts were loaded into a column packed with 1 mL of an 80% (v/v) slurry of Ni-Sepharose resin. The resin was washed with 10 bed volumes of lysis buffer containing 5 mM imidazole and washed further with 10 bed volumes of lysis buffer containing 75 mM imidazole. His_6_-tagged TreS was eluted from the resin with 5 bed volumes of lysis buffer containing 250 mM imidazole and 10% glycerol. Imidazole and NaCl were removed from purified TreS by means of a PD-10 desalting column equilibrated with 20 mM Tris-HCl (pH 7.5) containing 10% glycerol.

*In vitro* TreS assays were conducted as previously reported.^10^ TreS activity was measured using a 100 μL *in vitro* enzyme reaction containing 40 mM MOPS (pH 7.0), 10 mM trehalose, and 30 ng purified TreS enzyme in the presence or absence of 100 μM 2-TreNH_2_, 6-TreNH_2_, or 6-TreAz. The reactions without TreS enzyme or trehalose substrate were also included. The TreS enzyme was incubated at 37 °C with the inhibitor for 10 min, after which the reaction was initiated by adding the substrate-buffer mixture. The mixture was incubated at 37 °C for an additional 10 min, then the reaction was quenched by adding ice-cold acetonitrile containing 0.2% formic acid to yield a final 70% acetonitrile mixture. After centrifugation, reaction supernatants were analyzed by LC-MS to quantify maltose (or maltose analogue).

### Antibiotic Potentiation Assays

To monitor the antimycobacterial effects, Mtb cultures were growth-synchronized to late log-phase and back-diluted to an OD_595_ of 0.05 before plating in Middlebrook 7H9 medium. Plates were incubated standing at 37 °C with 5% CO_2_. OD_595_ was evaluated using a plate reader at 10–15 days postplating and percent growth was calculated relative to the untreated control for each. IC_50_ measurements were calculated using a nonlinear fit in GraphPad Prism. For all IC_50_ curves, data represent the mean ± standard error for technical triplicates. Data are representative of at least two independent experiments.

Outgrowth experiments were conducted as previously reported.^44^ Mid log phase Mtb cultures were resuspended in 5 mL of Middlebrook 7H9 medium with 50× MIC-equivalent BDQ or 20× MIC-equivalent INH and incubated at 37 °C for 5 days. The cultures were then diluted 21-fold (10 μL into 200 μL) into fresh 7H9 medium in a new 96-well plate (outgrowth plate). OD_595_ was measured using a plate reader every 2–3 days. All values are the average of biological triplicates ± standard error.

### THP-1 Cell Culture and Intramacrophage Killing Assay

THP-1 cells were grown RPMI-1640 with 10% v/v fetal bovine serum (FBS), 1 mM sodium pyruvate, 2 mM l-glutamine, and PenStrep (100 U/mL penicillin and 100 μg/mL streptomycin) at 37 °C using an incubator with 5% CO_2_. The cells were fed every 3–4 days by removing half of the culture medium and replacing with fresh medium. THP-1 cells were washed with fresh RPMI-1640 without PenStrep medium and adjusted 1 × 10^5^ cells/mL. The cells were treated with 20 nM phorbol myristate acetate (PMA) for 24 h before infection and used to infect a single-cell suspension of Mtb at a mean of infection (MOI) of 10 for 84 h. After 84 h, the cells were extensively washed and the media was replaced with RPMI-1460 with 50 μg/mL gentamycin to remove extracellular bacteria overnight. The cells were and then treated with 1× MIC-equivalent RIF or BDQ in the presence of 100 μM of 2-TreNH_2_, 6-TreNH_2_, or 6-TreAz. To estimate intracellular Mtb growth, infected macrophages were lysed using 0.5% Triton X-100, and serial dilutions were plated on m7H10 and incubated at 37 °C. CFU were determined 21 days later.

### Mammalian Cell Cytotoxicity Assay

Cytotoxicity of 2-TreAz, 2-TreNH_2_, 3-TreNH_2_, 6-TreAz, and 6-TreNH_2_ was assessed in pro-monocytic U-937 cells using commercially available the lactate dehydrogenase (LDH) assay kit (Cayman Chemical). The assay was performed essentially as per kit instructions. Briefly, 2 × 10^5^ U-937 cells/mL in 100 μL RPMI medium with 10% FBS in a flat-bottom 96-well plate in triplicate were treated with 1 mM concentration of 2-TreAz, 2-TreNH_2_, 3-TreNH_2_, 6-TreAz, 6-TreNH_2_, or trehalose. 10% Triton X-100 solution was used as a maximum LDH release control and cell culture grade water was used as the spontaneous LDH release control. The cells were incubated for 48 h at 37 °C with 5% CO_2_, after which the 96-well plate was centrifuged at 400*g* for 5 min and 50 μL of supernatant was transferred to another 96-well plate. To each well, 50 μL of the kit reaction mixture was added, and the plate was incubated at 37 °C with gentle shaking for 30 min in a Tecan Infinite M200 Pro plate reader and absorbance was monitored at 490 nm. From the absorbance readings, percent cytotoxicity was calculated as per kit instructions.

### Expression and Purification of Mtb TreS and Cryo-EM Structural Elucidation

The production of recombinant Mtb wild-type TreS is as previously described but with slight modifications.^45^ The amino acid range from 11 to 587 with a cleavable N-terminal 6× histidine tag was expressed in *E. coli*. A synthetic gene, codon optimized for *E. coli* protein production encoding Mtb TreS-11-587-D238A (IDT-DNA technologies), was amplified by PCR and inserted at the PshA1 cut site of a modified pET32 plasmid (EMD biosciences) and used to transform *E. coli* T7 Express cells (New England BioLabs). A single colony was used to inoculate LB containing 100 μg/mL of carbenicillin and cultured at 37 °C. Gene expression was induced at 16 °C with 1 mM IPTG for 24 h, and the cells were harvested by centrifugation at 4000 rpm for 30 min. Pelleted cells were resuspended in Buffer A containing 50 mM Tris pH 7.5, 500 mM NaCl, 1% (v/v) glycerol, 0.3 mM (tris (2-carboxyethyl) phosphine) (TCEP), and 5 mM imidazole. Final concentrations of 1 μM lysozyme and 0.1 μM DNase I were added to the suspension of cells and the sample was sonicated (Sonicator 3000, Misonix). The lysate was centrifuged at 10,000 rpm for 40 min and the supernatant was loaded onto a His Trap 5 mL column equilibrated with Buffer A. After washing the column with 15 column volumes of Buffer A, bound TreS was eluted with Buffer B containing 150 mM imidazole and the other components of Buffer A. The protein was incubated with rhinovirus 3C protease overnight while dialyzing against buffer A. The tag-cleaved protein was applied to a His Trap 5 mL column equilibrated with Buffer A and the flowthrough was collected. The protein was subjected to size exclusion chromatography to remove any aggregates using 50 mM Tris pH 7.5 buffer, containing 300 mM NaCl and 0.3 mM TCEP.

The sample was diluted to a concentration of 0.5 mg/mL and 3.2 μL of that same sample was blotted to cryo-EM grids (Quantifoil R 1.2/1.3 300 Mesh, Cu) following glow discharge using a PELCO easiGlow glow discharge cleaning system and blotted for 3 s using the Vitrobot Mark IV System (Thermo Fisher). Cryo-EM data were collected at the Iowa State University cryo-EM facility using a Glacios microscope at 200 kV (Thermo Fisher) and a K3 direct electron detector (DED). Data were analyzed and maps were generated using CryoSPARC. A tetramer of the TreS wild-type apo structure available in PBD ID: 4LXF was used as the starting model for the initial fitting within the density map. The structure was further refined using Coot and Phenix. The final model was visualized using Pymol.

## Supporting Information Available

Screen of compound panel at 1 mM in Msmeg (Figure S1); dose responses of compounds in Msmeg (Figures S2–S9); dose responses of compounds in Mtb (Figures S10–S16); LpqY-SugABC dependence of compound activity in Msmeg (Figure S17); supplementary Mtb biofilm metabolomics PCA (Figure S18); evaluation of compounds using in vitro TreS assay (Figure S19); cytotoxicity evaluation of TreAz and TreNH2 inhibitors (Figure S20); 3D reconstruction of the TreS-D238A complex (Figure S21); alignment of available TreS structures (Figure S22); metabolic pathway enrichment (Table S1); and NMR spectra (PDF)
