# Supplementary material for: Targeting Mycobacterium tuberculosis Persistence through Inhibition of the Trehalose Catalytic Shift
Source: ACS Infect Dis. 2024 Mar 14;10(4):1391–404. doi: 10.1021/acsinfecdis.4c00138 (PMC11019547; doi:10.1021/acsinfecdis.4c00138)

Supplementary Information (SI)

for

Targeting *Mycobacterium tuberculosis* persistence through inhibition of the trehalose catalytic shift

Karishma Kalera,[1,2] Rachel Liu,[3] Juhyeon Lim,[3] Rasangi Pathirage,[4] Daniel H. Swanson,[1] Ulysses G. Johnson,[1,2] Alicyn I. Stothard,[1] Jae Jin Lee,[3] Anne W. Poston,[1,‡] Peter J. Woodruff, Donald R. Ronning,[4] Hyungjin Eoh,[3]* and Benjamin M. Swarts[1,2]*

[1]Department of Chemistry and Biochemistry, Central Michigan University, Mount Pleasant, MI, 48859, USA

[2]Biochemistry, Cell, and Molecular Biology Program, Central Michigan University, Mount Pleasant, MI, 48859, USA

[3]Department of Molecular Microbiology and Immunology, Keck School of Medicine, University of Southern California, Los Angeles, CA 90033, USA

[4]Department of Pharmaceutical Sciences, University of Nebraska Medical Center, Omaha, NE 68198, USA

[5]Department of Chemistry, University of Southern Maine, Portland, Maine 04104, USA

*Corresponding author: E-mail: ben.swarts@cmich.edu

*Corresponding author: E-mail: heoh@usc.edu

‡This work is dedicated to A. W. Poston (1970-2022)

SI Table of Contents

Supplementary schemes and figures S3

Figure S1. Screen of compound panel at 1 mM in Msmeg S3

Figures S2-S9. Dose responses of compounds in Msmeg S3-S7

Figures S10-S16. Dose responses of compounds in Mtb S8-S11

Figure S17. LpqY-SugABC dependence of compound activity in Msmeg S12

Figure S18. Supplementary Mtb biofilm metabolomics PCA analysis S13

Figure S19. Evaluation of compounds using in vitro TreS assay S13

Figure S20. Cytotoxicity evaluation of TreAz and $TreNH_2$ inhibitors S14

Figure S21. 3D re-construction of the TreS-D238A complex S15

Figure S22. Aligment of available TreS structures S15

Supplementary tables S16

Table S1. Metabolic pathway enrichment S16

NMR spectra S17

Supplementary schemes and figures

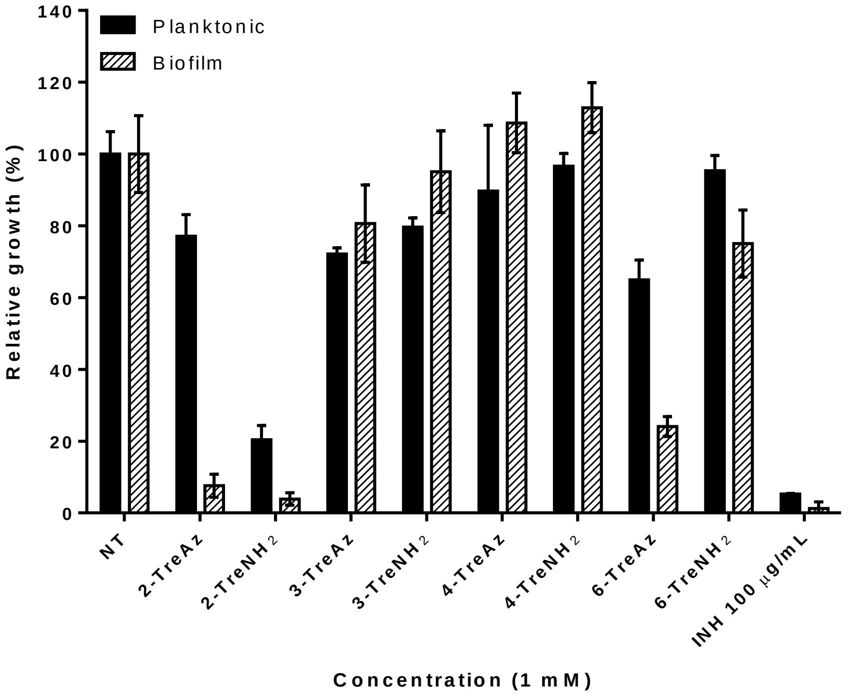

Figure S1. Screen of compound panel at 1 mM against Msmeg planktonic and biofilm growth.

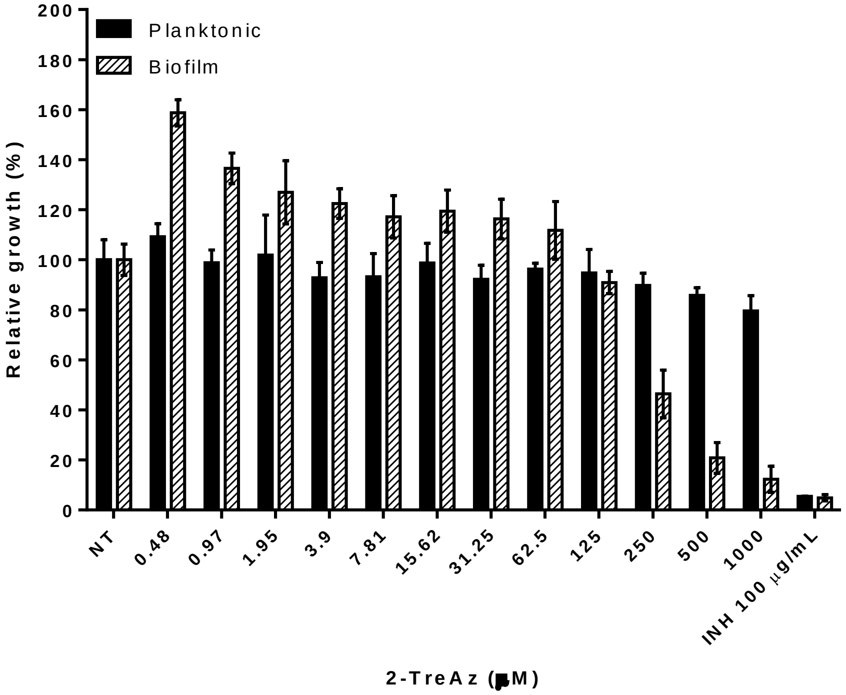

Figure S2. Dose response of 2-TreAz against Msmeg planktonic and biofilm growth.

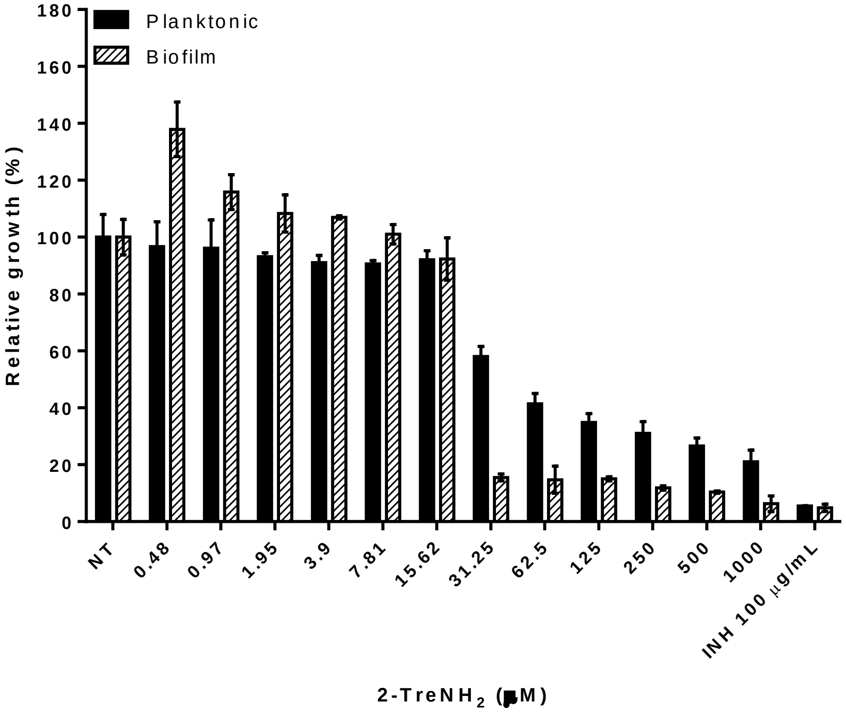

Figure S3. Dose response of 2-TreNH$_2$ against Msmeg planktonic and biofilm growth.

4

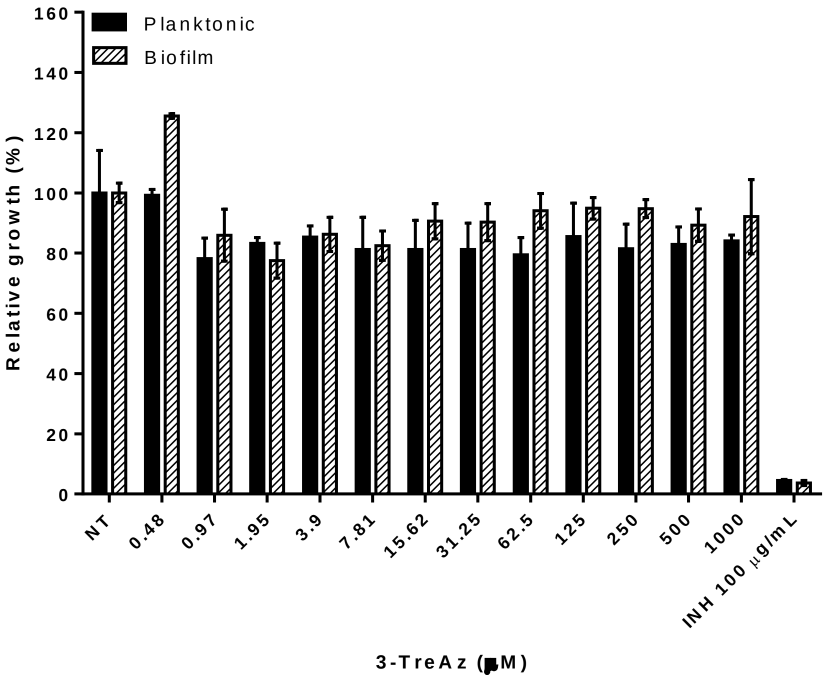

Figure S4. Dose response of 3-TreAz against Msmeg planktonic and biofilm growth.

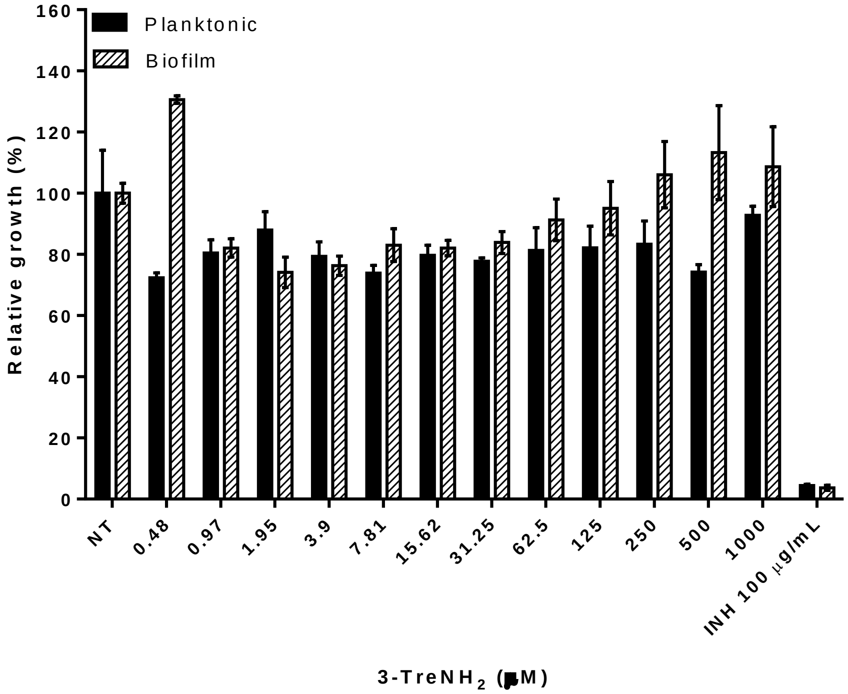

Figure S5. Dose response of 3-TreNH$_2$ against Msmeg planktonic and biofilm growth.

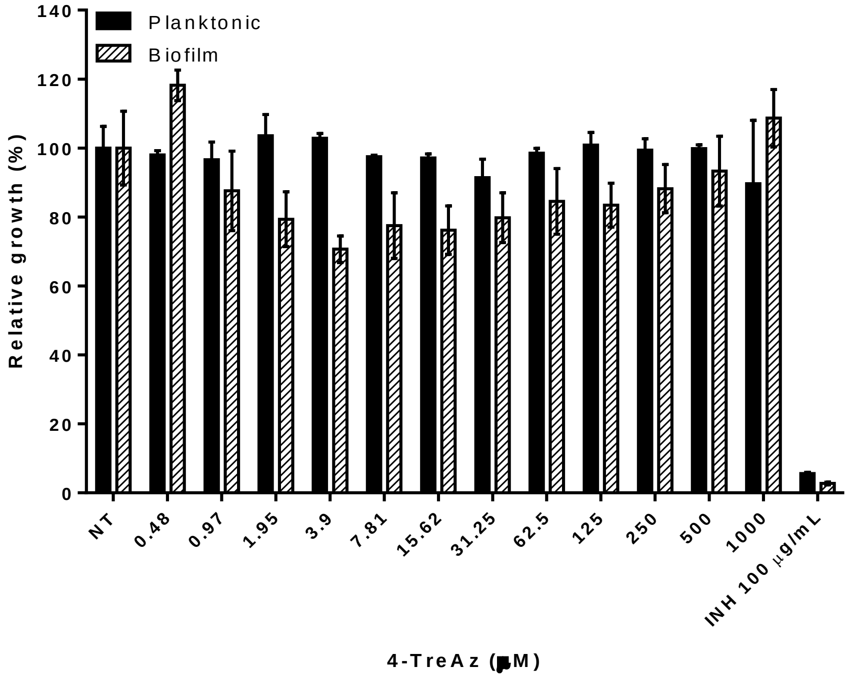

Figure S6. Dose response of 4-TreAz against Msmeg planktonic and biofilm growth.

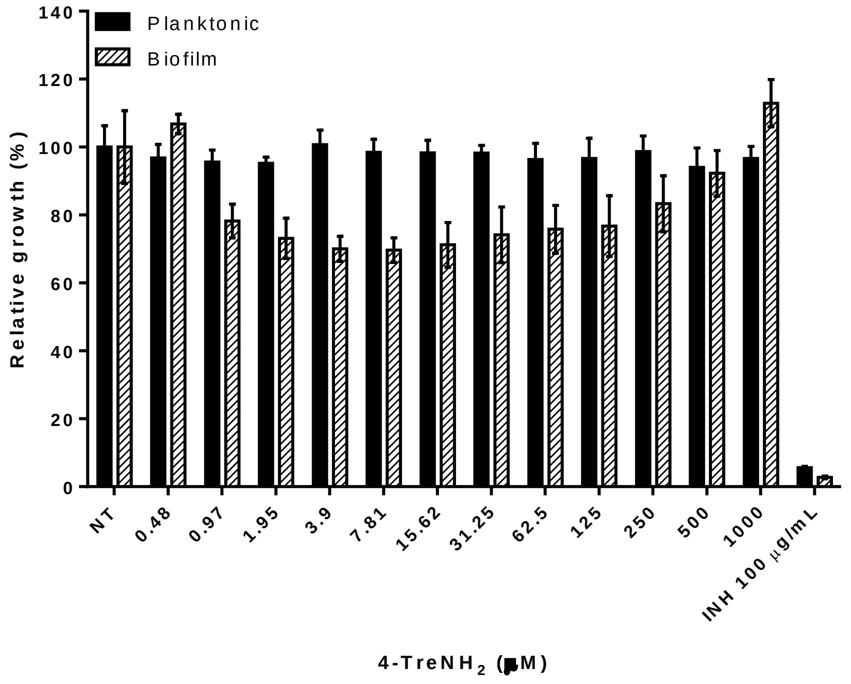

Figure S7. Dose response of 4-$TreNH_2$ against Msmeg planktonic and biofilm growth.

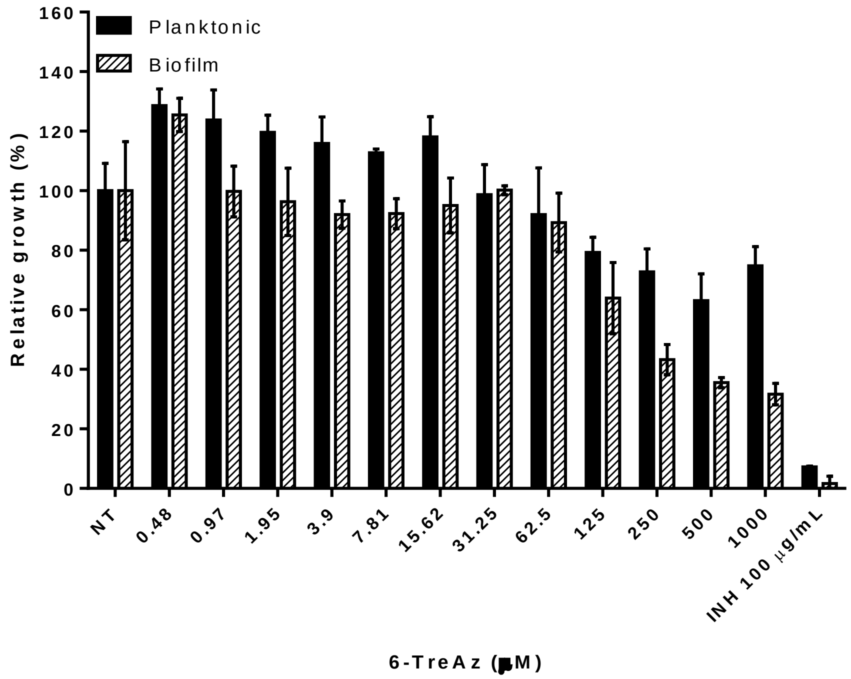

Figure S8. Dose response of 6-TreAz against Msmeg planktonic and biofilm growth.

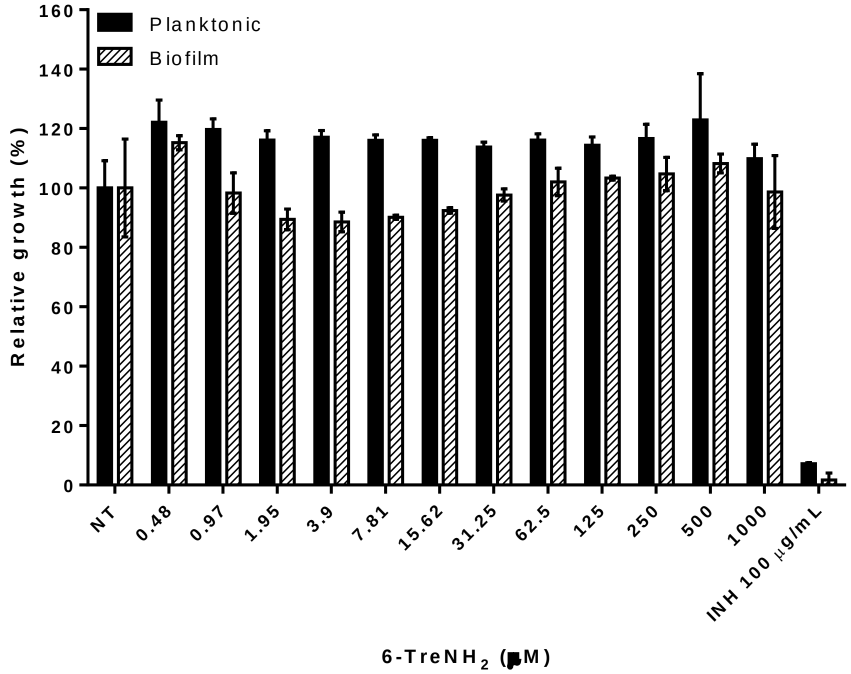

Figure S9. Dose response of 6-TreNH$_2$ against Msmeg planktonic and biofilm growth.

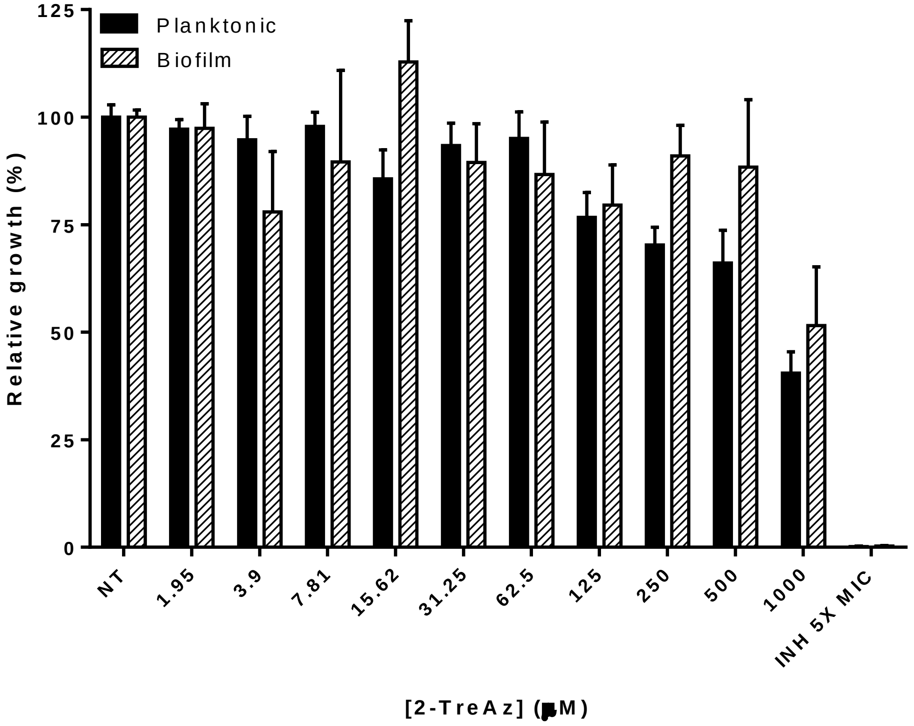

Figure S10. Dose response of 2-TreAz against Mtb H37Rv planktonic and biofilm growth.

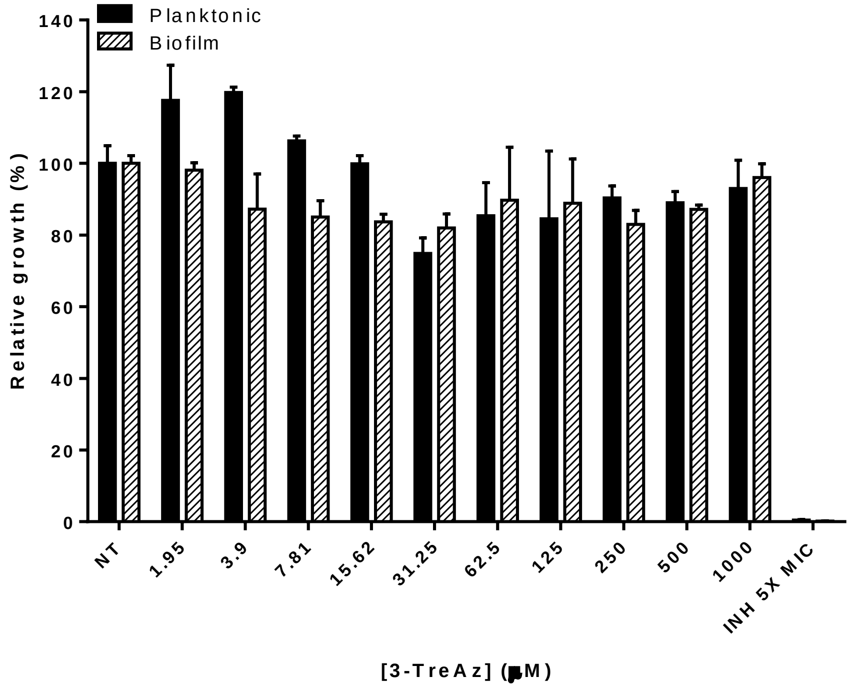

Figure S11. Dose response of 3-TreAz against Mtb H37Rv planktonic and biofilm growth.

8

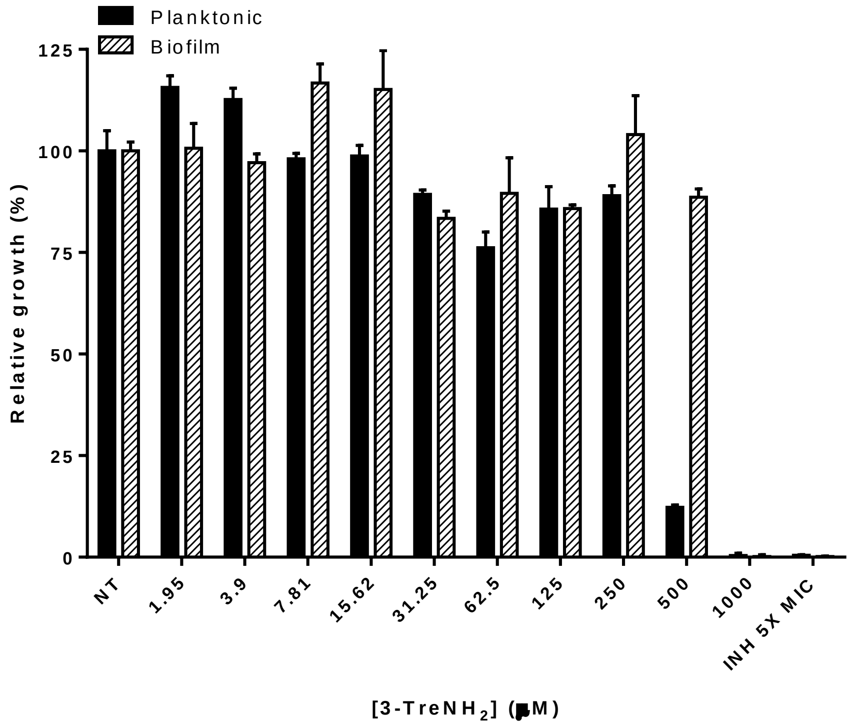

Figure S12. Dose response of 3-TreNH$_2$ against Mtb H37Rv planktonic and biofilm growth.

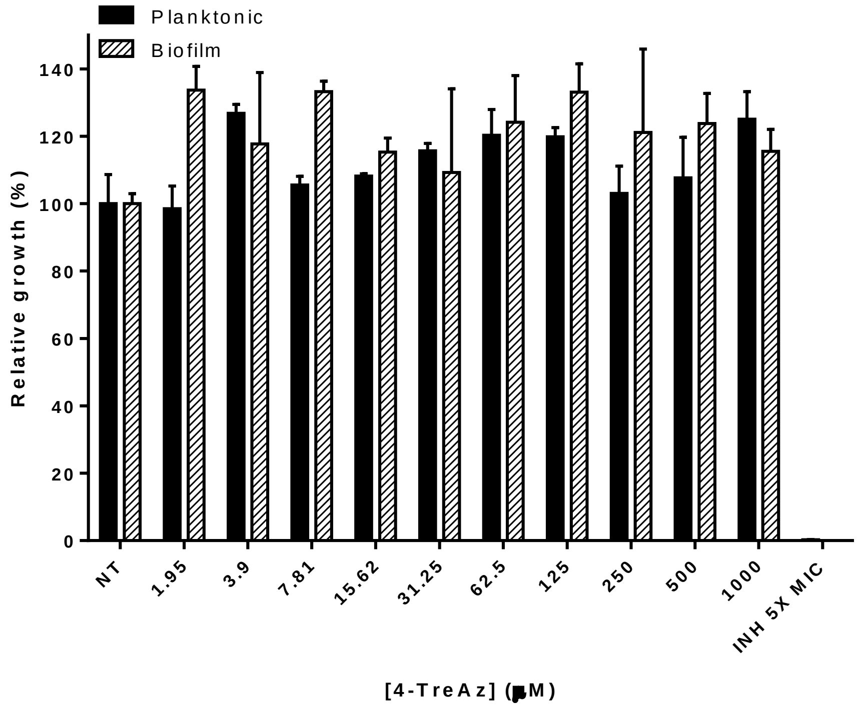

Figure S13. Dose response of 4-TreAz against Mtb H37Rv planktonic and biofilm growth.

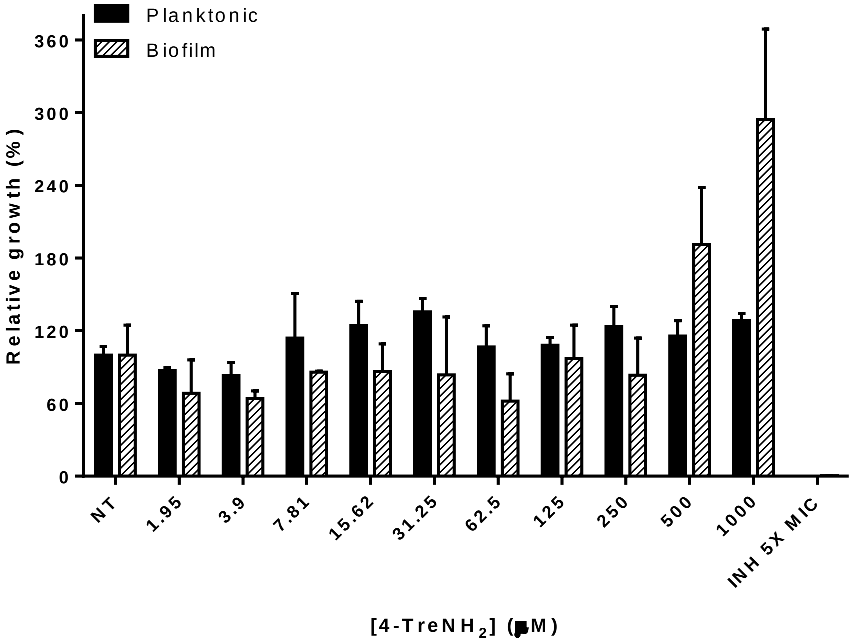

Figure S14. Dose response of 4-TreNH2 against Mtb mc^{2}7000 planktonic and biofilm growth.

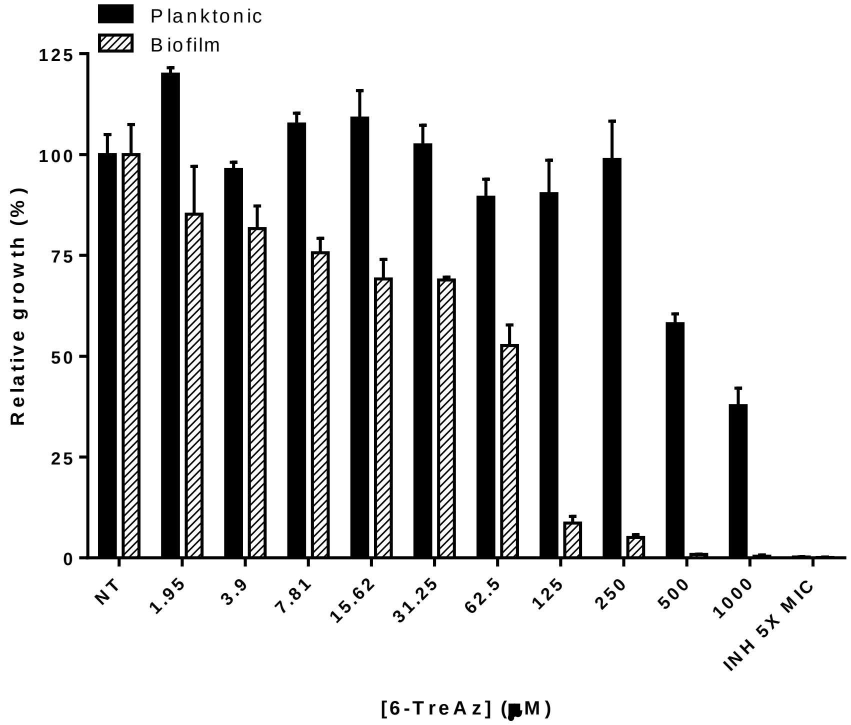

Figure S15. Dose response of 6-TreAz against Mtb H37Rv planktonic and biofilm growth.

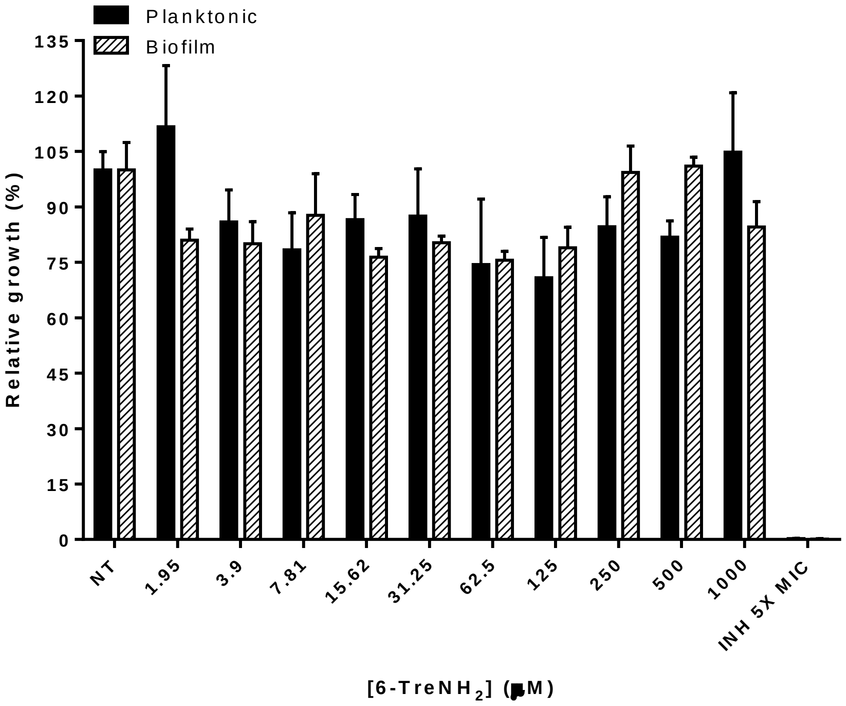

Figure S16. Dose response of 6-TreNH$_2$ against Mtb H37Rv planktonic and biofilm growth.

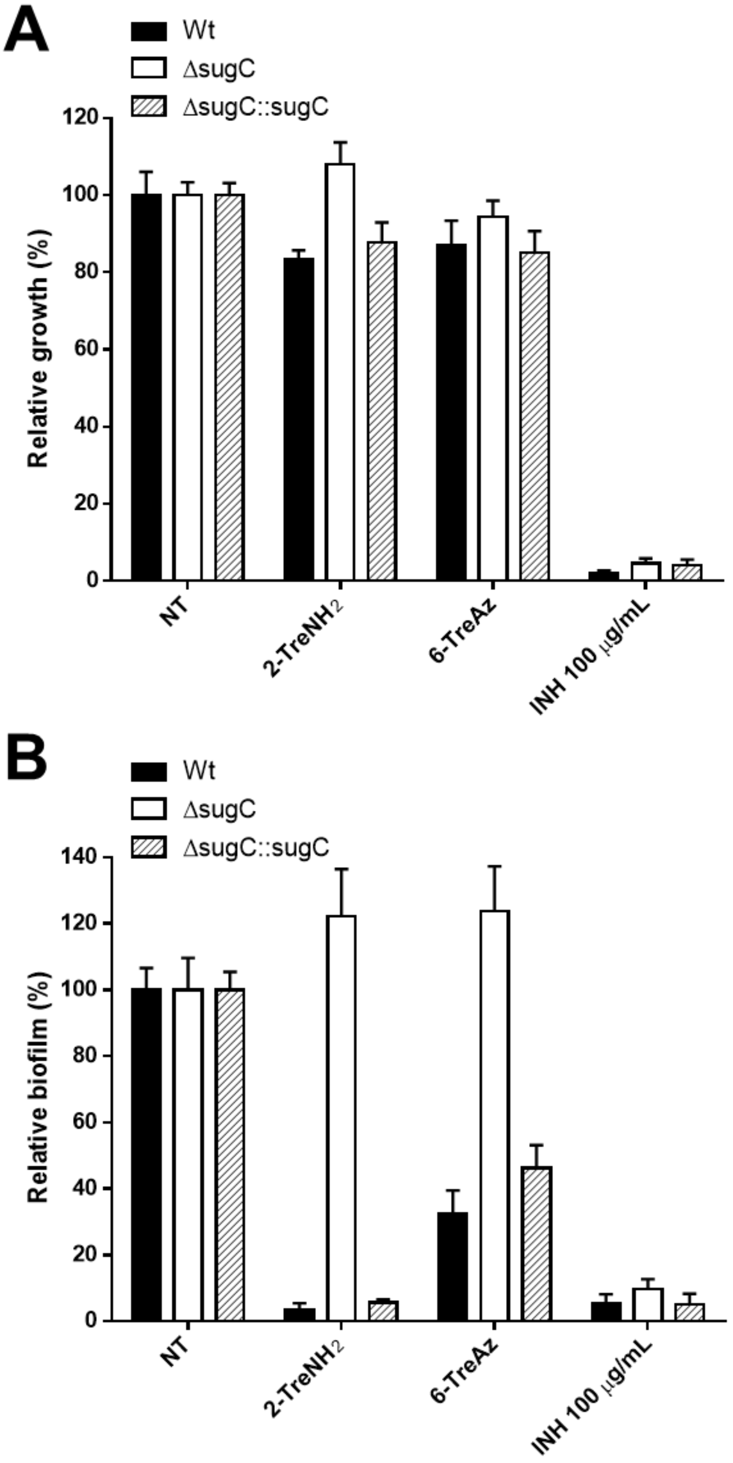

Figure S17. Msmeg wild type or mutant was cultured under planktonic (A) or biofilm (B) growth conditions in the presence of the indicated trehalose analogue, or left not treated (NT) as negative control, or treated with isoniazid (INH) as positive control. Growth was measured using OD_{600} reading for planktonic conditions and CV staining for biofilm conditions. Analogue concentrations are: 2-TreNH$_2$, 31.25 μM; 6-TreAz, 500 μM. Data are normalized relative to positive control at 100%. Error bars represent the standard deviation of three replicates and data are representative of three independent experiments.

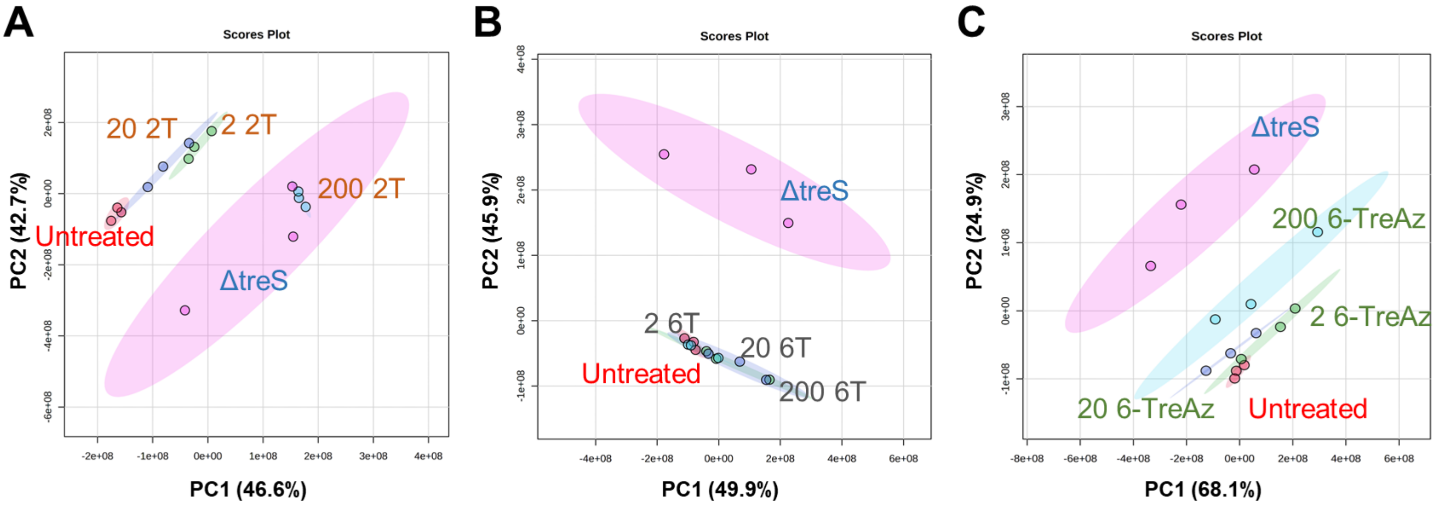

Figure S18. Biofilm metabolomics analysis using principal component analysis (PCA) plots. Metabolomes from Mtb biofilm-persisters after treatment with 0, 2, 20, and 200 μg/mL 2- or 6-TreNH$_2$ (2T and 6T in panels A and B, respectively) were collected and analyzed by LC-MS. ΔtreS and treatment with same amount of 6-TreAz (panel C) were used as positive controls.

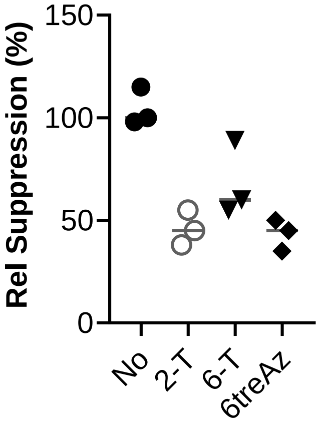

Figure S19. The effect of trehalose analogues on TreS-mediated maltose production was monitored using an in vitro enzyme reaction as reported in Lee *et al*. *Nat. Commun.* **2019**, 2928-2928. In vitro TreS reactions contained 0.1 μg of recombinant TreS, 10 mM $MgCl_2$, 0.1 mM trehalose in the presence or absence of 100 μM analogue, incubated at 37 °C for 4 h. Maltose production was monitored by LC-MS. 2-T, 2-TreNH$_2$; 6-T, 6-TreNH$_2$.

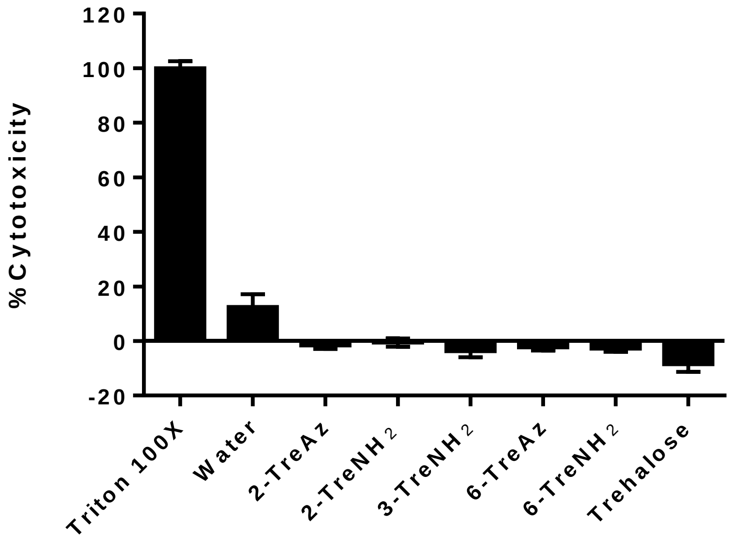

Figure S20. TreAz and $TreNH_2$ analogues are non-cytotoxic to U-937 pro-monocyte cell line. U-937 cells were treated with 1 mM concentration of the indicated trehalose analogue for 48 h and lactate dehydrogenase (LDH) release was assayed. 10% Triton 100X was used for the maximum LDH release control and cell culture grade water was used for the spontaneous LDH release control. Data shown are normalized to maximum LDH release as 100% and error bars represent three replicates. Data are representative of two independent experiments.

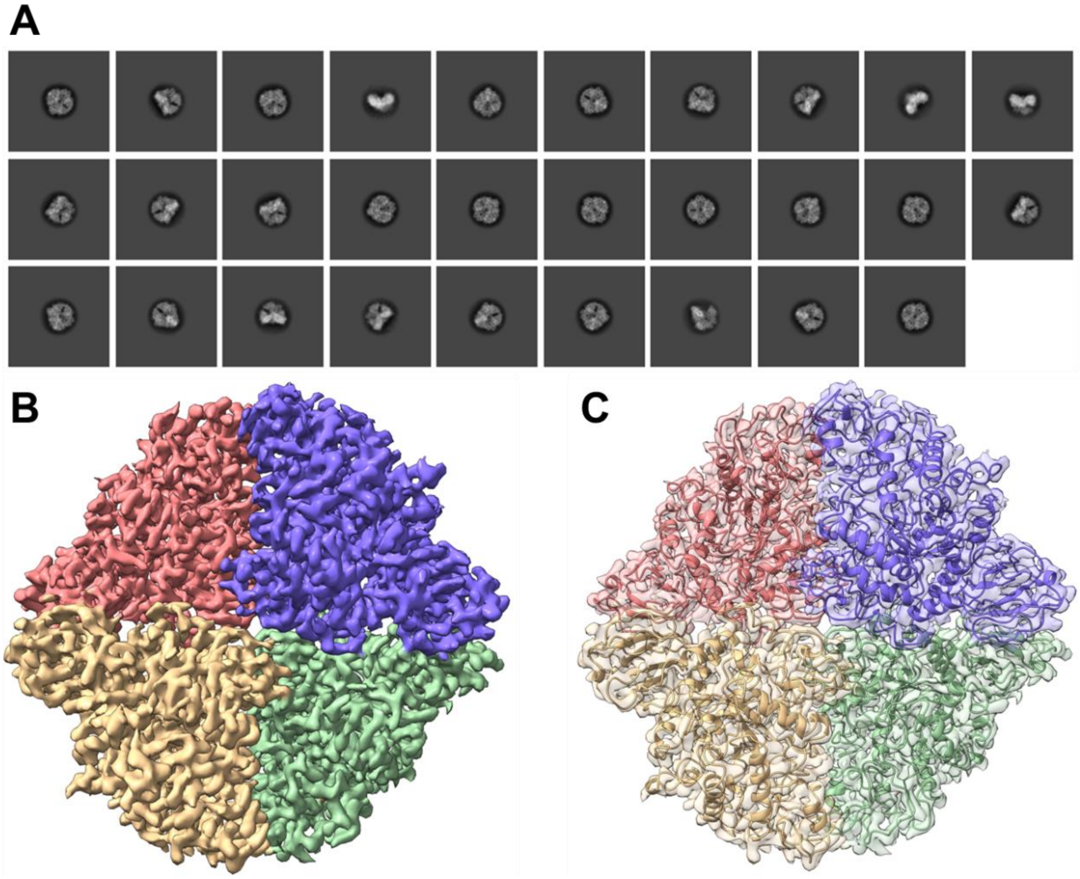

Figure S21. Data analysis and 3D re-construction of the TreS-D238A/6-TreAz complex. (A) 2D classification. (B) Cryo-EM volume map. (C) TreS model fitted to the map.

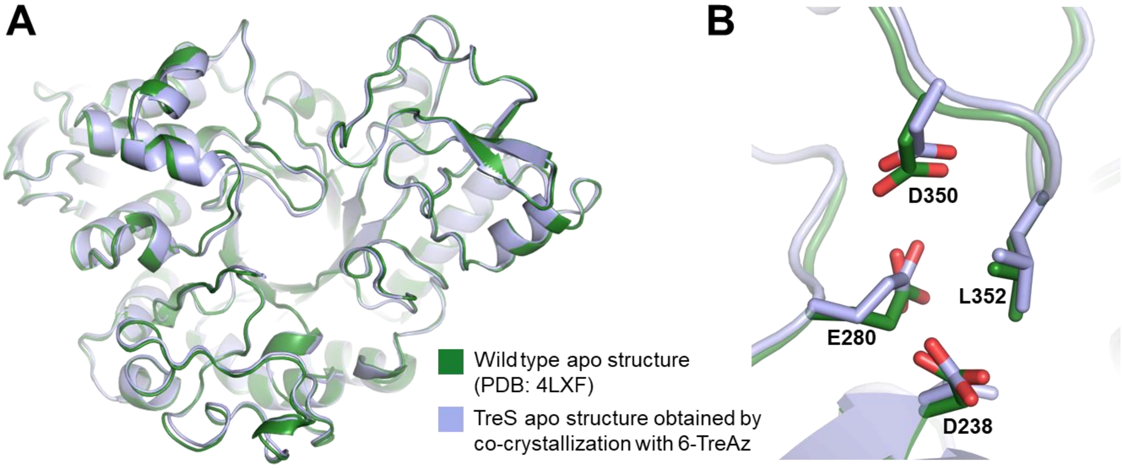

Figure S22. Alignemnt of the TreS apo structure (PDB:4LXF) to the crystal structure solved for wild-type TreS incubated with 6-TreAz. (A) Overall alignment and (B) close up of the catalytic amino acid residues at the active site and L352.

Supplementary tables

Table S1. Metabolic pathway enrichment. Significantly altered pathways in Mtb affected by treatment with putative TreS inhibitors. ΔtreS was included as a control.

	**ΔtreS**	**2-TreNH$_2$**	**6-TreAz**	**6- TreNH$_2$**
1	Purine metabolism	Glycerophospholipid metabolism	Purine metabolism	Glycerophospholipid metabolism
2	Glycerophospholipid metabolism	Purine metabolism	Alanine, aspartate and glutamate metabolism	**Glycerolipid metabolism**
3	Lysine biosynthesis	**Arginine and proline metabolism**	Lysine biosynthesis	**Arginine and proline metabolism**
4	Glycine, serine and threonine metabolism	Lysine biosynthesis	D-Glutamine and D-glutamate metabolism	
5	Alanine, aspartate and glutamate metabolism	**Sulfur metabolism**	Nicotinate and nicotinamide metabolism	
6	Lysine biosynthesis	**Cysteine and methionine metabolism**	Arginine biosynthesis	
7	Starch and sucrose metabolism	**Carbapenem biosynthesis**	Glycerophospholipid metabolism	
8	Streptomycin biosynthesis	Pentose phosphate pathway		
9	Lysine degradation	Starch and sucrose metabolism		
10	Phenylalanine metabolism	Streptomycin biosynthesis		
11	Pentose phosphate pathway	Alanine, aspartate and glutamate metabolism		
12	Nicotinate and nicotinamide metabolism	Lysine biosynthesis		
13	D-Glutamine and D-glutamate metabolism	Inositol phosphate metabolism		
14	Inositol phosphate metabolism	Arginine biosynthesis		
15	Arginine biosynthesis	D-Glutamine and D-glutamate metabolism		
16	Citrate cycle (TCA cycle)	Fructose and mannose metabolism		
17	Fructose and mannose metabolism	**Pentose and glucuronate interconversions**		
18	Tyrosine metabolism	Nicotinate and nicotinamide metabolism		
19	Methane metabolism			

16

2-TreAz

1H NMR (500 MHz, D2O)

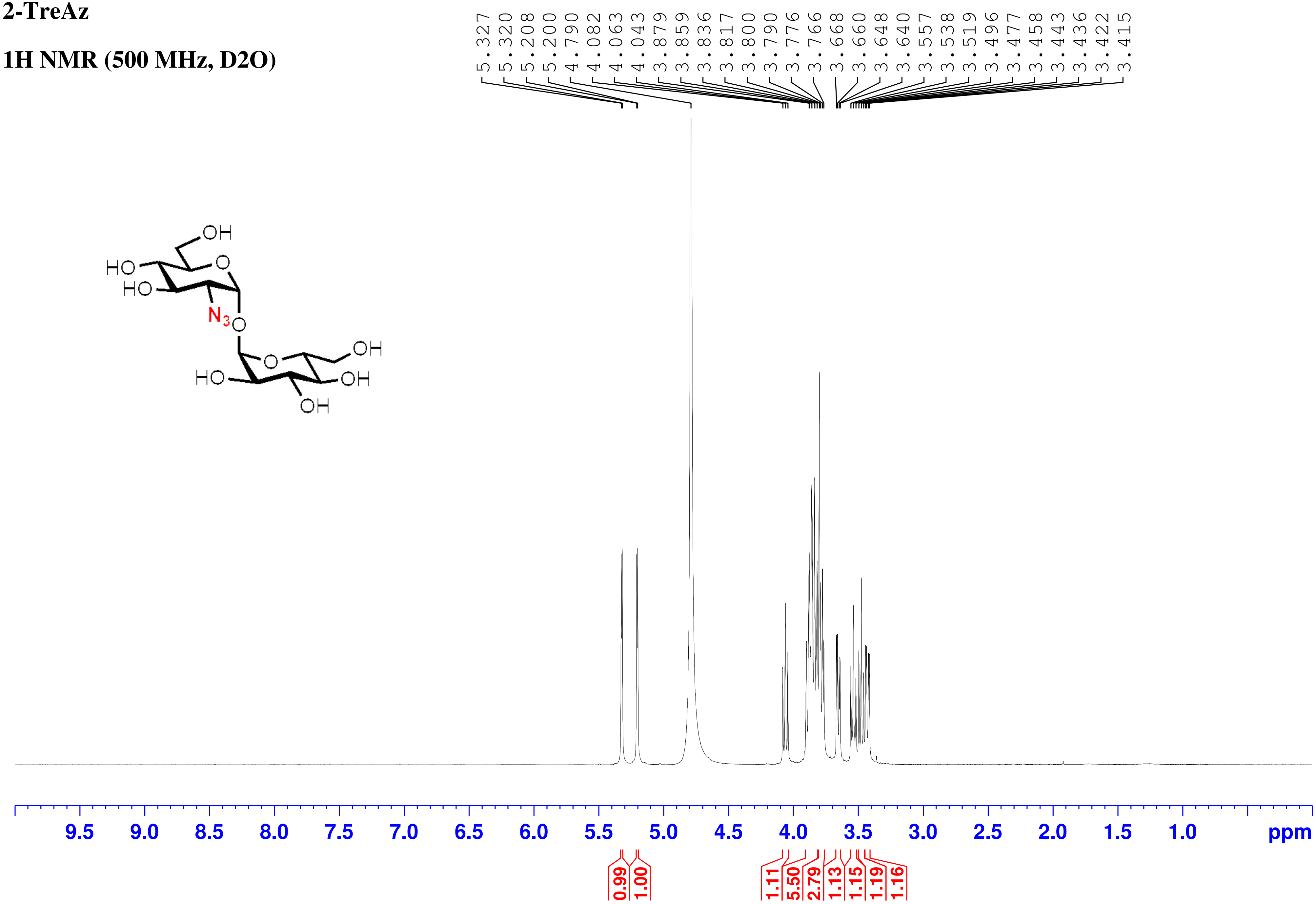

2-TreAz

1H NMR (500 MHz, D2O)

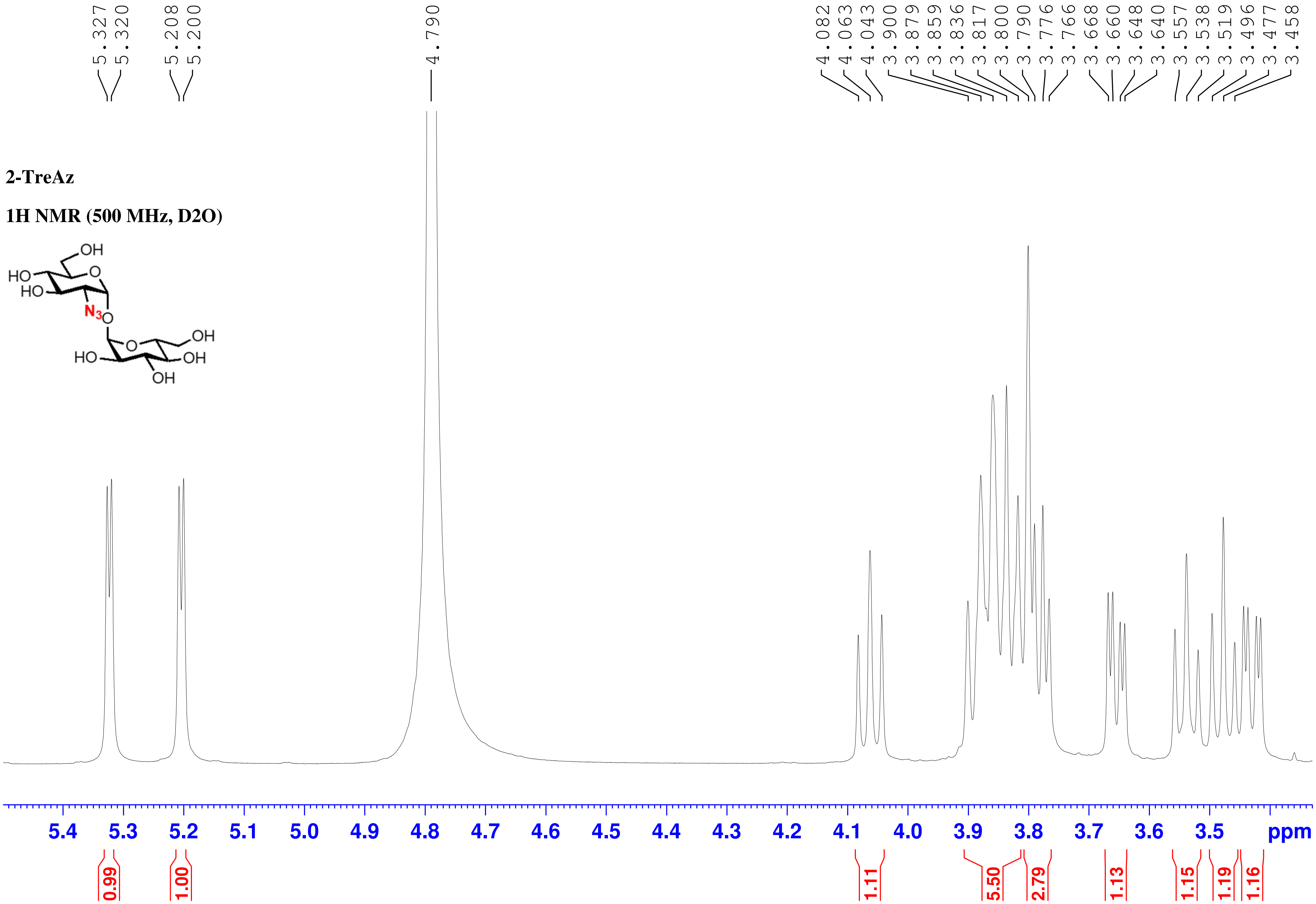

2-TreAz

13C NMR (126 MHz, D2O)

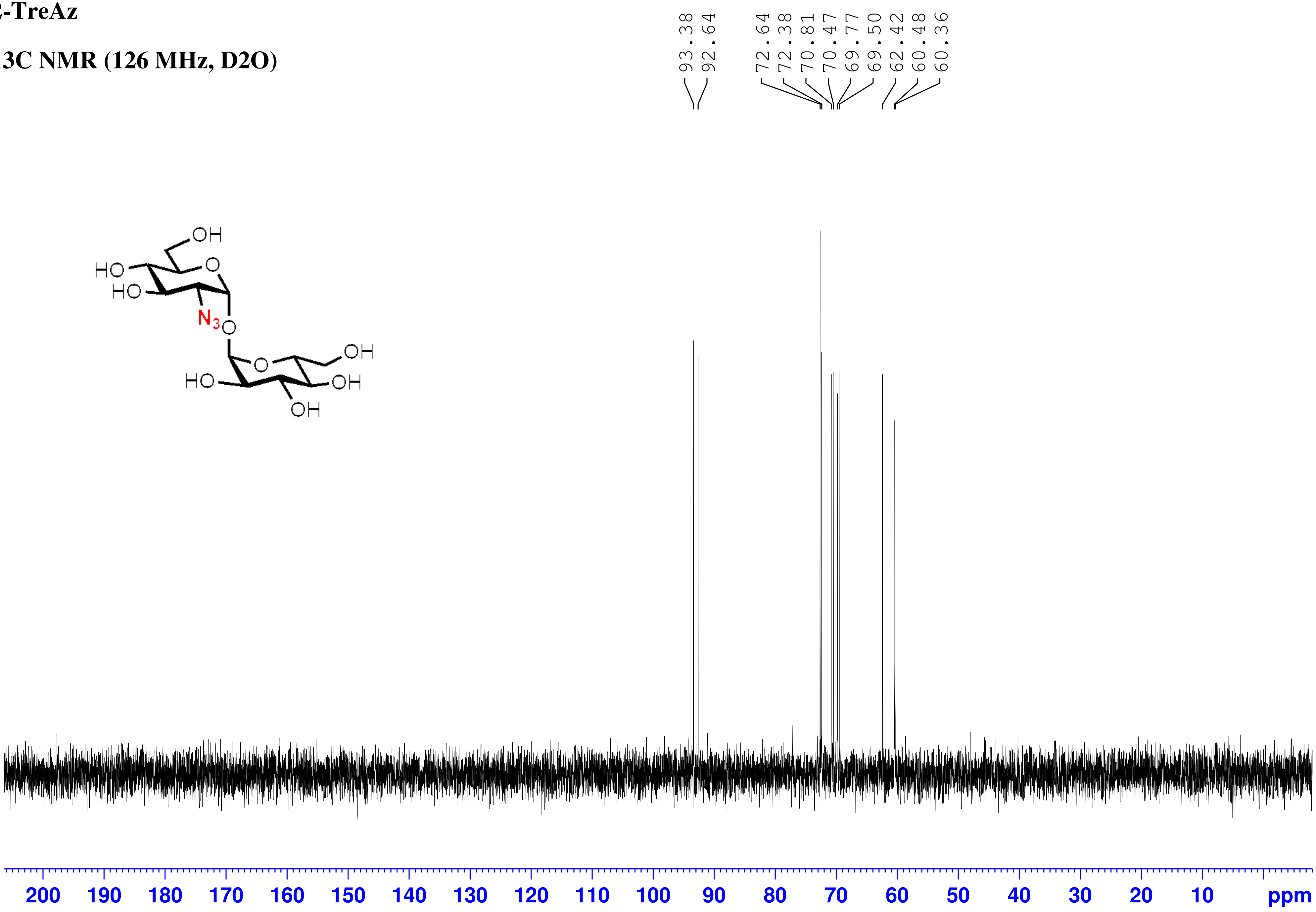

2-TreAz

13C NMR (126 MHz, D2O)

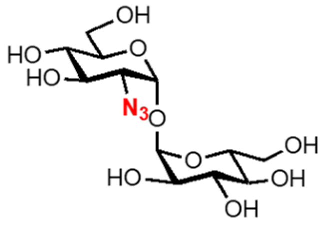

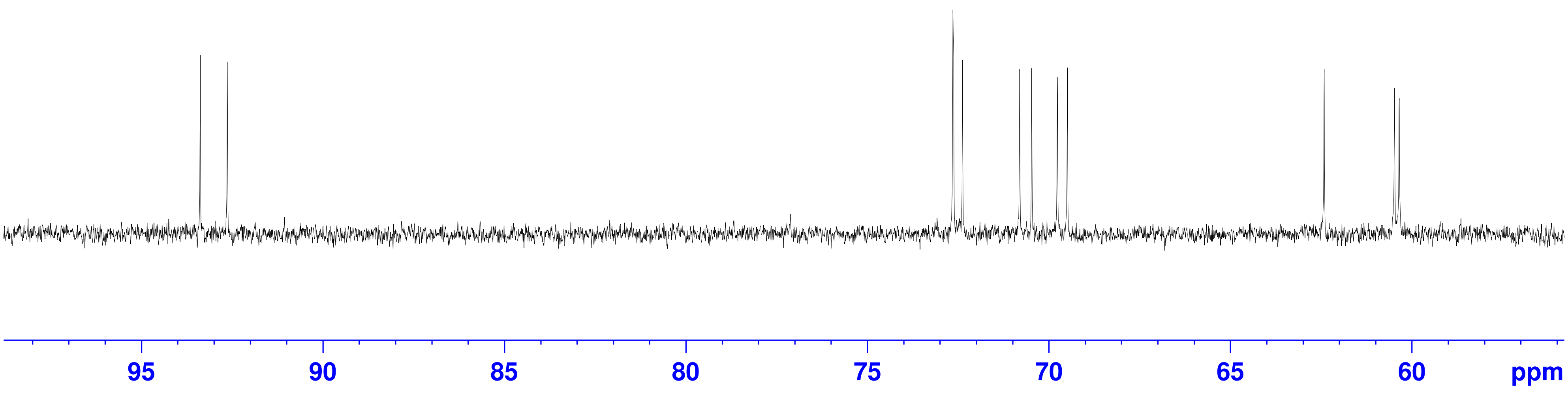

3-TreAz

1H NMR (500 MHz, D2O)

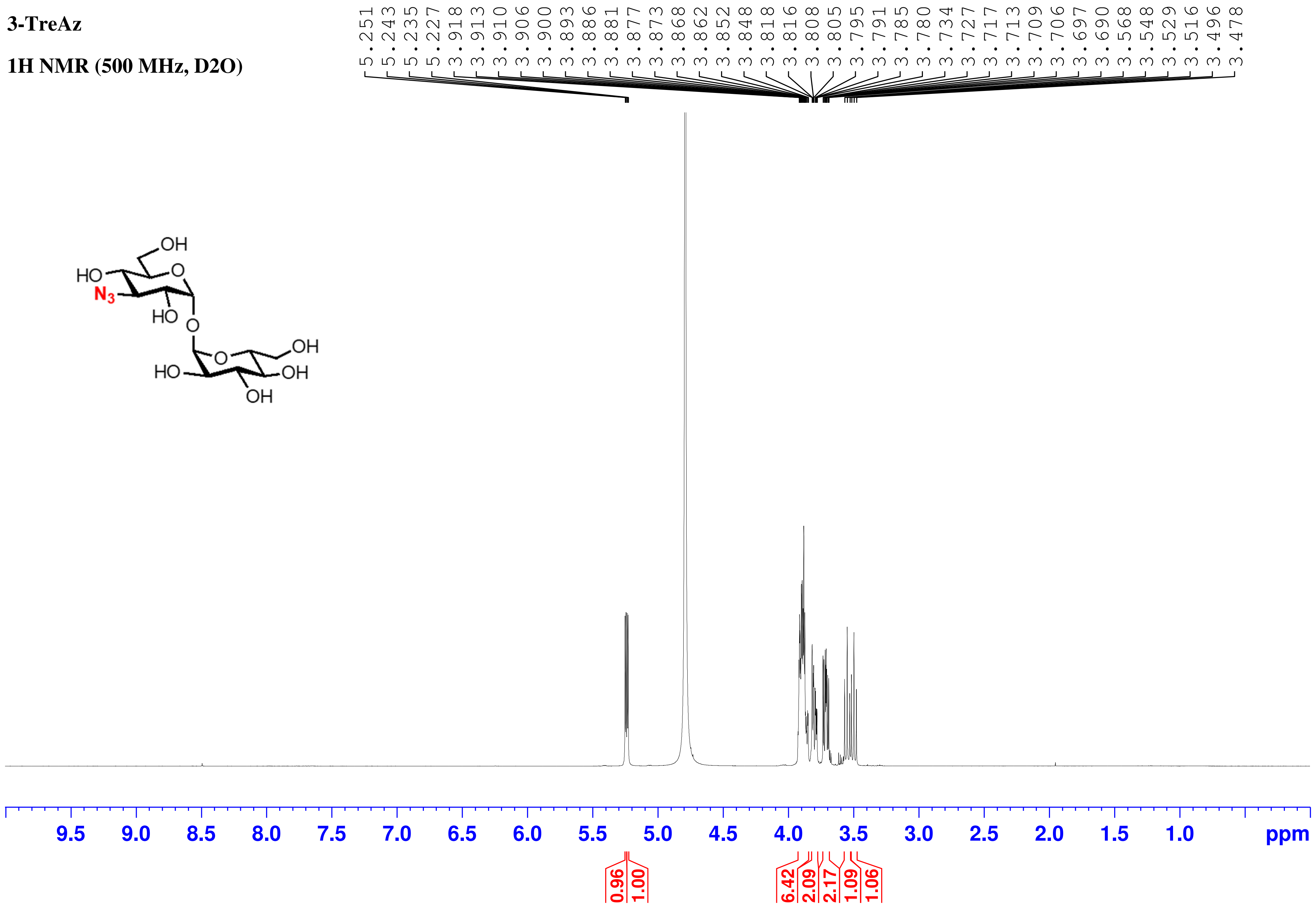

3-TreAz

1H NMR (500 MHz, D2O)

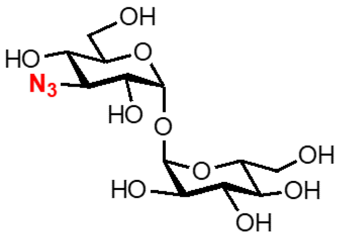

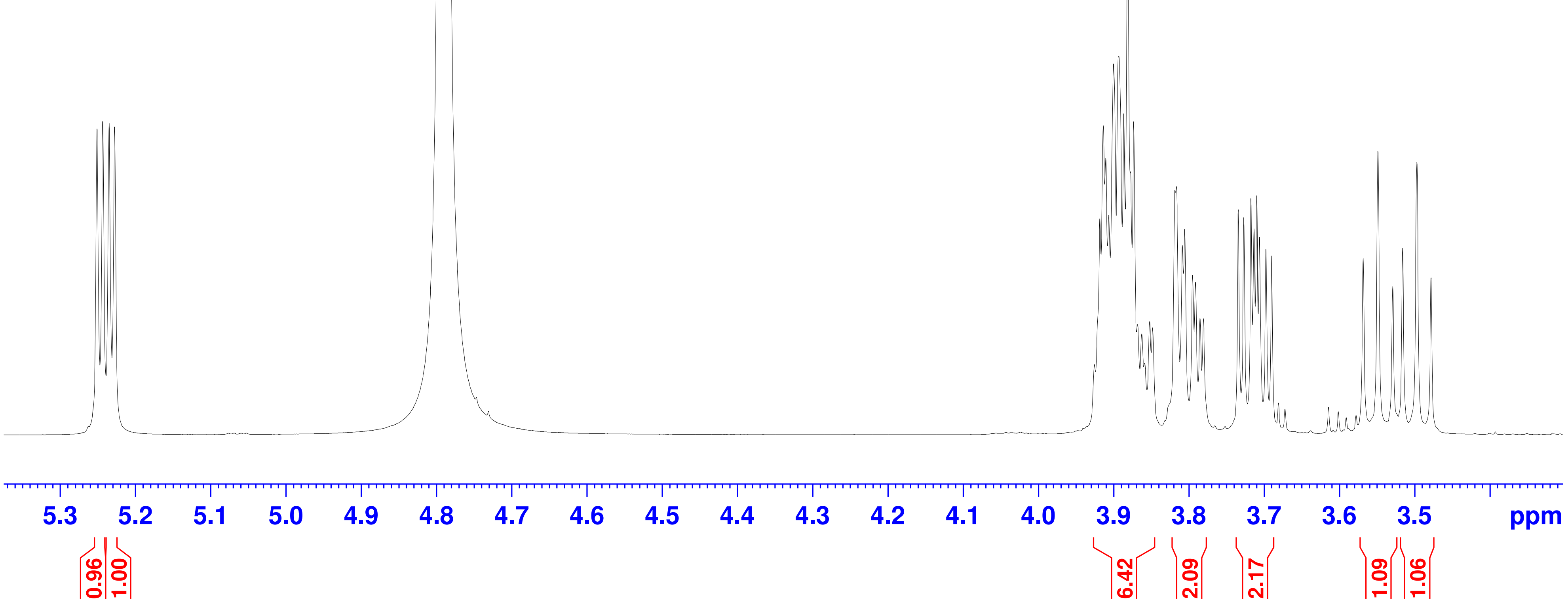

3-TreAz

13C NMR (126 MHz, D2O)

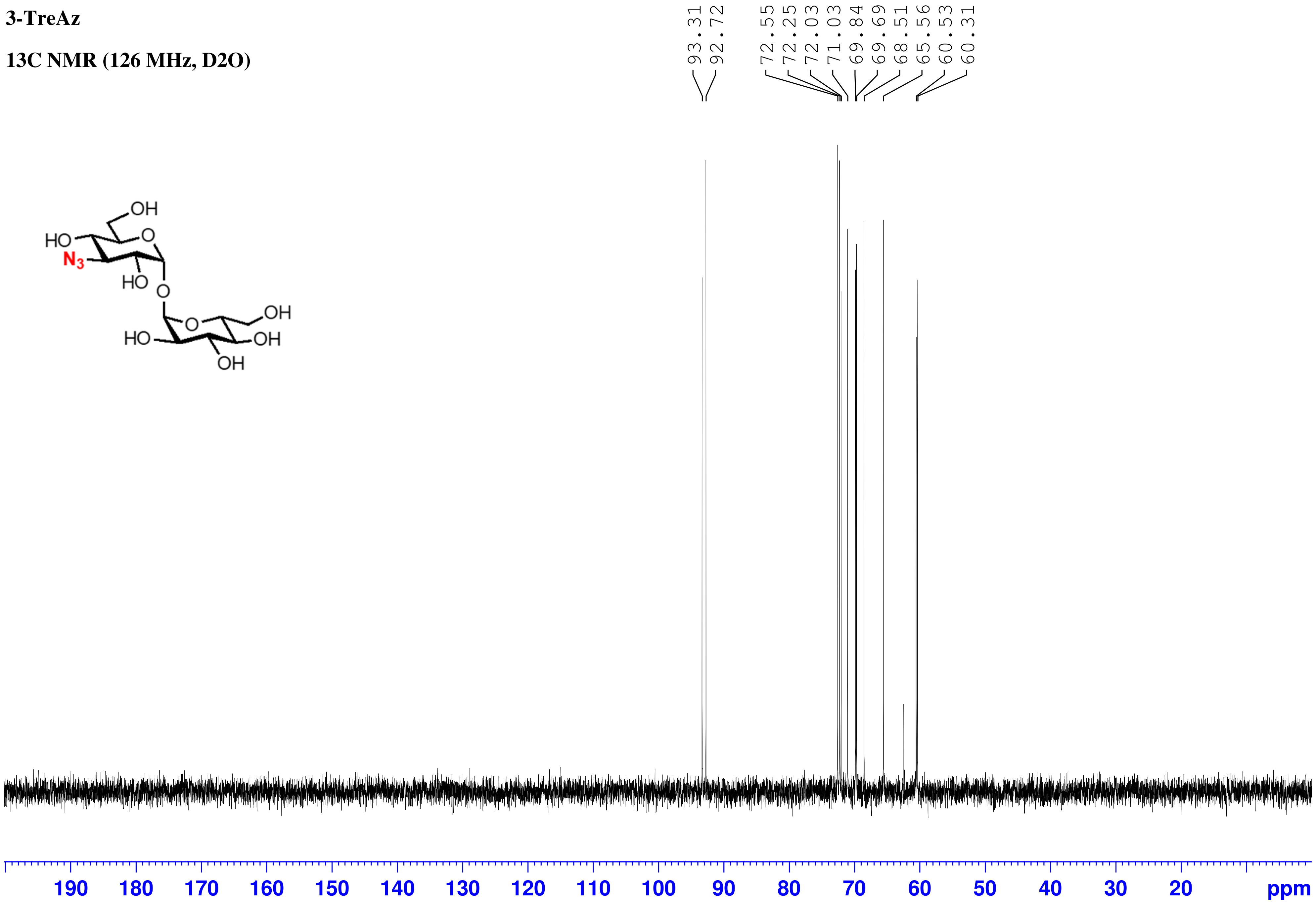

3-TreAz

13C NMR (126 MHz, D2O)

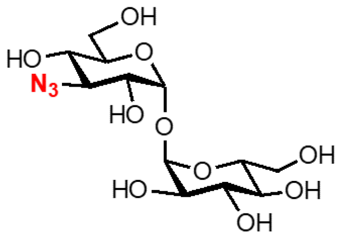

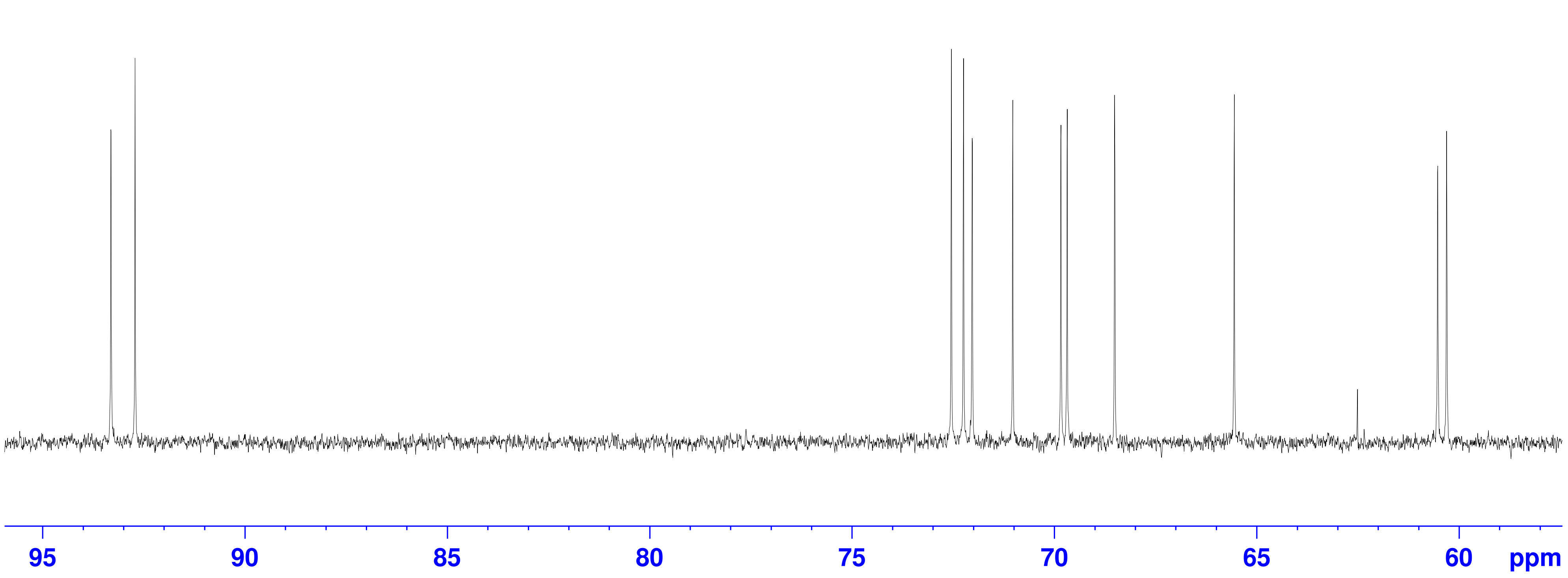

4-TreAz

1H NMR (500 MHz, D2O)

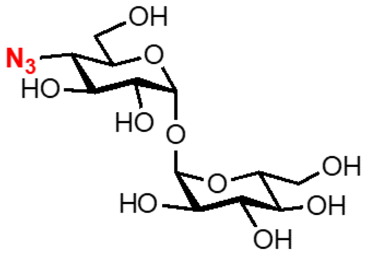

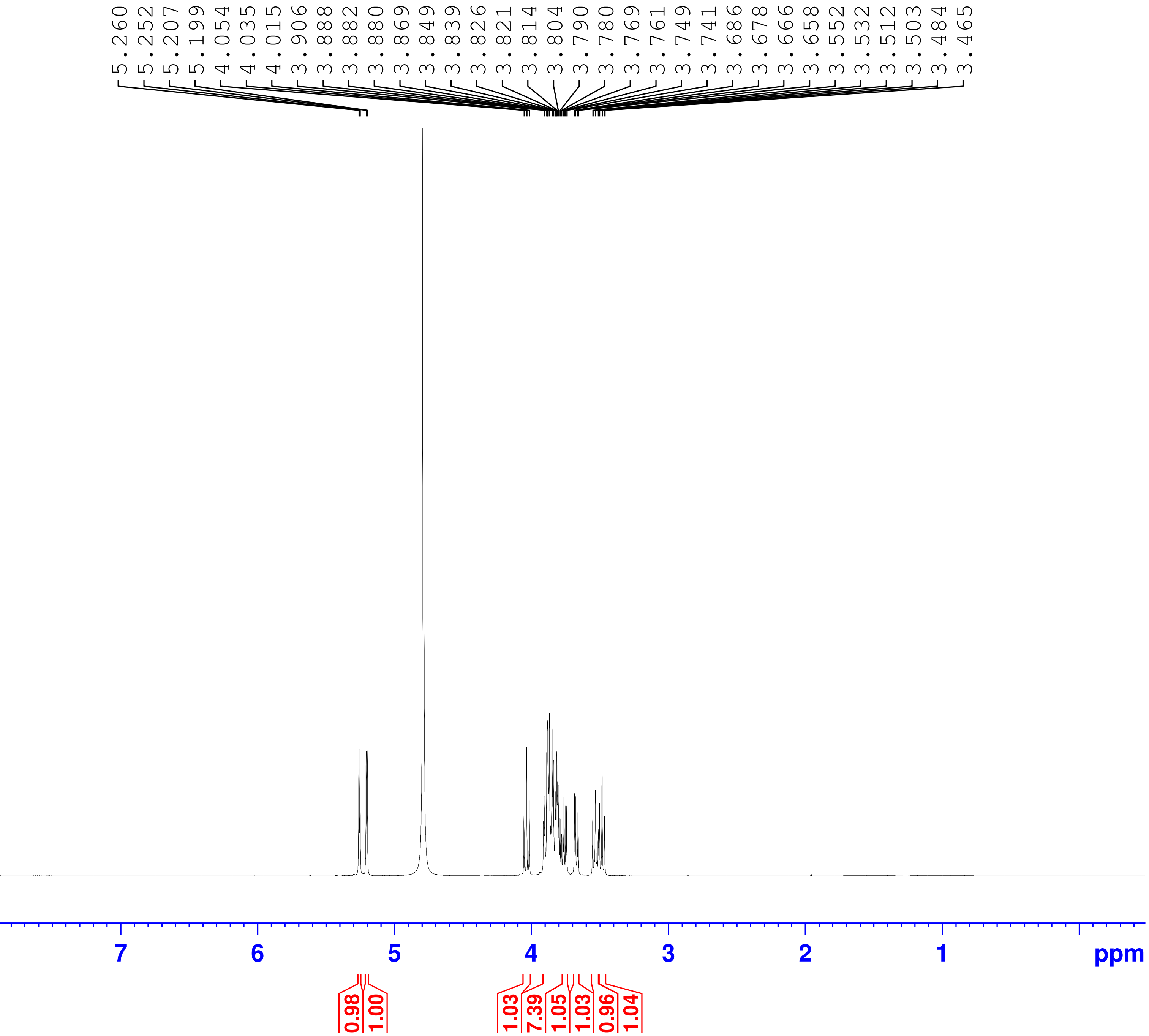

4-TreAz

1H NMR (500 MHz, D2O)

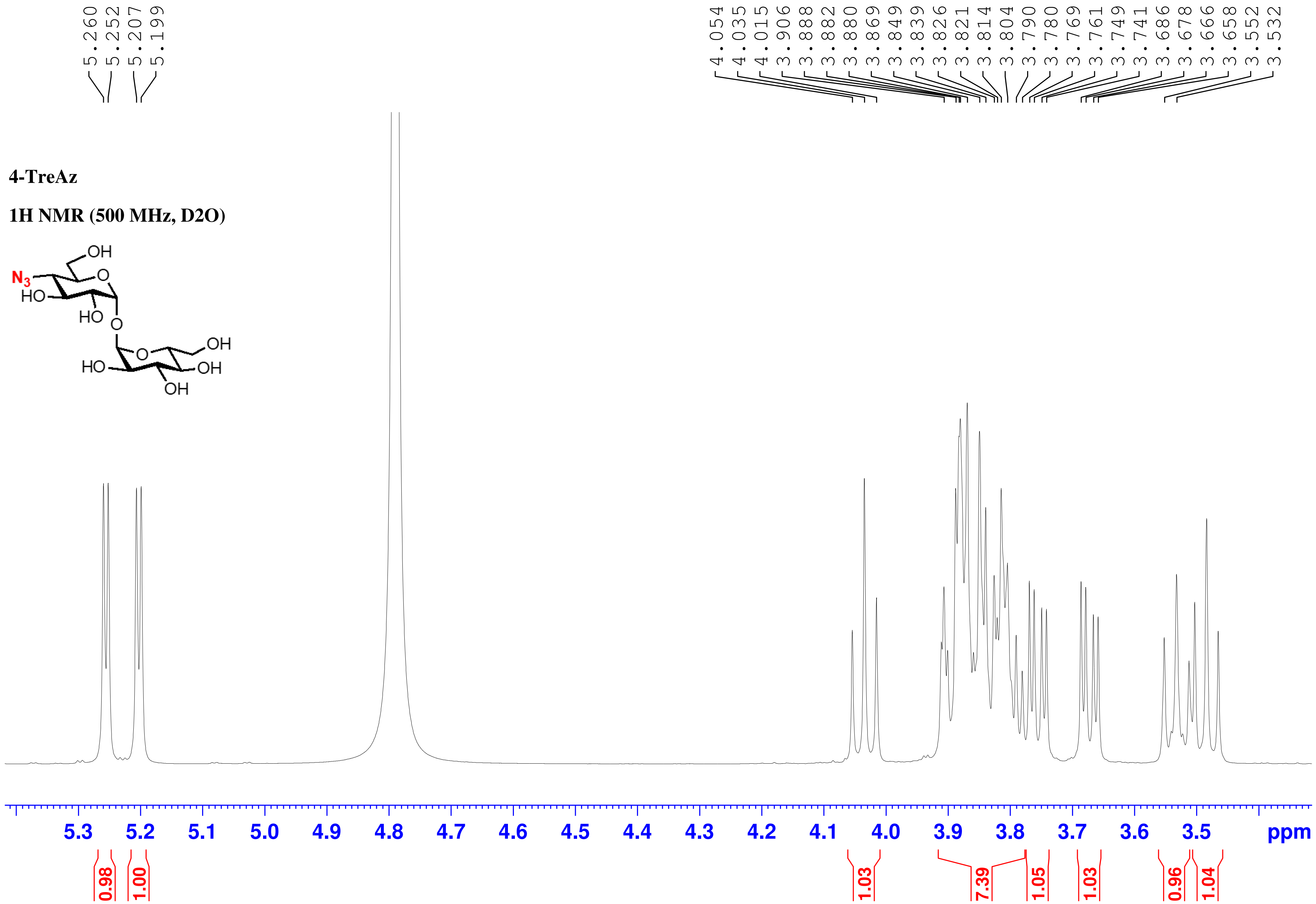

4-TreAz

13C NMR (126 MHz, D2O)

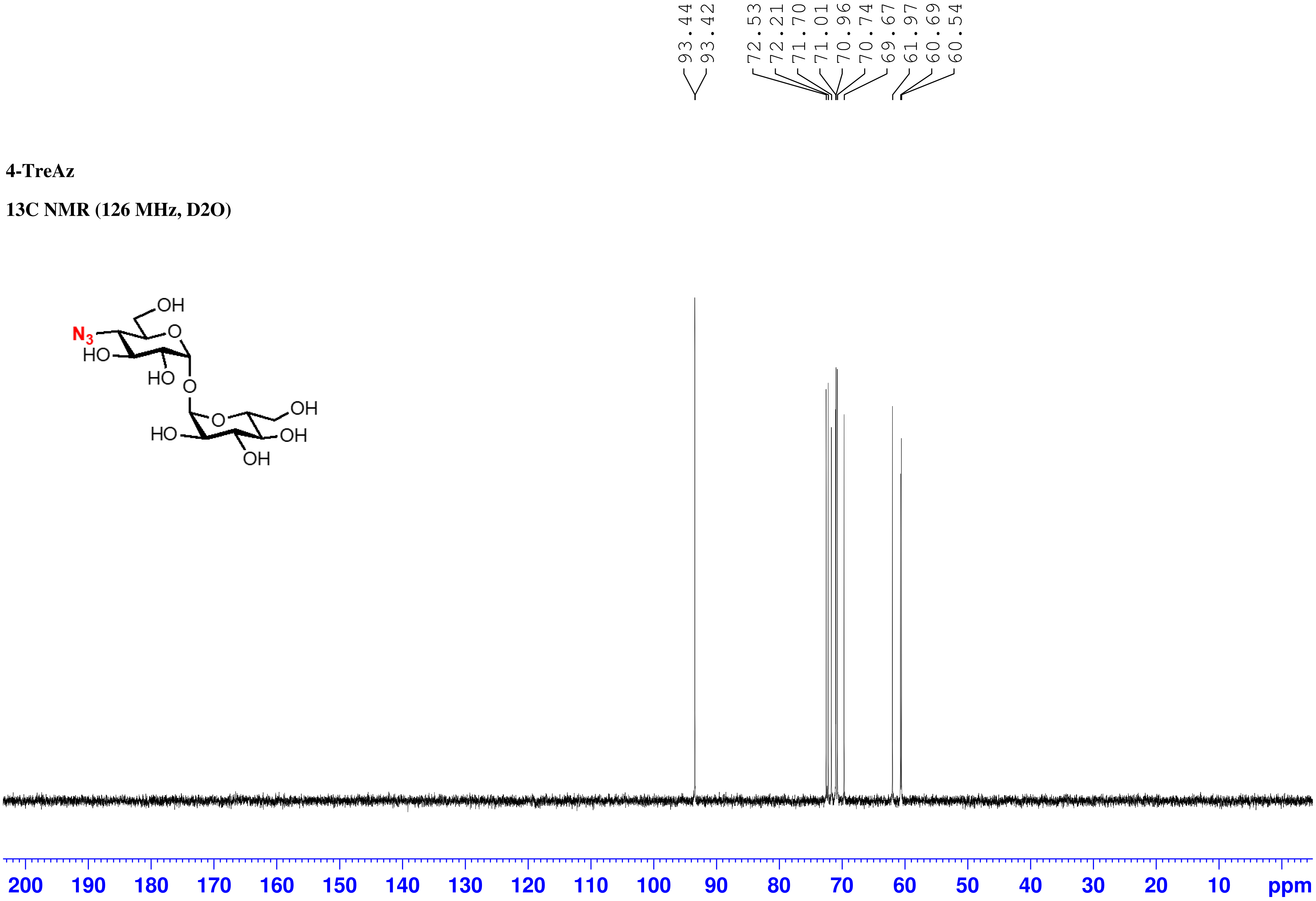

4-TreAz

13C NMR (126 MHz, D2O)

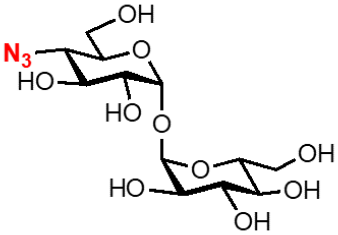

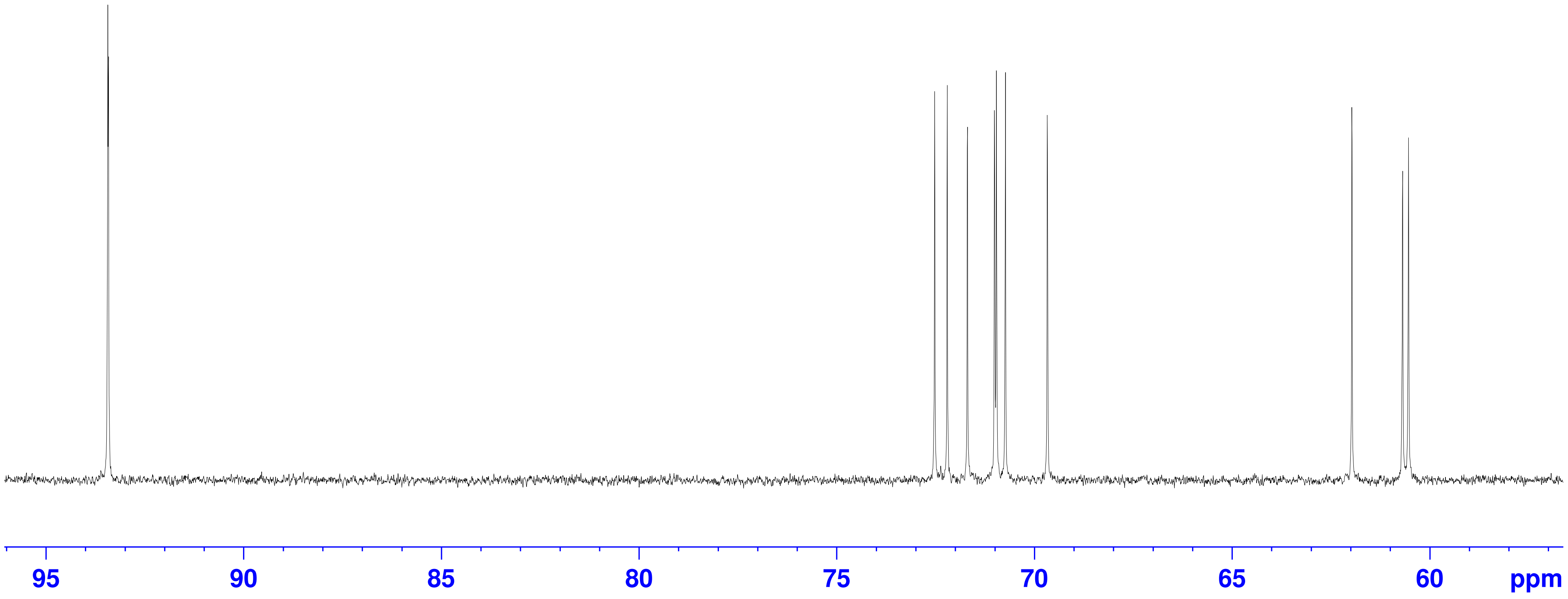

6-TreAz

1H NMR (500 MHz, D2O)

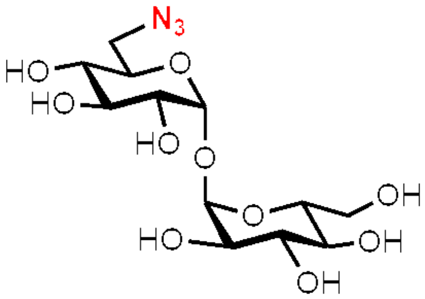

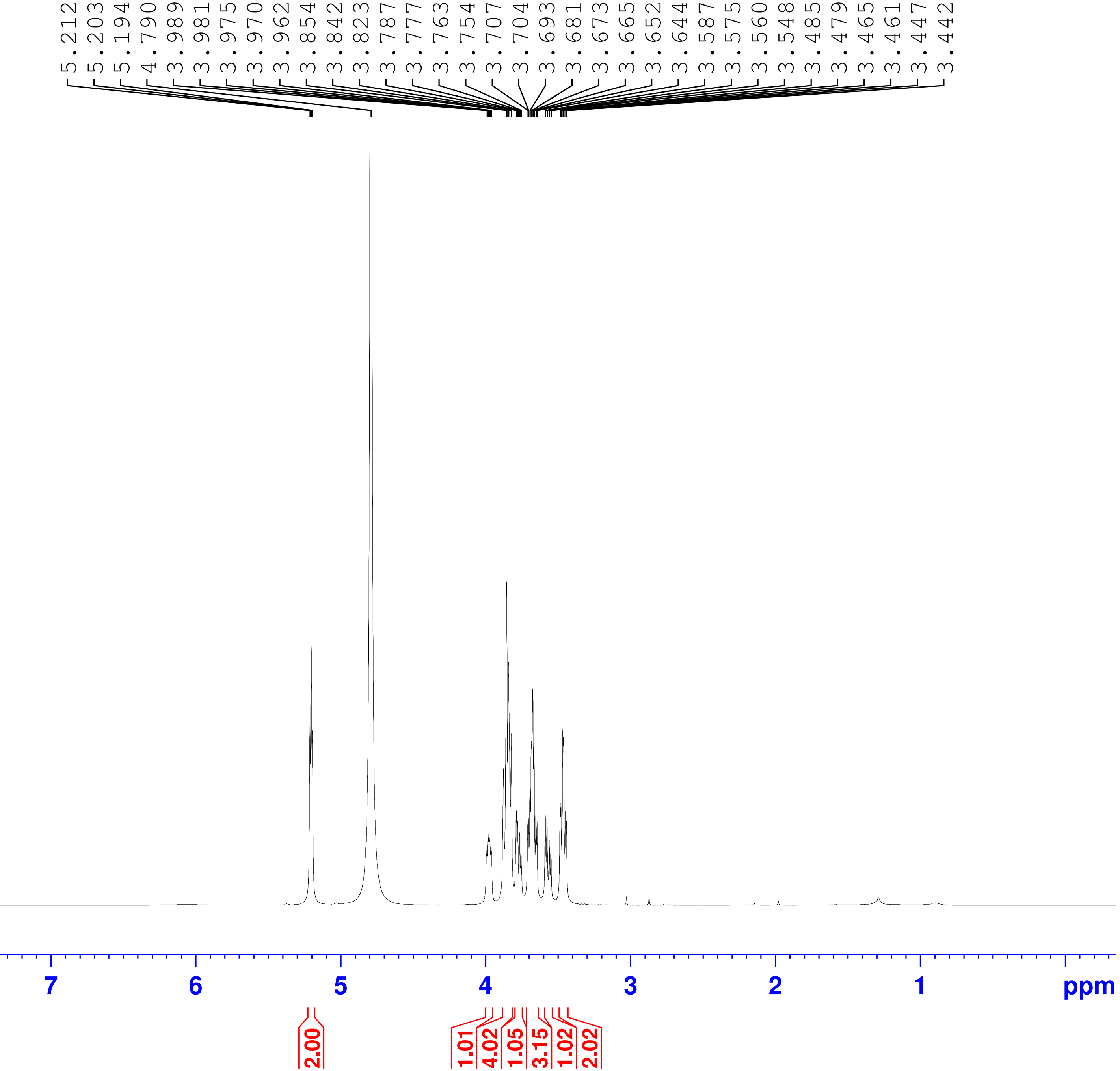

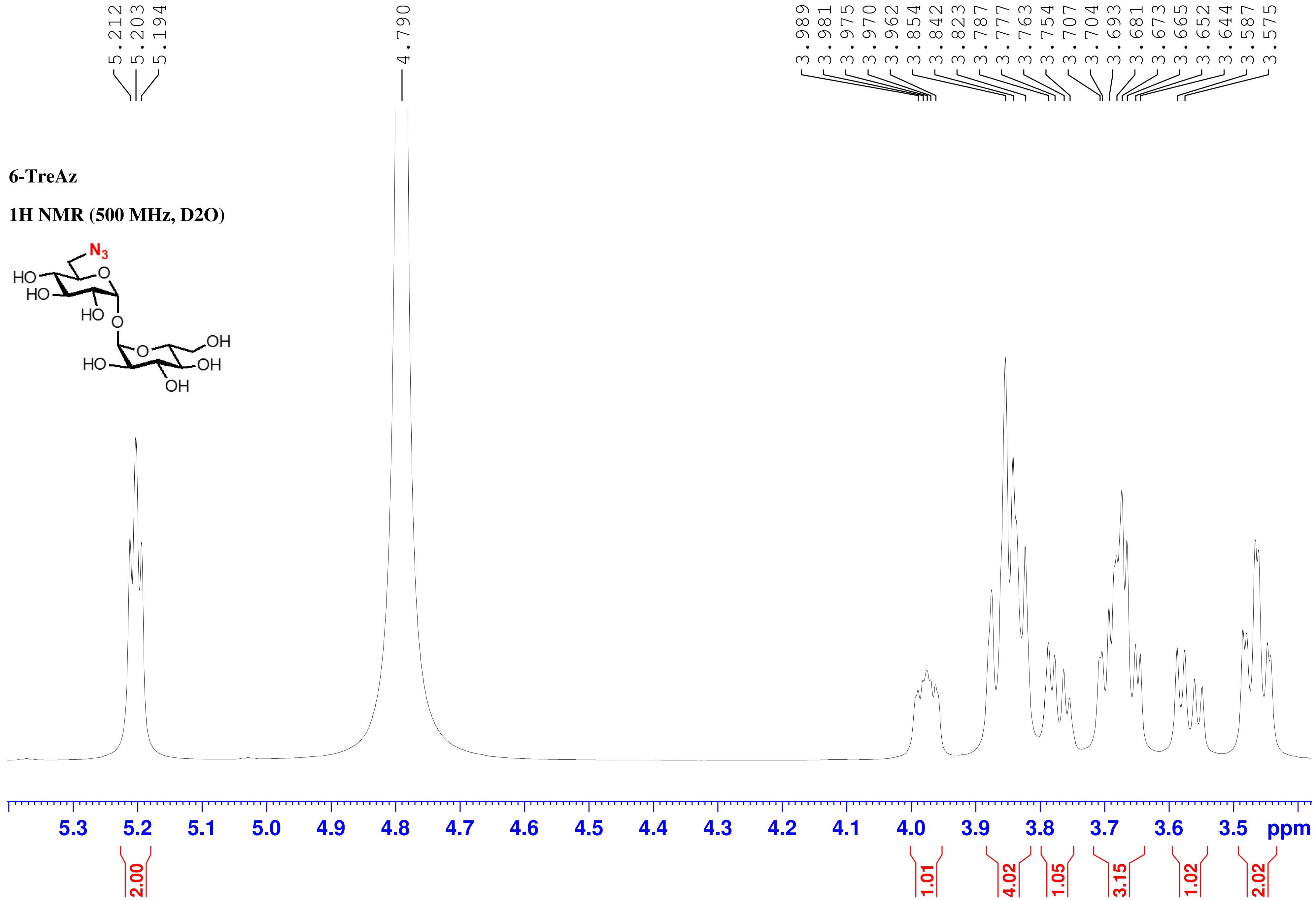
6-TreAz
1H NMR (500 MHz, D2O)
N3
HO
HO
HO
O
O
O
OH
OH
HO
OH
5.212
5.203
5.194
4.790
3.989
3.981
3.975
3.970
3.962
3.854
3.842
3.823
3.787
3.777
3.763
3.754
3.707
3.704
3.693
3.681
3.673
3.665
3.652
3.644
3.587
3.575
5.3
5.2
5.1
5.0
4.9
4.8
4.7
4.6
4.5
4.4
4.3
4.2
4.1
4.0
3.9
3.8
3.7
3.6
3.5
ppm
2.00
1.01
4.02
1.05
3.15
1.02
2.02

6-TreAz

13C NMR (126 MHz, D2O)

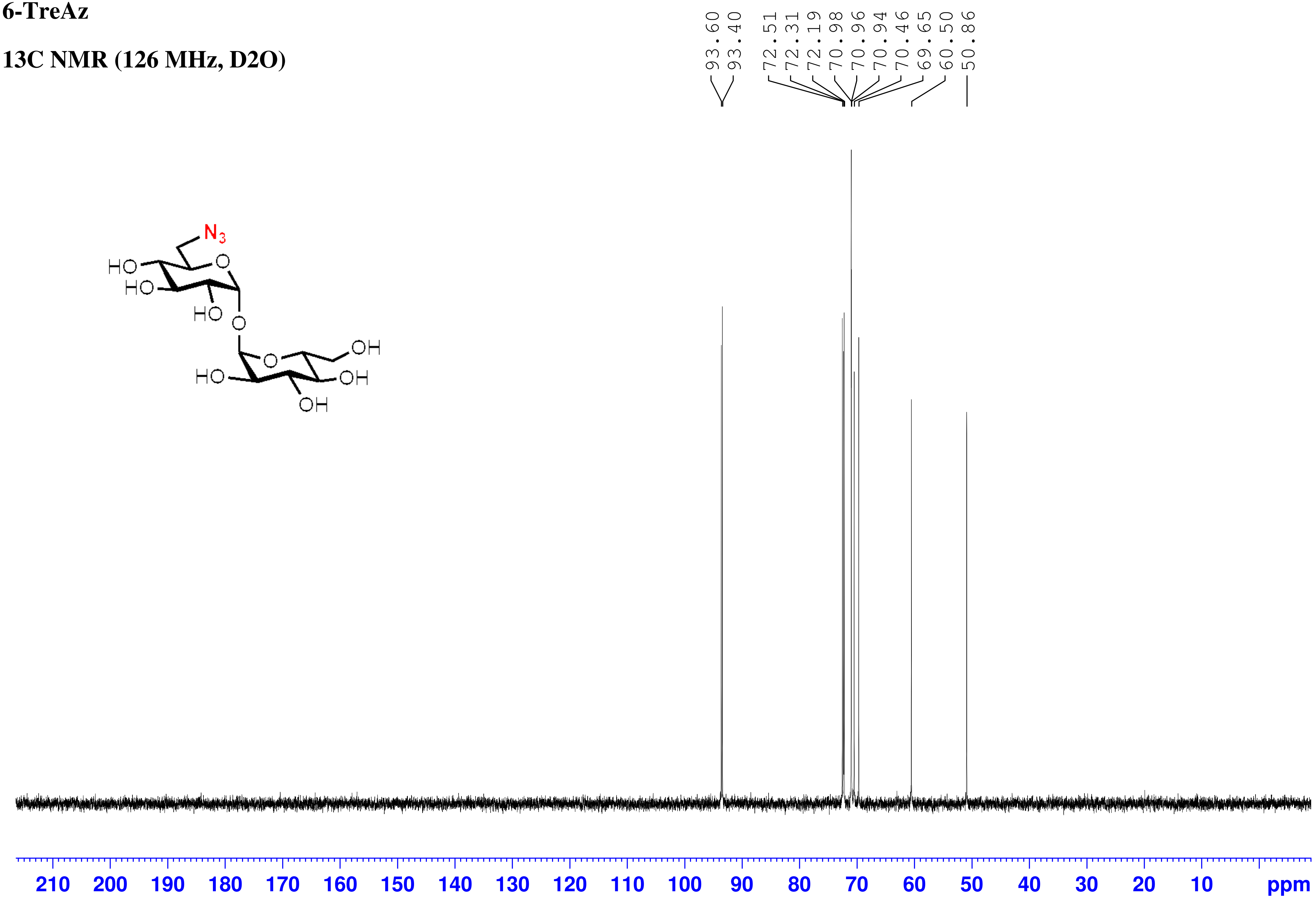

6-TreAz

13C NMR (126 MHz, D2O)

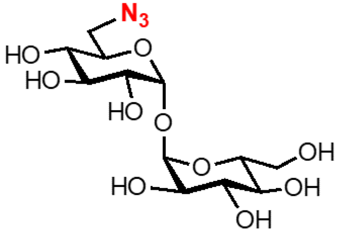

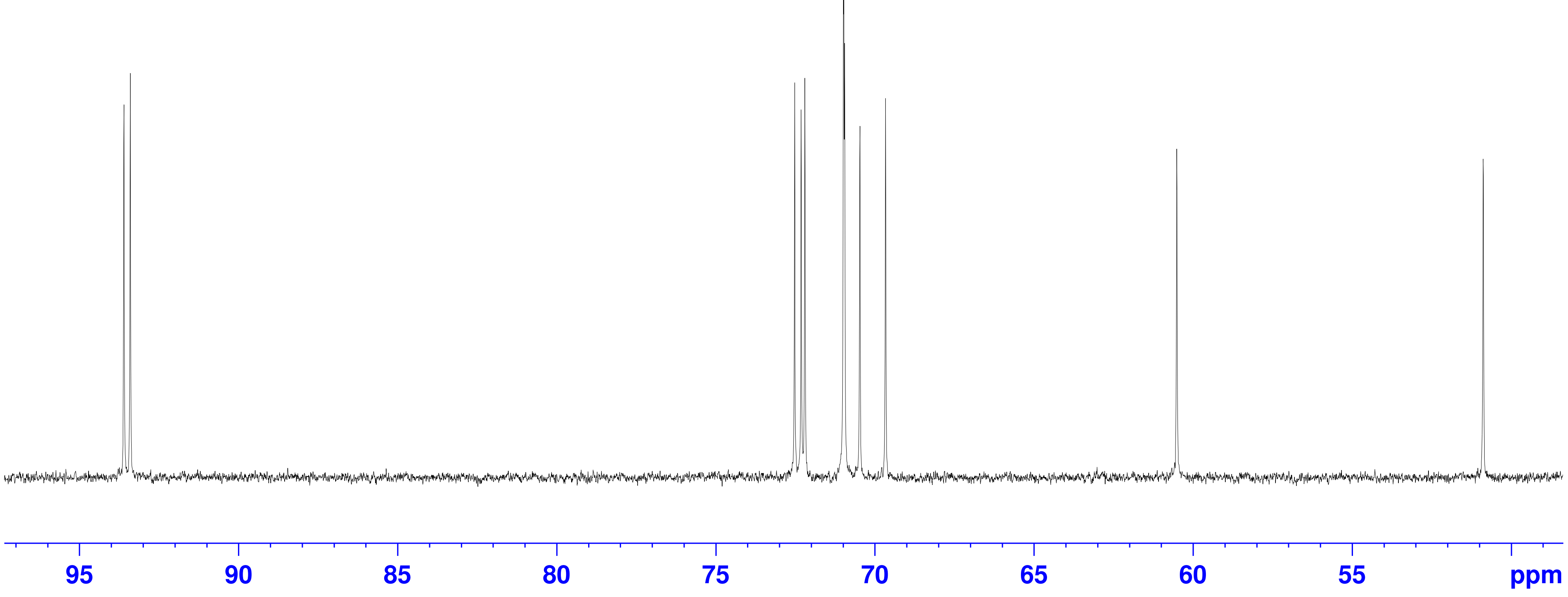

2-Trehalosamine

1H NMR (500 MHz, D2O)

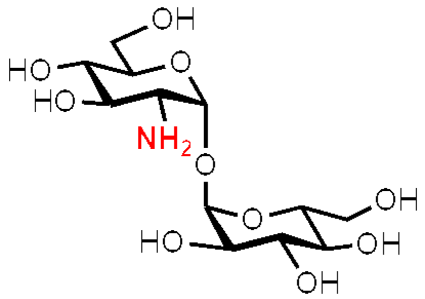

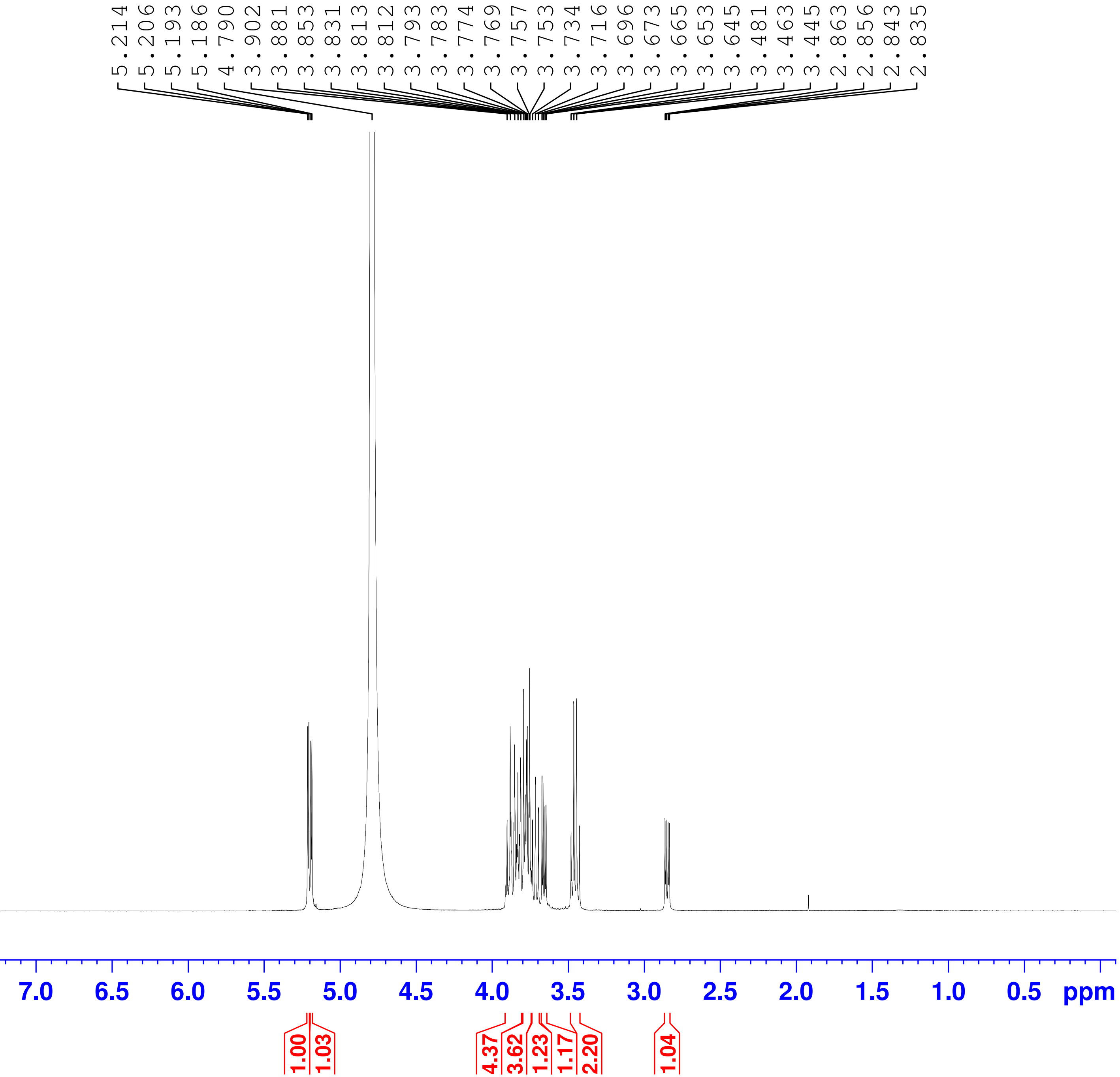

2-TreNH2

1H NMR (500 MHz, D2O)

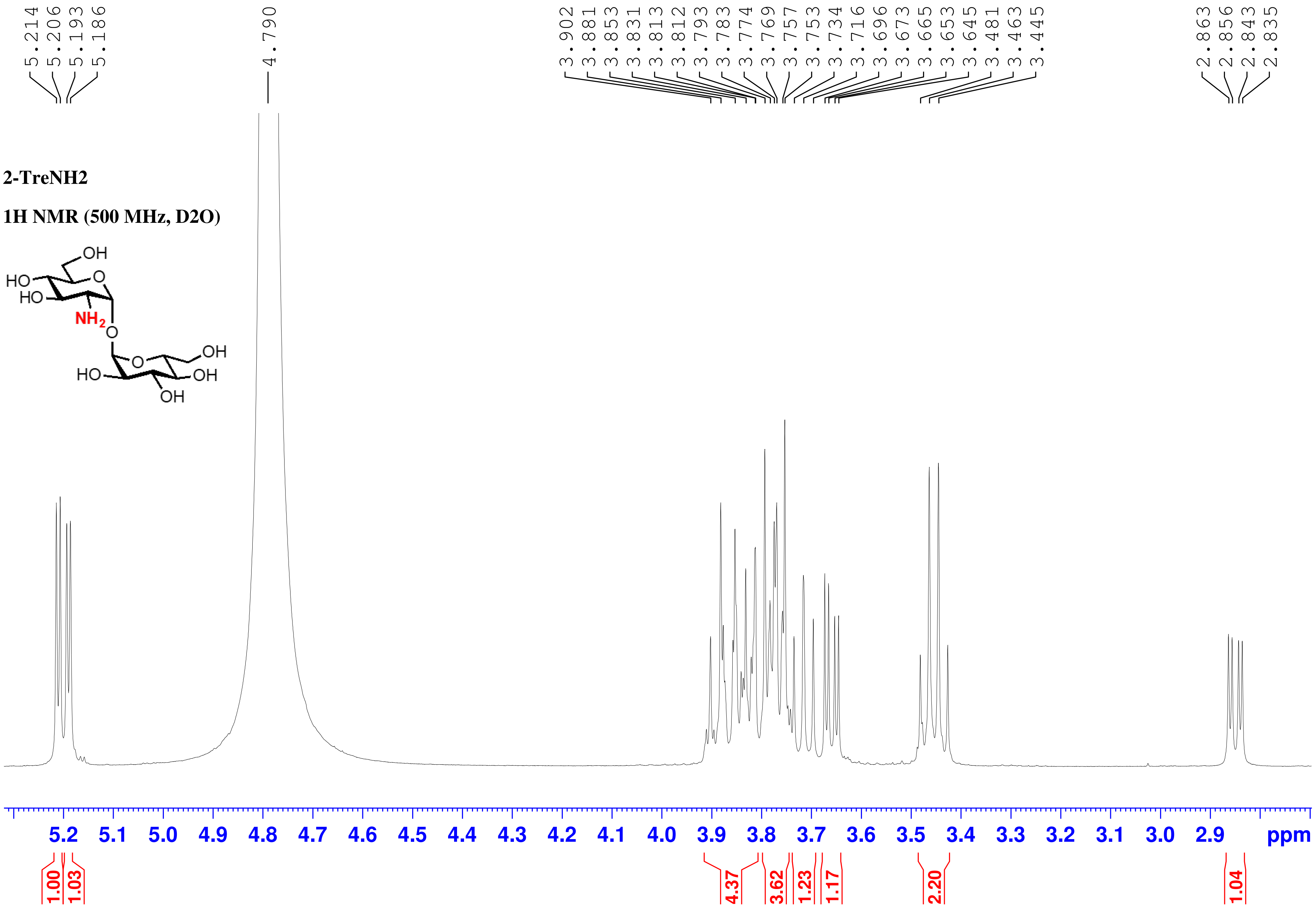

2-TreNH2

13C NMR (126 MHz, D2O)

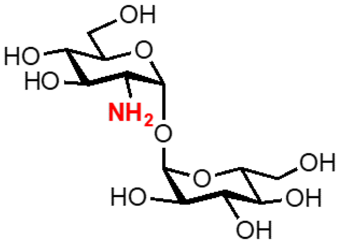

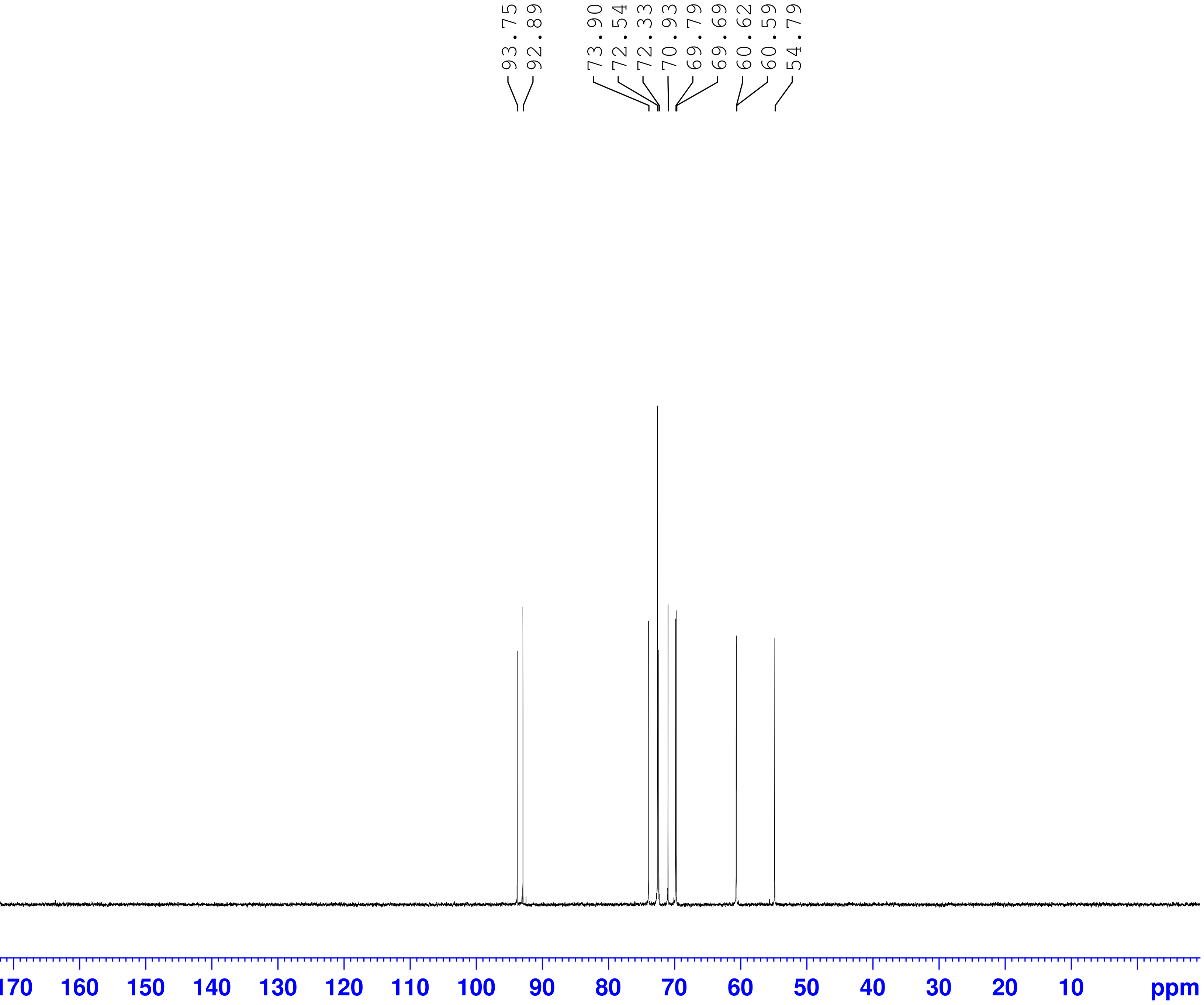

2-TreNH2

13C NMR (126 MHz, D2O)

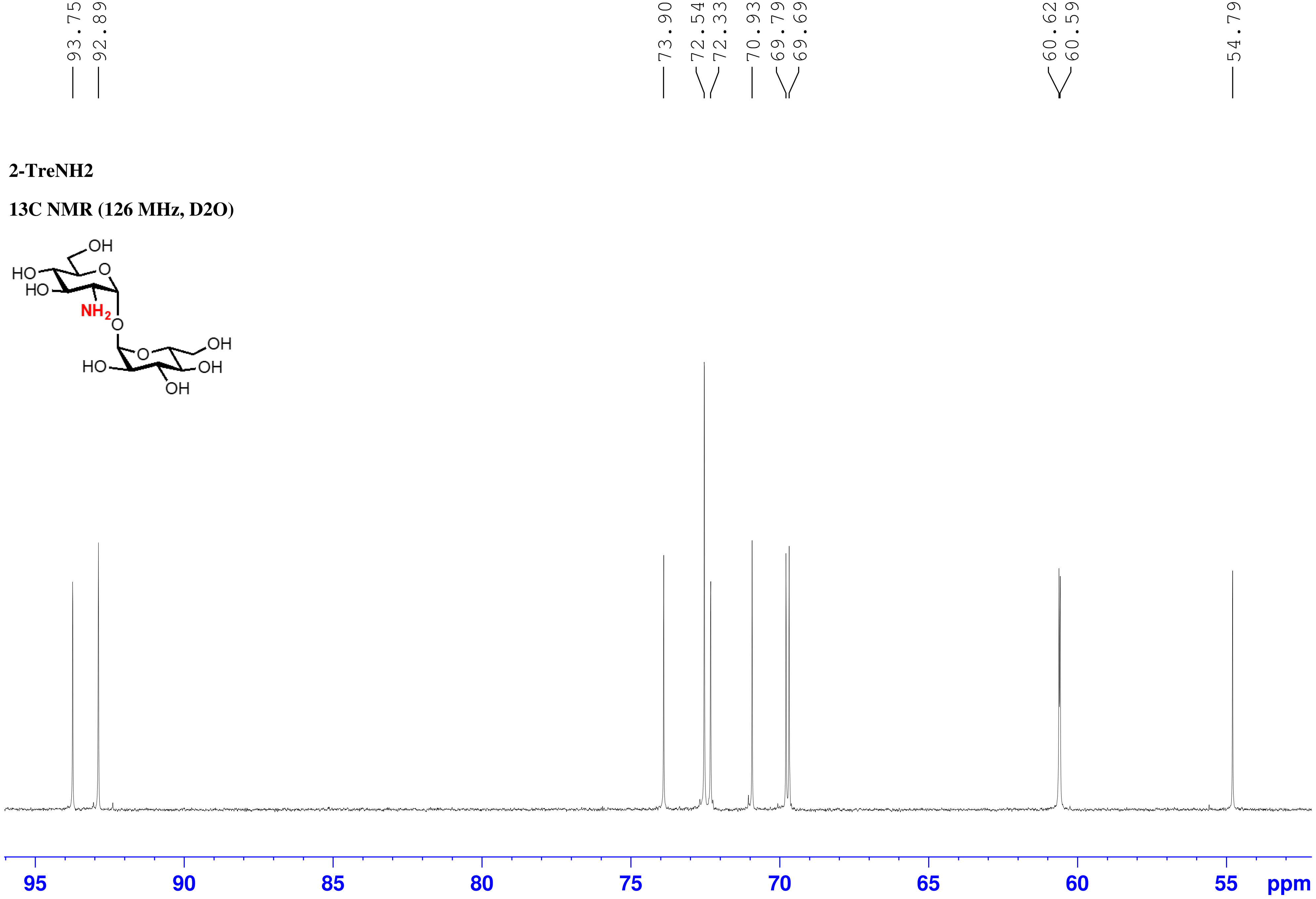

3-Trehalosamine

1H NMR (500 MHz, D2O)

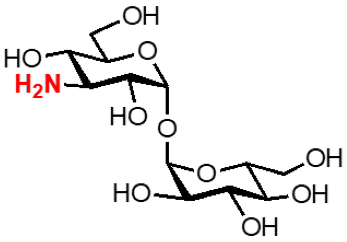

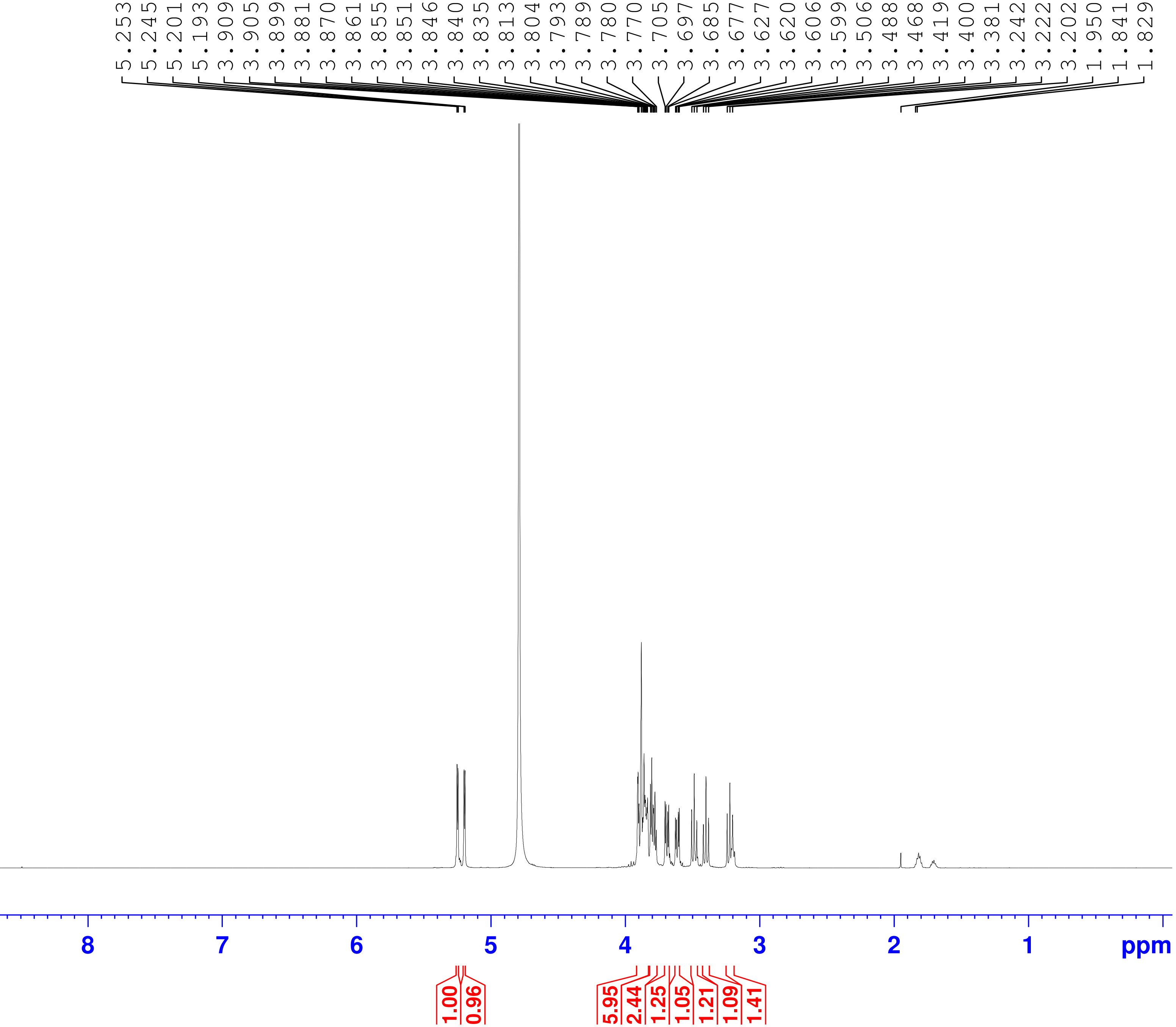

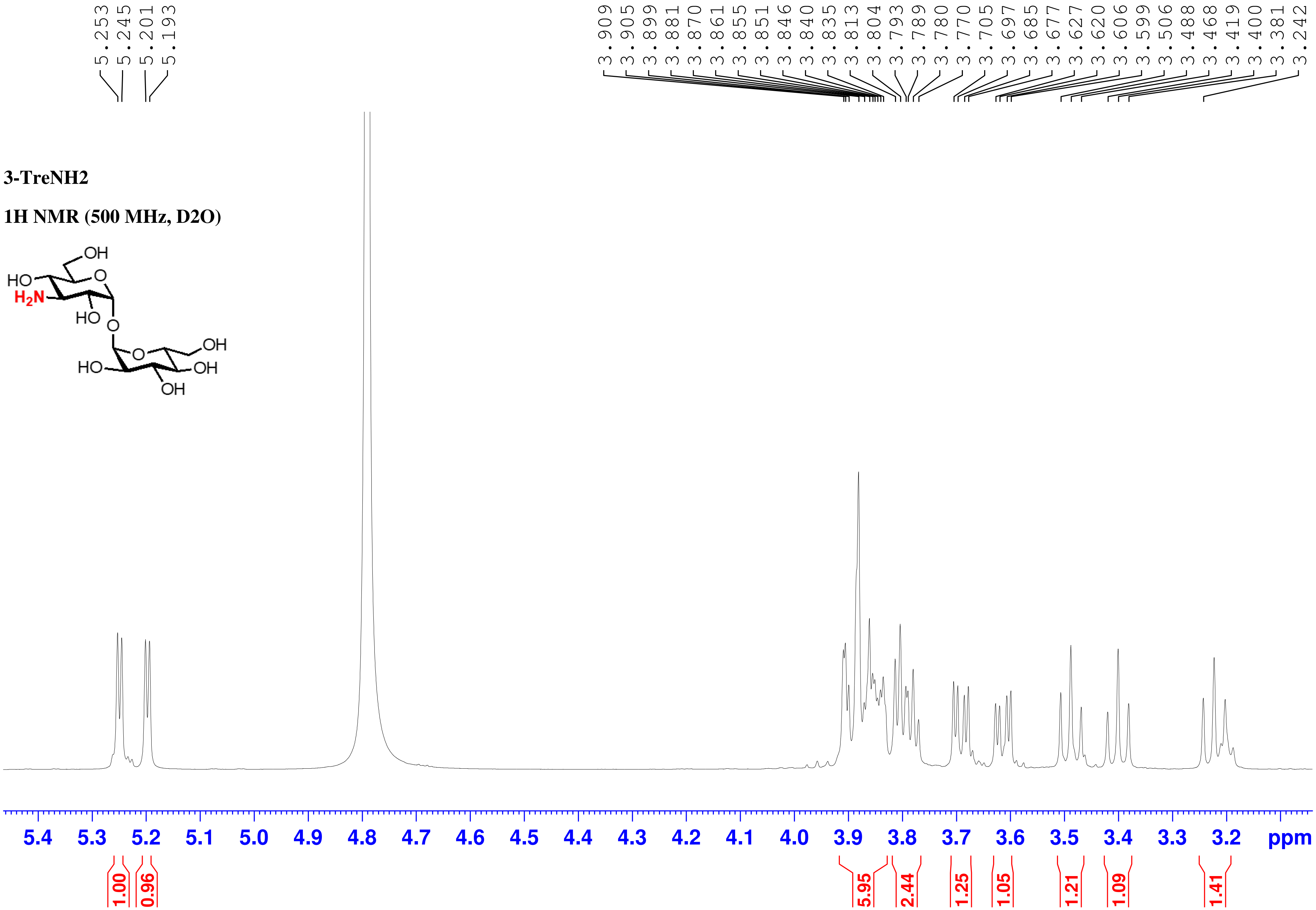
3-TreNH2
1H NMR (500 MHz, D2O)
HO
H2N
HO
O
OH
HO
OH
OH
OH
5.253
5.245
5.201
5.193
3.909
3.905
3.899
3.881
3.870
3.861
3.855
3.851
3.846
3.840
3.835
3.813
3.804
3.793
3.789
3.780
3.770
3.705
3.697
3.685
3.677
3.627
3.620
3.606
3.599
3.506
3.488
3.468
3.419
3.400
3.381
3.242
5.4 5.3 5.2 5.1 5.0 4.9 4.8 4.7 4.6 4.5 4.4 4.3 4.2 4.1 4.0 3.9 3.8 3.7 3.6 3.5 3.4 3.3 3.2 ppm
1.00
0.96
5.95
2.44
1.25
1.05
1.21
1.09
1.41

3-Trehalosamine

13C NMR (126 MHz, D2O)

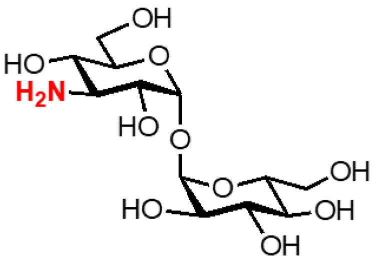

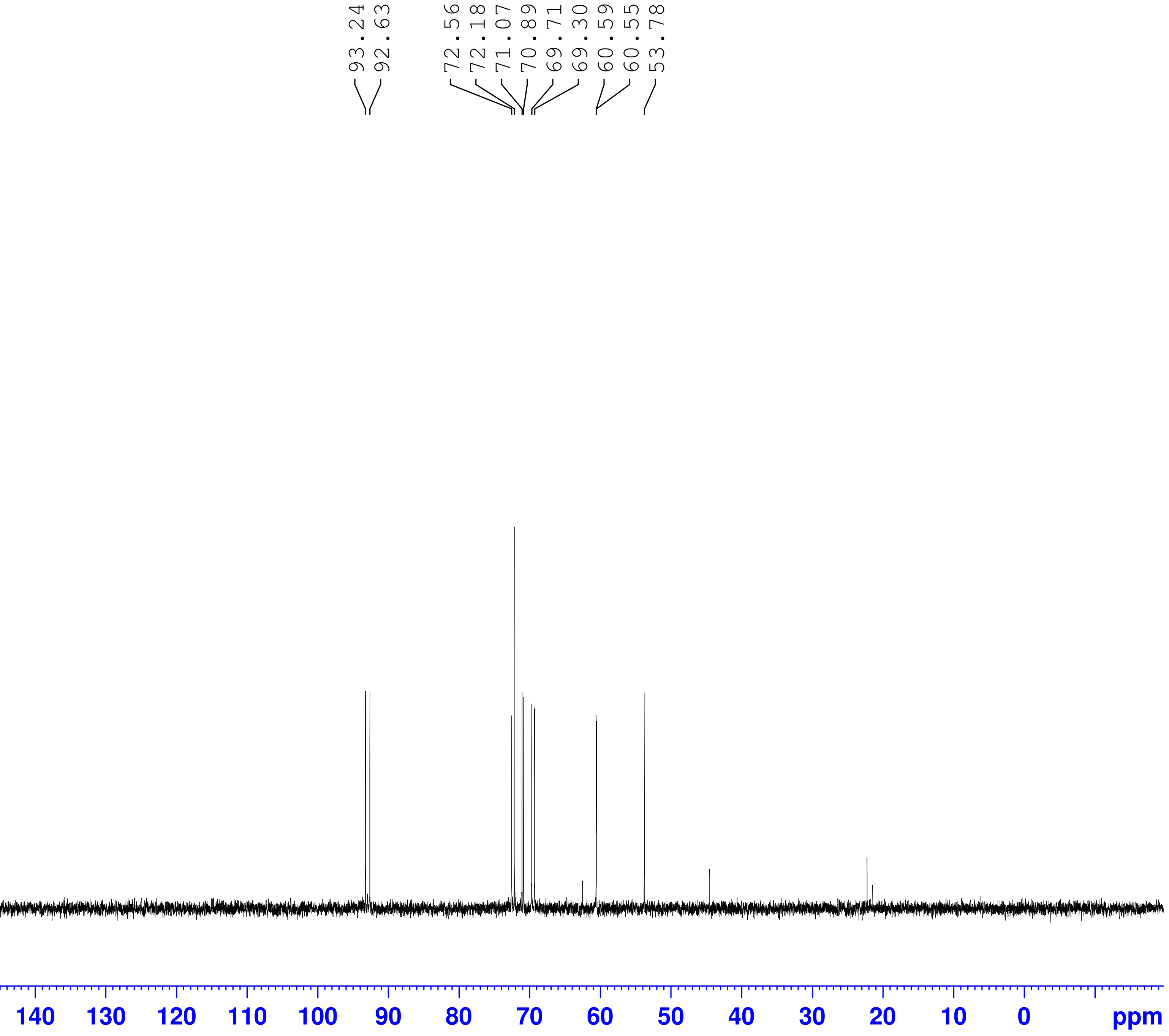

3-TreNH2

13C NMR (126 MHz, D2O)

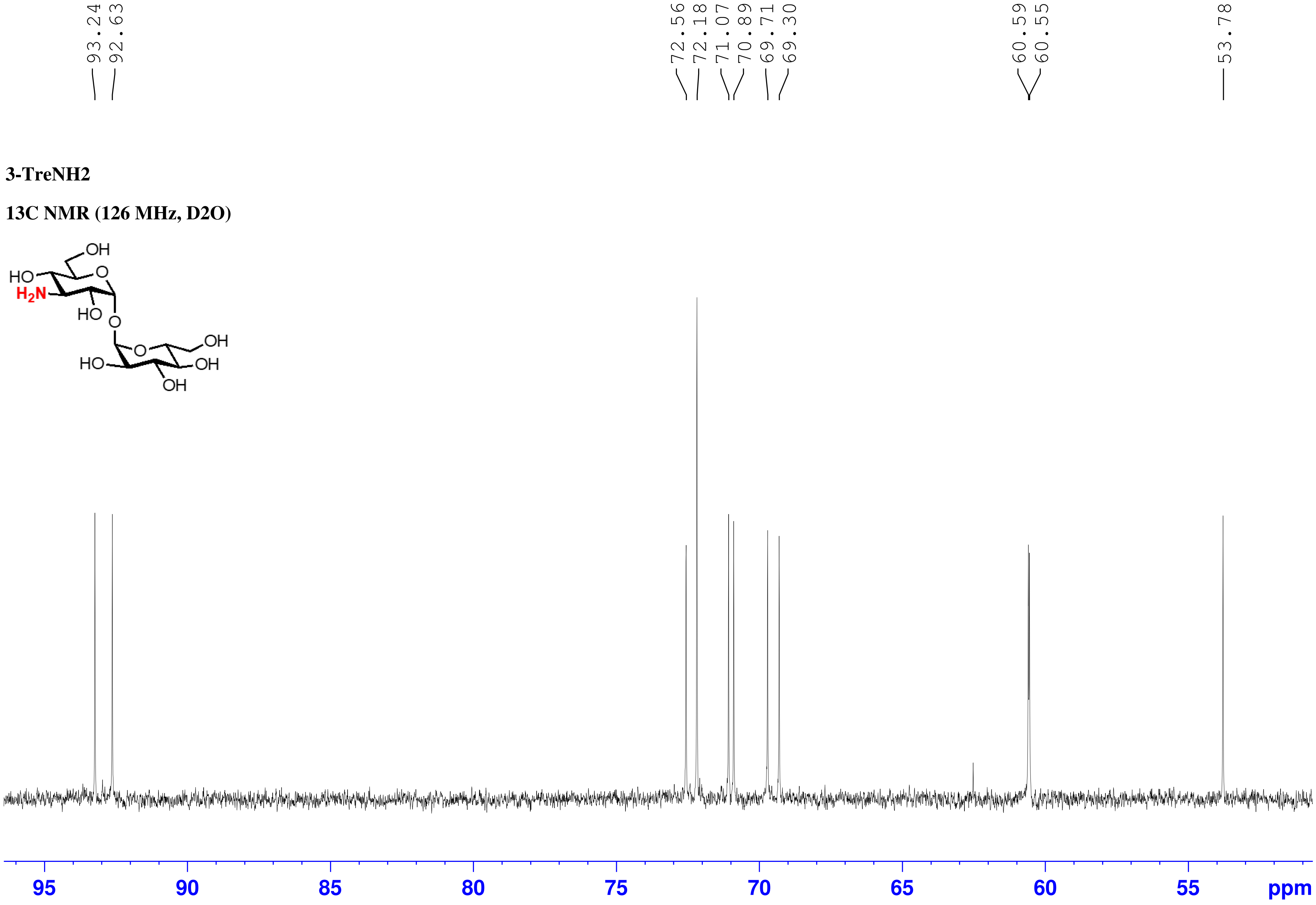

4-TreNH2

1H NMR (500 MHz, D2O)

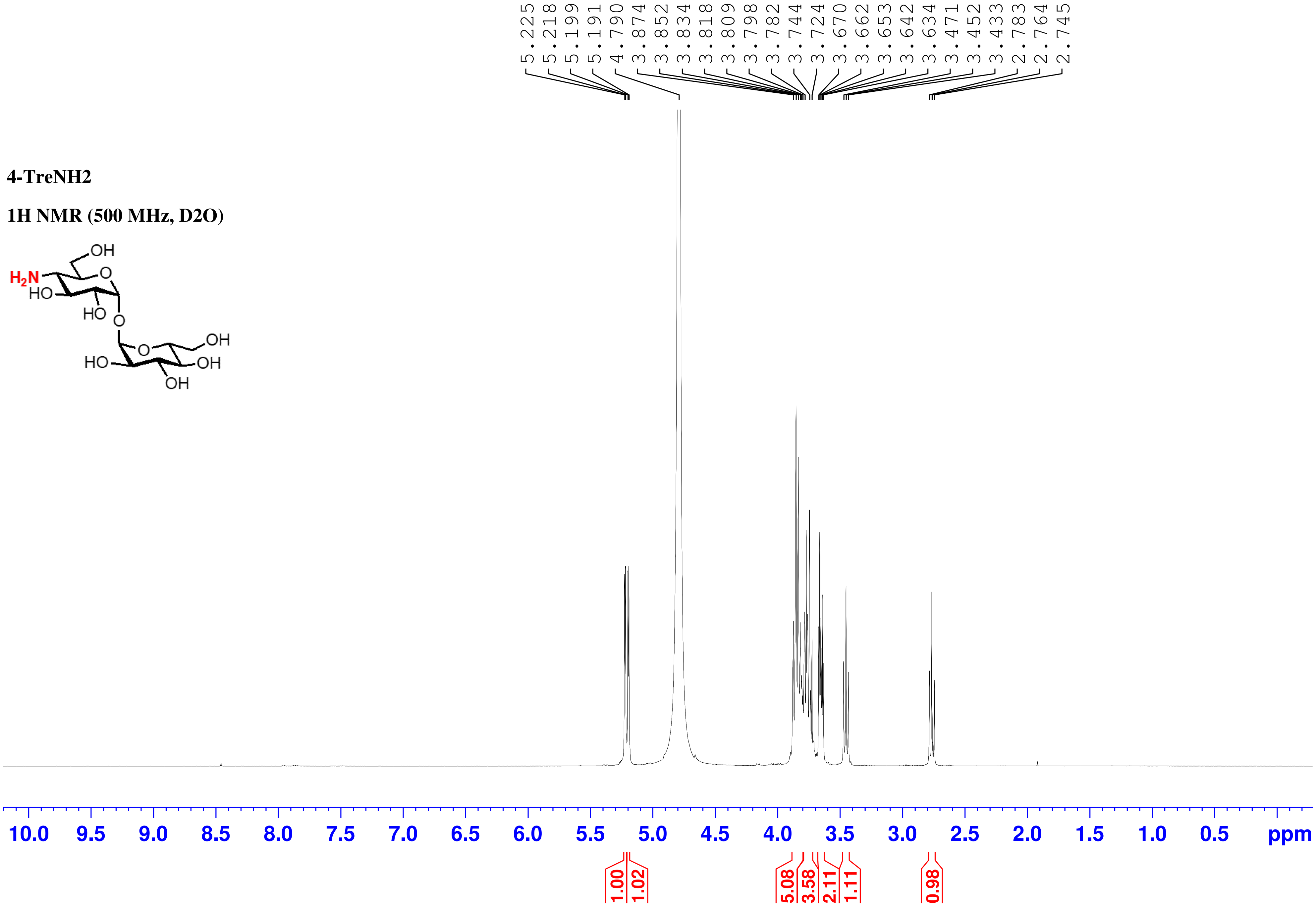

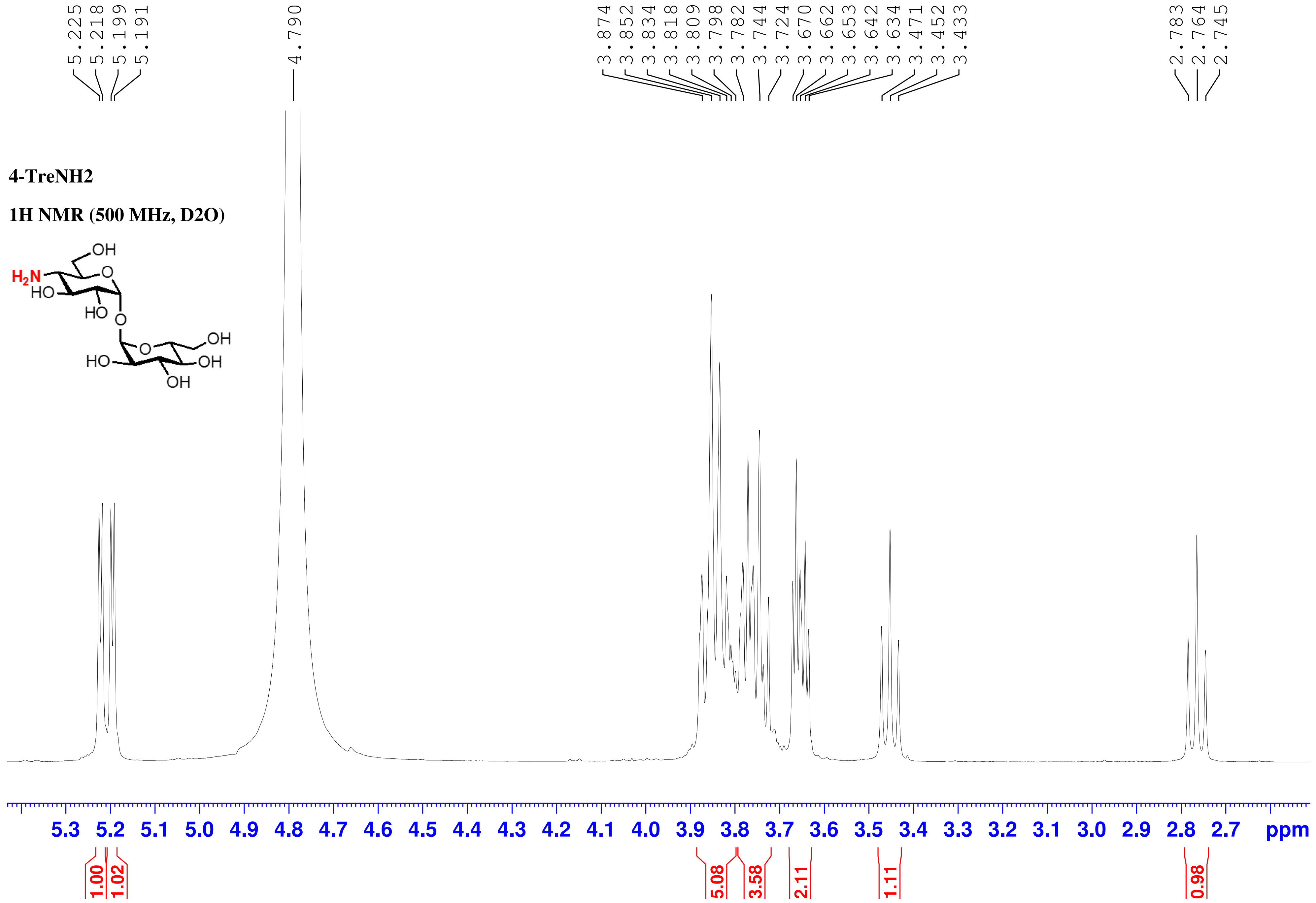

4-TreNH2
1H NMR (500 MHz, D2O)
H2N
OH
O
HO
HO
O
OH
O
HO
OH
OH
5.225
5.218
5.199
5.191
4.790
3.874
3.852
3.834
3.818
3.809
3.798
3.782
3.744
3.724
3.670
3.662
3.653
3.642
3.634
3.471
3.452
3.433
2.783
2.764
2.745
5.3 5.2 5.1 5.0 4.9 4.8 4.7 4.6 4.5 4.4 4.3 4.2 4.1 4.0 3.9 3.8 3.7 3.6 3.5 3.4 3.3 3.2 3.1 3.0 2.9 2.8 2.7 ppm
1.00
1.02
5.08
3.58
2.11
1.11
0.98

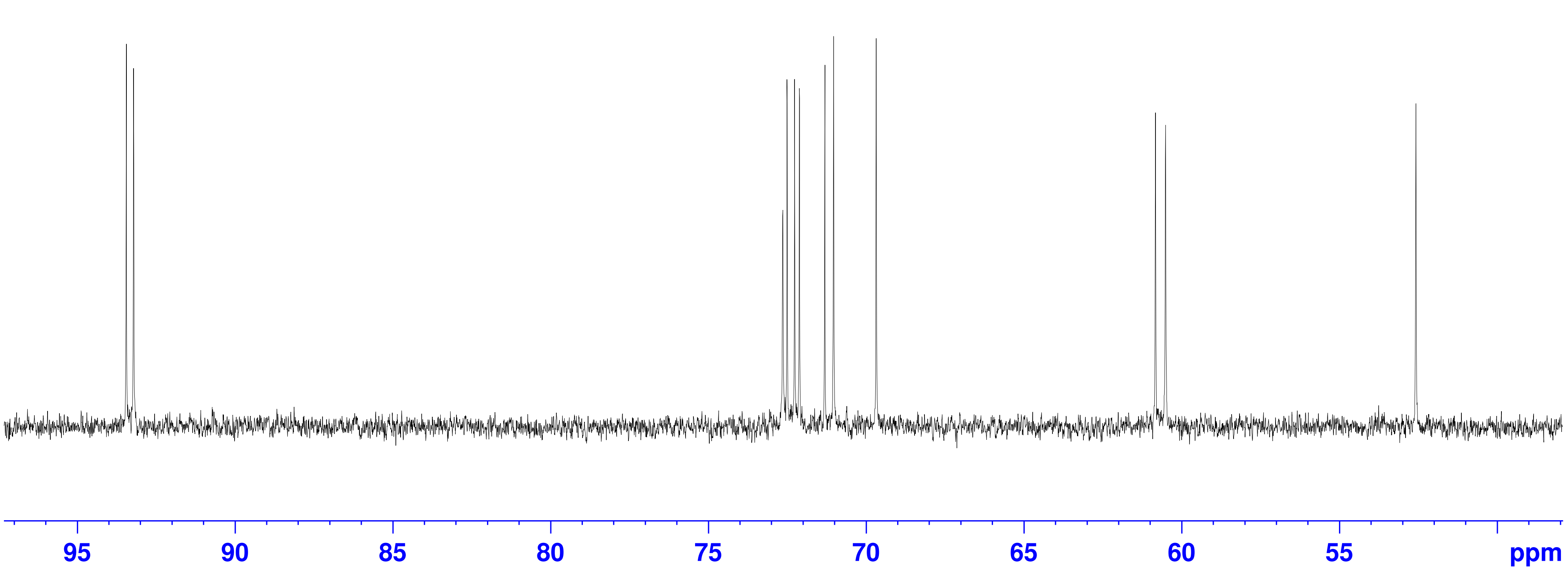

4-TreNH2

13C NMR (126 MHz, D2O)

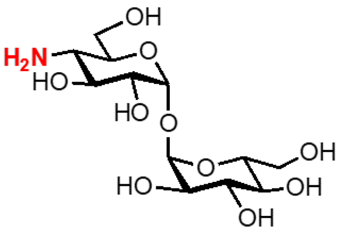

4-TreNH2

13C NMR (126 MHz, D2O)

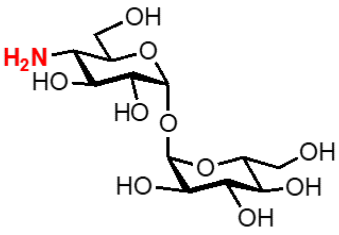

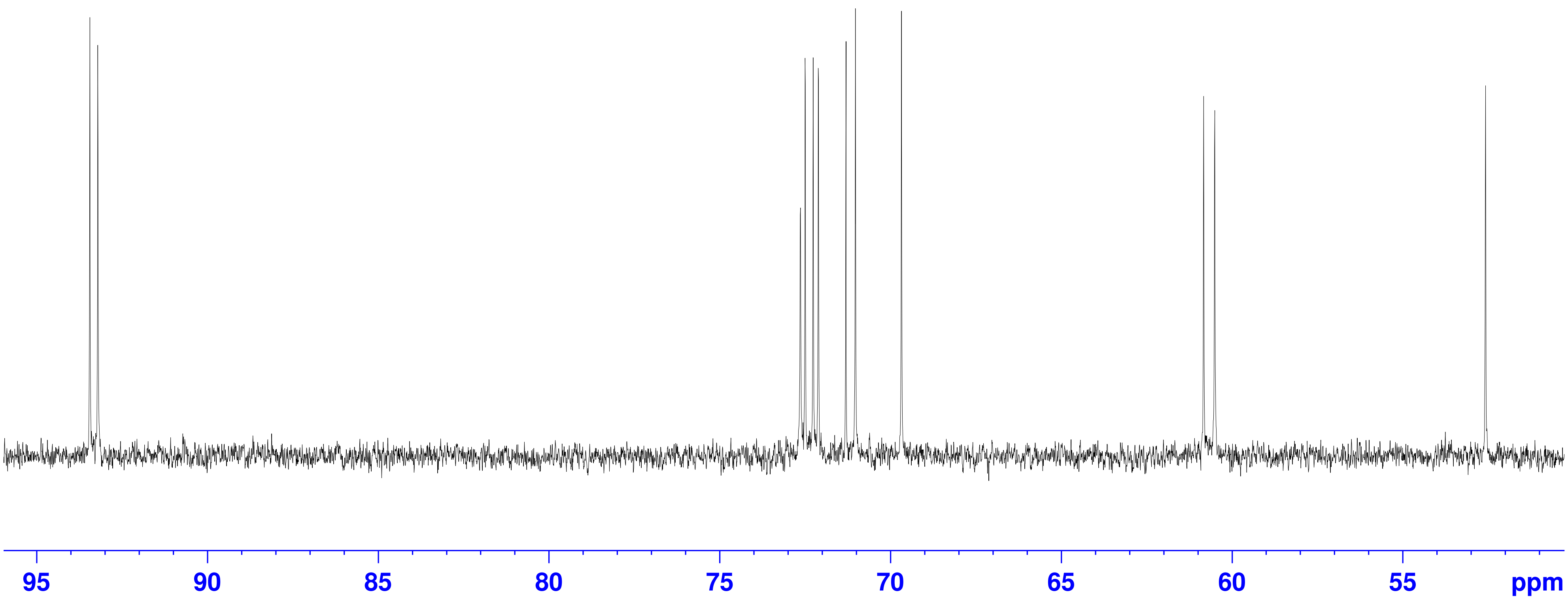

6-Trehalosamine acetate salt

1H NMR (500 MHz, D2O)

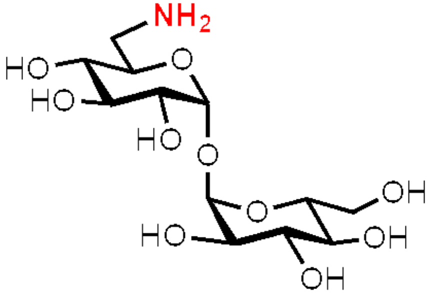

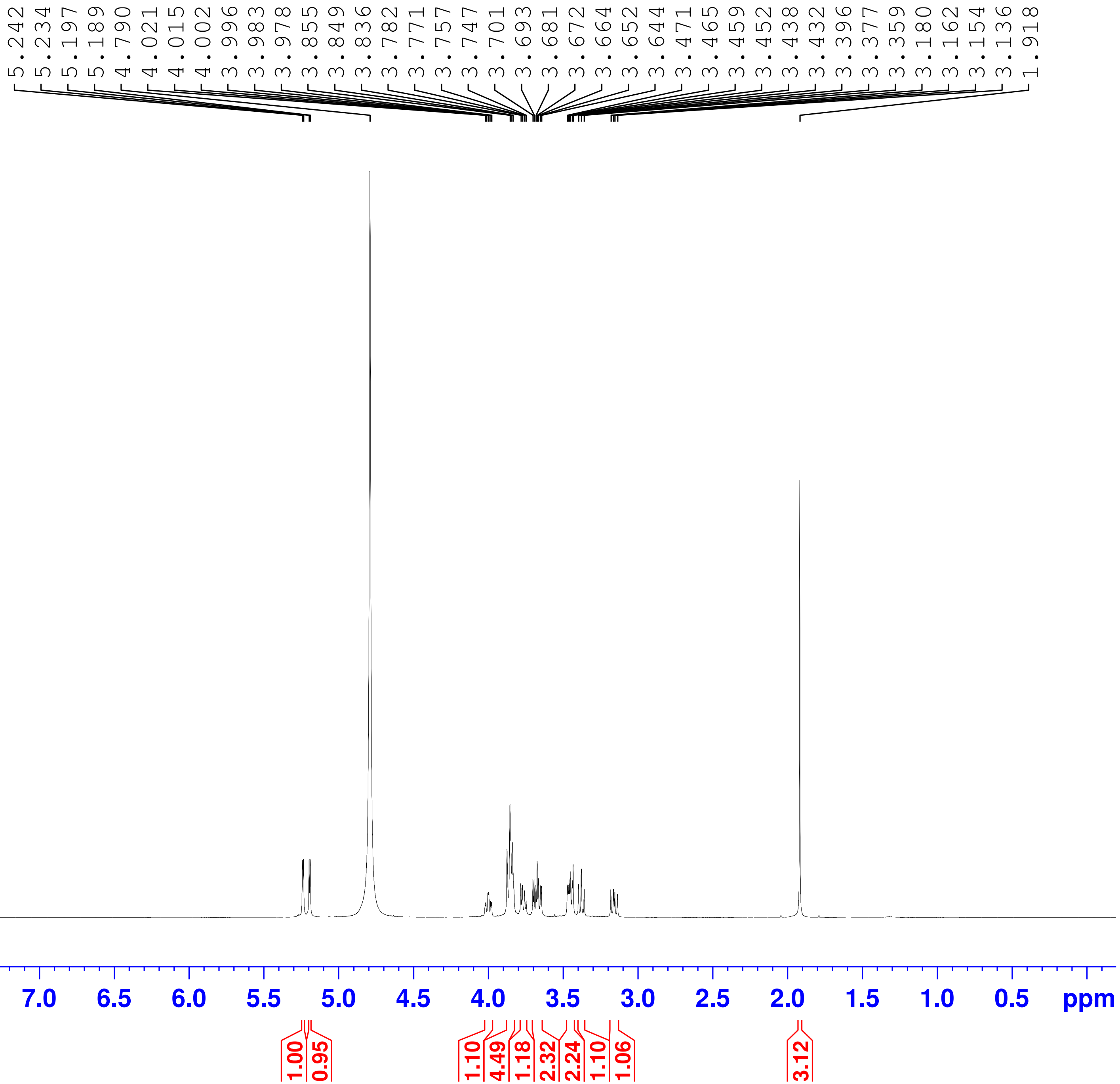

6-TreNH2

1H NMR (500 MHz, D2O)

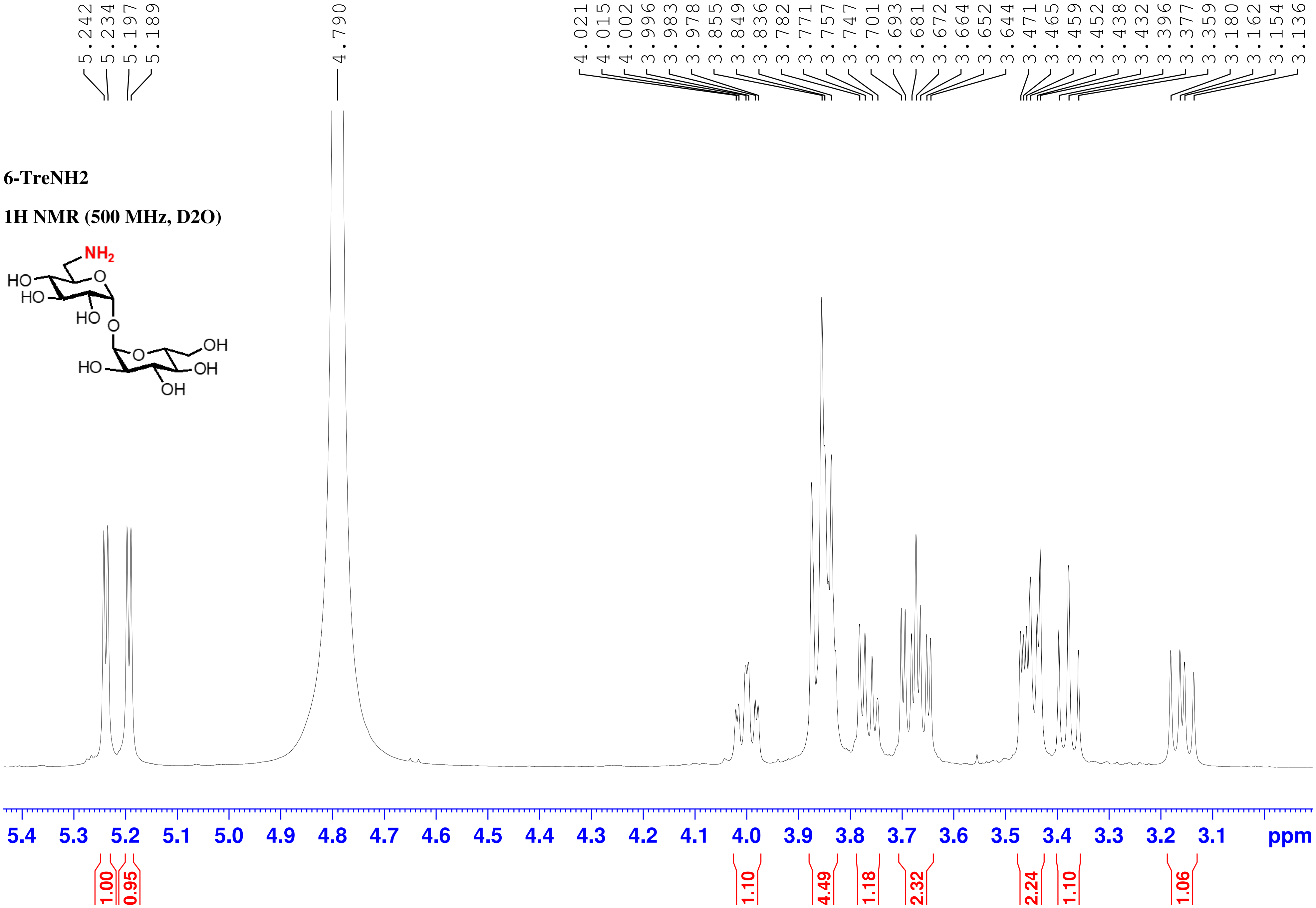

6-Trehalosamine acetate salt

13C NMR (126 MHz, D2O)

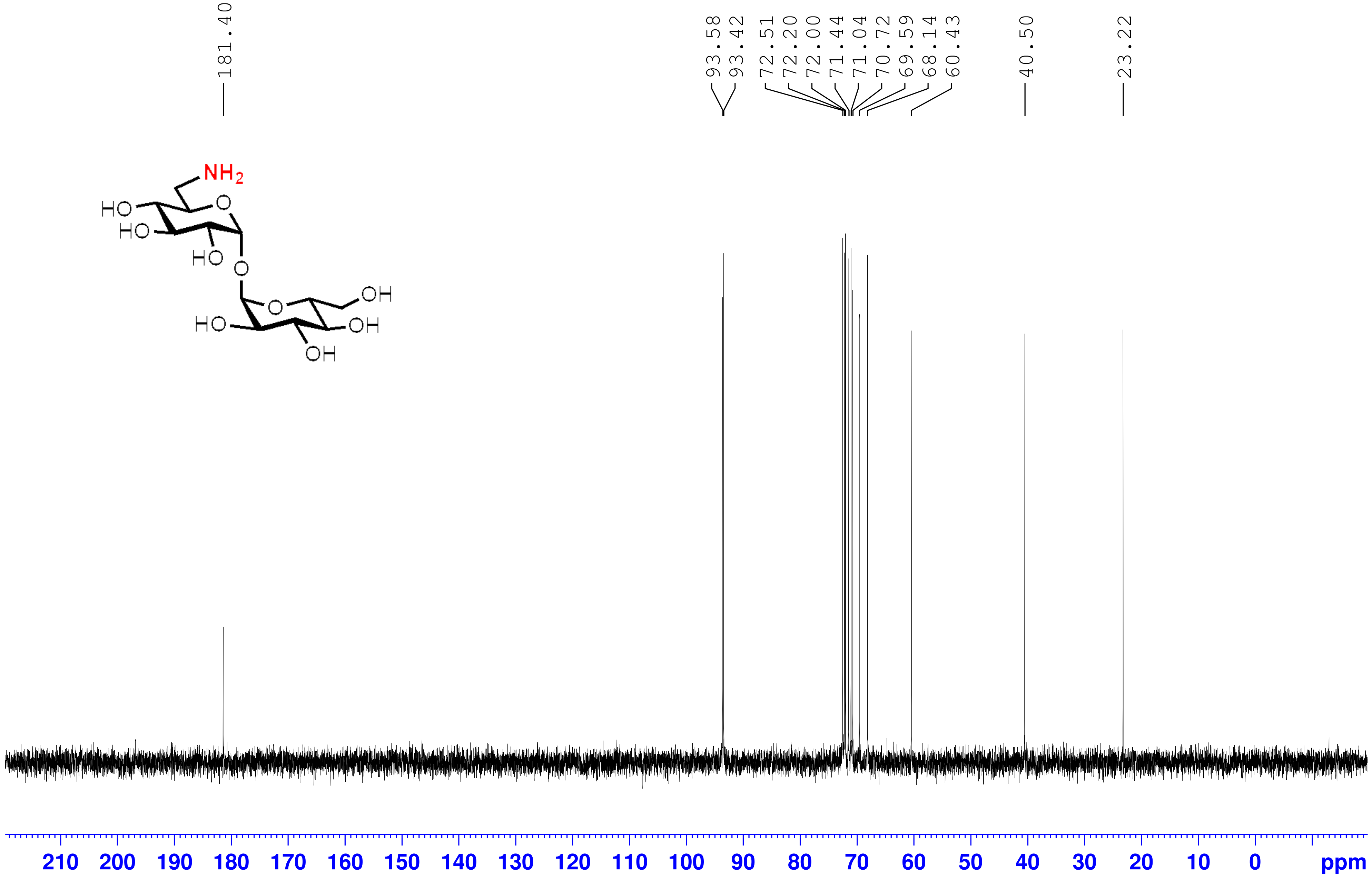

6-TreNH2

13C NMR (126 MHz, D2O)

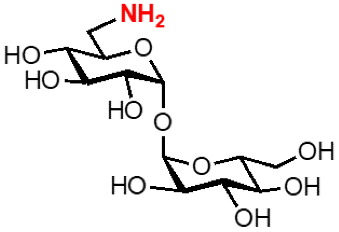

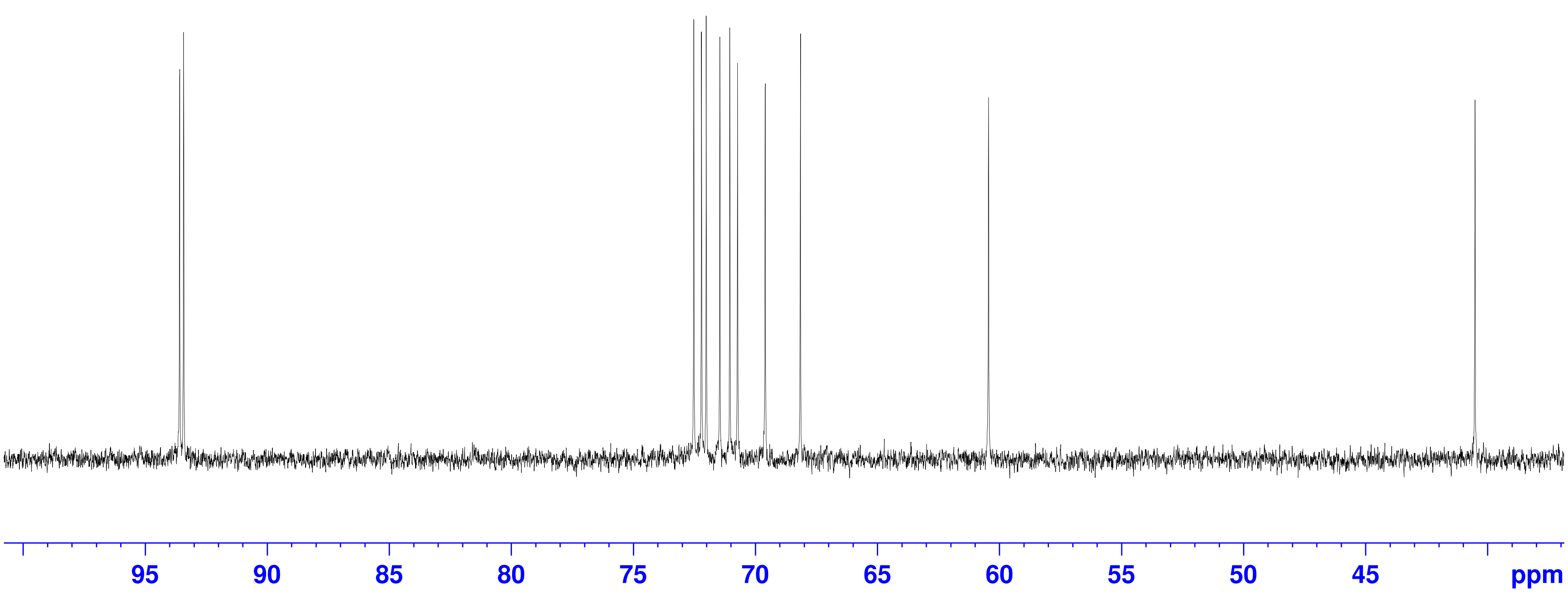

Supplement: Supplementary file 1 — id4c00138_si_001.pdf [file id4c00138_si_001.pdf]
